# Supplementary material for: Loss of CHD1 Promotes Heterogeneous Mechanisms of Resistance to AR-Targeted Therapy via Chromatin Dysregulation
Source: Cancer Cell. 2020 Apr 13;37(4):584–598.e11. doi: 10.1016/j.ccell.2020.03.001 (PMC7292228; doi:10.1016/j.ccell.2020.03.001)
Supplement: Document S1. Figures S1–S8 [file mmc1.pdf]

Cancer Cell, Volume *37*

Supplemental Information

Loss of *CHD1* Promotes Heterogeneous Mechanisms of Resistance to AR-Targeted Therapy via Chromatin Dysregulation

Zeda Zhang, Chuanli Zhou, Xiaoling Li, Spencer D. Barnes, Su Deng, Elizabeth Hoover, Chi-Chao Chen, Young Sun Lee, Yanxiao Zhang, Choushi Wang, Lauren A. Metang, Chao Wu, Carla Rodriguez Tirado, Nickolas A. Johnson, John Wongvipat, Kristina Navrazhina, Zhen Cao, Danielle Choi, Chun-Hao Huang, Eliot Linton, Xiaoping Chen, Yupu Liang, Christopher E. Mason, Elisa de Stanchina, Wassim Abida, Amaia Lujambio, Sheng Li, Scott W. Lowe, Joshua T. Mendell, Venkat S. Malladi, Charles L. Sawyers, and Ping Mu

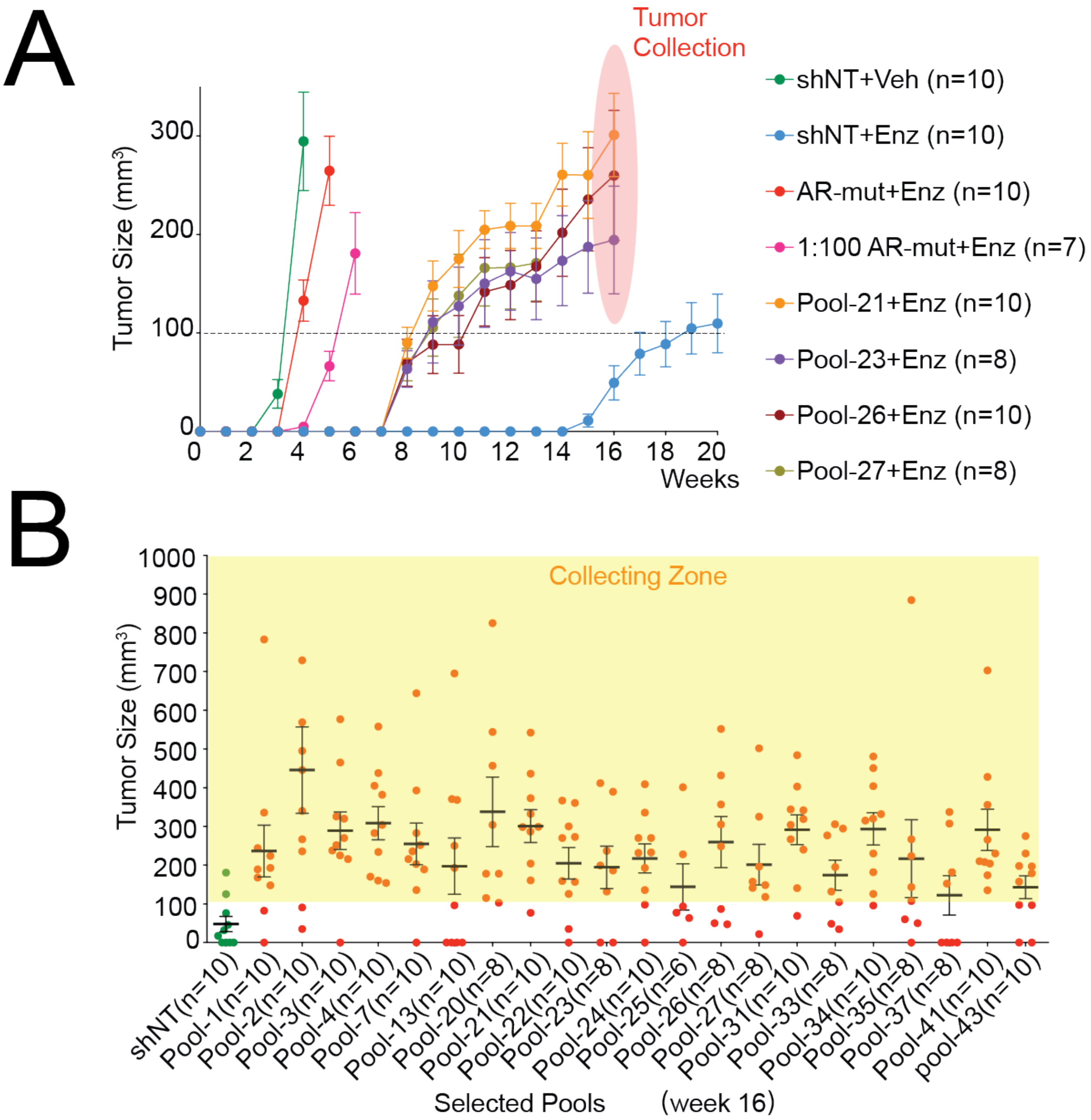

Figure S1 related to Figure 1. Pilot experiments and the validation of other two top hits emerged from the *in vivo* library screen. (A) Tumor growth curve of xenografted LNCaP/AR cells transduced with annotated plasmids or pools of library. Enz denotes enzalutamide treatment at 10 mg/kg orally one day after grafting. Veh denotes 0.5% CMC + 0.1% Tween 80 treatment at same dosage. (B) Tumor measurement of xenografted LNCaP/AR cells transduced with shNT and 21 representative pools at week 16. For all panels unless otherwise noted, mean ± SEM. is represented.

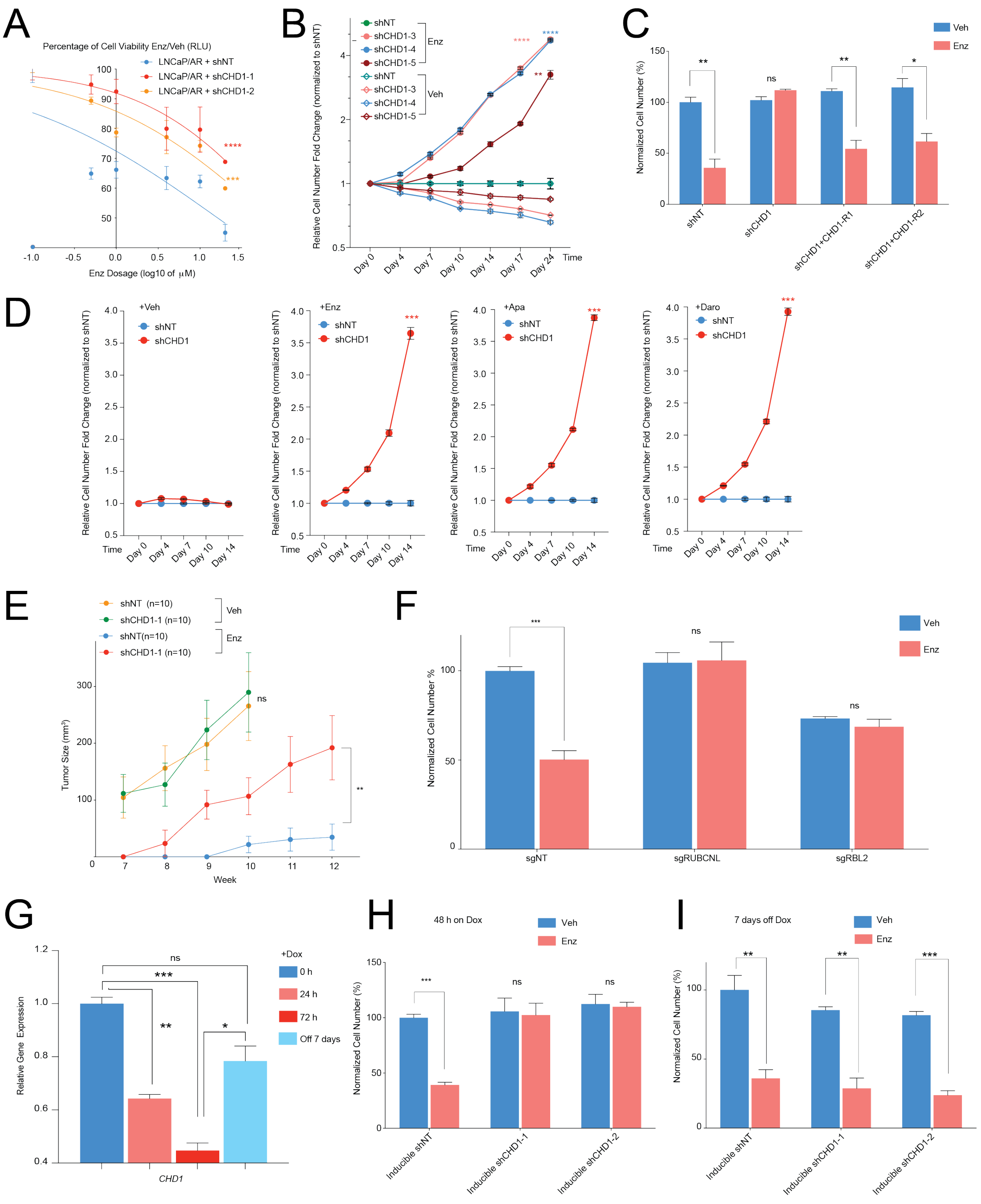

Figure S2 related to Figure 3. Resistance conferred by *CHD1* KD is reversible. (A) Dose response curve of LNCaP/AR cells transduced with annotated shRNAs. Mean ± SEM is represented, and p values were calculated by non-linear regression with extra sun-of-squares F test, 3 biological replicates were used for each data point. (B) Relative cell number fold change compared to shNT group, based on the results of FACS-based competition assay. Enz denotes

enzalutamide of 10 μM and Veh denotes DMSO. Mean ± SEM is represented, and p values were calculated using two-way ANOVA, 3 biological replicates in each group. (C) Relative cell number of LNCaP/AR cells transduced with annotated shRNAs and rescue plasmids expressing CHD1 cDNA (R1/R2), normalized to shNT+Veh group. Cells were treated with 10 μM enzalutamide (Enz) or DMSO (Veh) for 7 days and cell numbers were counted. Mean ± SEM is represented, and p values were calculated using multiple t tests, 3 biological replicates in each group. (D) Relative cell number fold change compared to shNT group, based on the results of competition assay. Veh denotes DMSO. Enzalutamide (Enz), apalutamide (Apa) and darolutamide (Daro) all denotes dosage of 10 μM. Mean ± SEM is represented, and p values were calculated using multiple t tests, 3 biological replicates in each group. (E) Tumor growth curve of xenografted LNCaP/AR cells transduced with annotated shRNAs. Enz denotes 10 mg/kg orally one day after grafting. Veh denotes 0.5% CMC + 0.1% Tween 80 at same dosage. Mean ± SEM is represented, and p values were calculated using two-way ANOVA. Experiments have been done with two independent repeats. (F) Relative cell number of LNCaP/AR cells transduced with annotated guide RNAs, normalized to shNT+Veh group. Cells were treated with 10 μM enzalutamide (Enz) or DMSO (Veh) for 7 days and cell numbers were counted. Mean ± SEM is represented, and p values were calculated using multiple t tests, 3 biological replicates in each group. (G) Relative gene expression level of *CHD1* in LNCaP/AR cells transduced with annotated inducible shRNAs at various time points. Mean ± SEM is represented, p values were calculated by one-way ANOVA, compared to 0 hr condition, 3 technical replicates in each group. (H) Relative cell number of LNCaP/AR cells transduced with annotated shRNAs in an inducible vector system, normalized to shNT+Veh. Cells were treated with 250 ng/ml doxycycline (Dox) for 48 hours, and then treated with 7 days of 10 μM enzalutamide (Enz) or DMSO (Veh), and cell numbers were counted. Mean ± SEM is represented, and p values were calculated using multiple t tests, 3 biological replicates in each group. (I) Relative cell number of LNCaP/AR cells transduced with annotated shRNAs in an inducible vector system, normalized to shNT+Veh. Cells were treated with 250 ng/ml doxycycline (Dox) for 48 hours, removed doxycycline for 7 days, and then treated with 7 days of 10 μM enzalutamide (Enz) or DMSO (Veh), then cell numbers were counted. Mean ± SEM is represented, and p values were calculated using multiple t tests, 3 biological replicates in each group. For all panels, **** $p<0.0001$. *** $p<0.001$. ** $p<0.01$. * $p<0.05$.

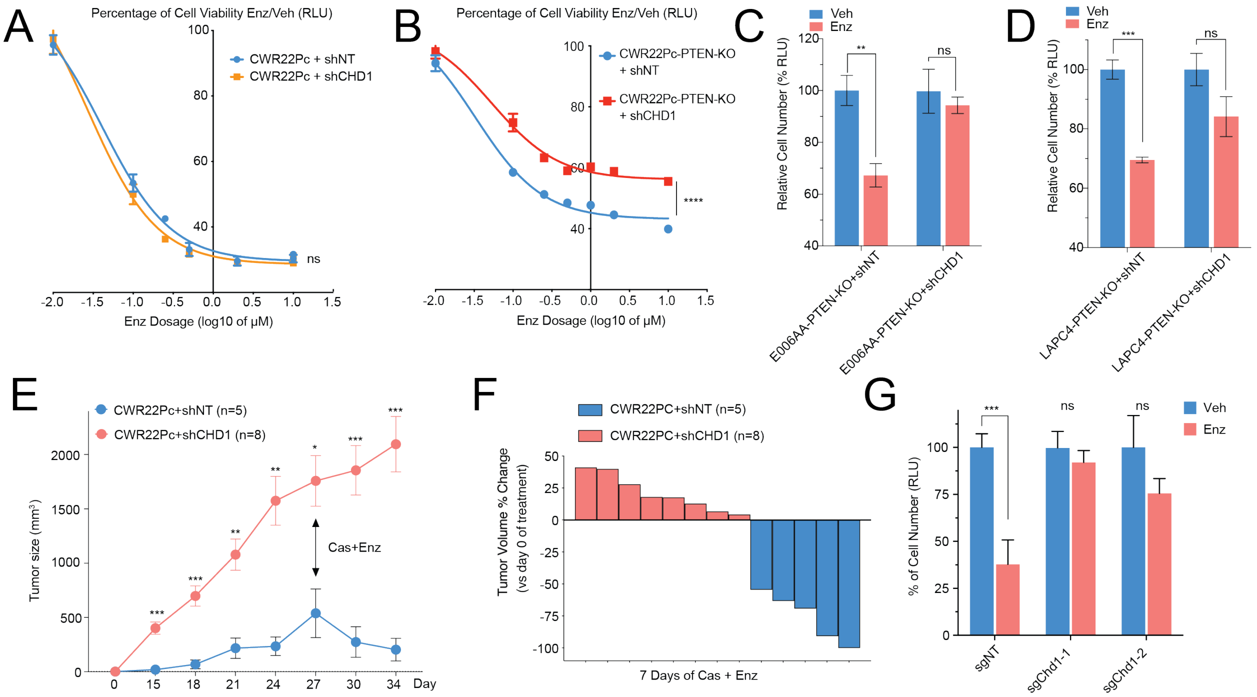

Figure S3 related to Figure 3. *CHD1* KD confers resistance in other PCa models. (A) Enzalutamide (Enz) dose response curve of CWR22Pc cells transduced with annotated shRNAs. Mean ± SEM is represented, and p values were calculated by non-linear regression with extra sun-of-squares F test, 3 biological replicates were used for each data point. (B) Enzalutamide (Enz) response curve of CWR22Pc-PTEN-KO cells transduced with annotated shRNAs. Mean ± SEM is represented, and p values were calculated by non-linear regression with extra sun-of-squares F test, 3 biological replicates were used for each data point. (C) Relative cell number of E006AA-PTEN-KO cells transduced with annotated hairpins. Cells were treated with 15 µg/ml enzalutamide (Enz) or DMSO (Veh) for 6 days in 3D Matrigel and cell number was measured using CellTiter-Glo assay. Mean ± SEM is represented, and p values were calculated using multiple t tests, 3 biological replicates in each group. (D) Relative cell number of LAPC4-PTEN-KO cells transduced with annotated hairpins. Cells were treated with 30 µg/ml enzalutamide (Enz) or DMSO (Veh) for 6 days and cell number was measured using CellTiter-Glo assay. Mean ± SEM is represented, and p values were calculated using multiple t tests, 3 biological replicates in each group. (E) Tumor growth curve of xenografted CWR22Pc cells in intact mice. All animals were castrated (Cas) and treated with enzalutamide (Enz) at 10 mg/kg orally from day 27. Mean ± SEM is represented and p values were calculated using two-way ANOVA. (F) Waterfall plot displaying changes in tumor size of xenografted CWR22Pc cells after 1 week of castration and enzalutamide treatments. Cas denotes castration. Enz denotes enzalutamide treatment at 10 mg/kg orally. (G) Relative cell number of mouse organoid (*Pten*$^{-/-}$) cultured in 3D. Organoids were treated with DMSO (Veh) or 1 µM enzalutamide (Enz) for 6 days. Mean ± SEM is represented, and p values were calculated using multiple t tests, 3 biological replicates in each group. For all panels, **** $p<0.0001$. *** $p<0.001$. ** $p<0.01$. * $p<0.05$.

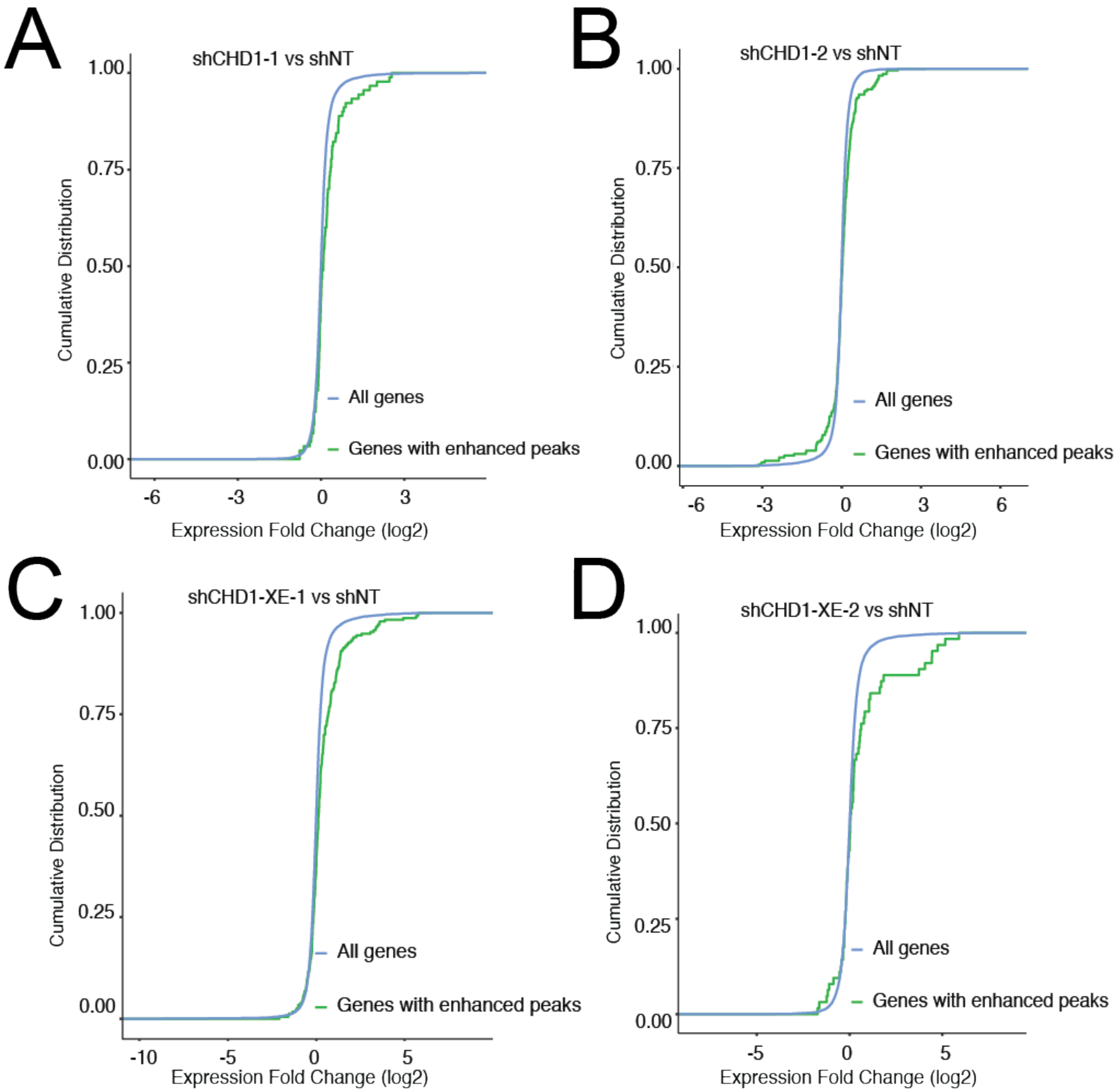

Figure S4 related to Figure 5. ATAC-seq analysis reveals global changes in chromatin accessibility after *CHD1* loss. (A-D) Cumulative distribution of log2 expression changes in shCHD1-1 cell line compared to shNT(A); shCHD1-2 cell line compared to shNT (B); shCHD1-XE-1 cell line compared to shNT (C); shCHD1-XE-2 cell line compared to shNT (D). For all panels, the blue line denotes all of the expressed genes. Green line denotes the genes with significant upregulated ATAC-peaks compared to shNT (enhanced peaks, combined increasing of peaks>6). For all panels, reads from 3 biological replicates were pooled to calculated the consensus peaks.

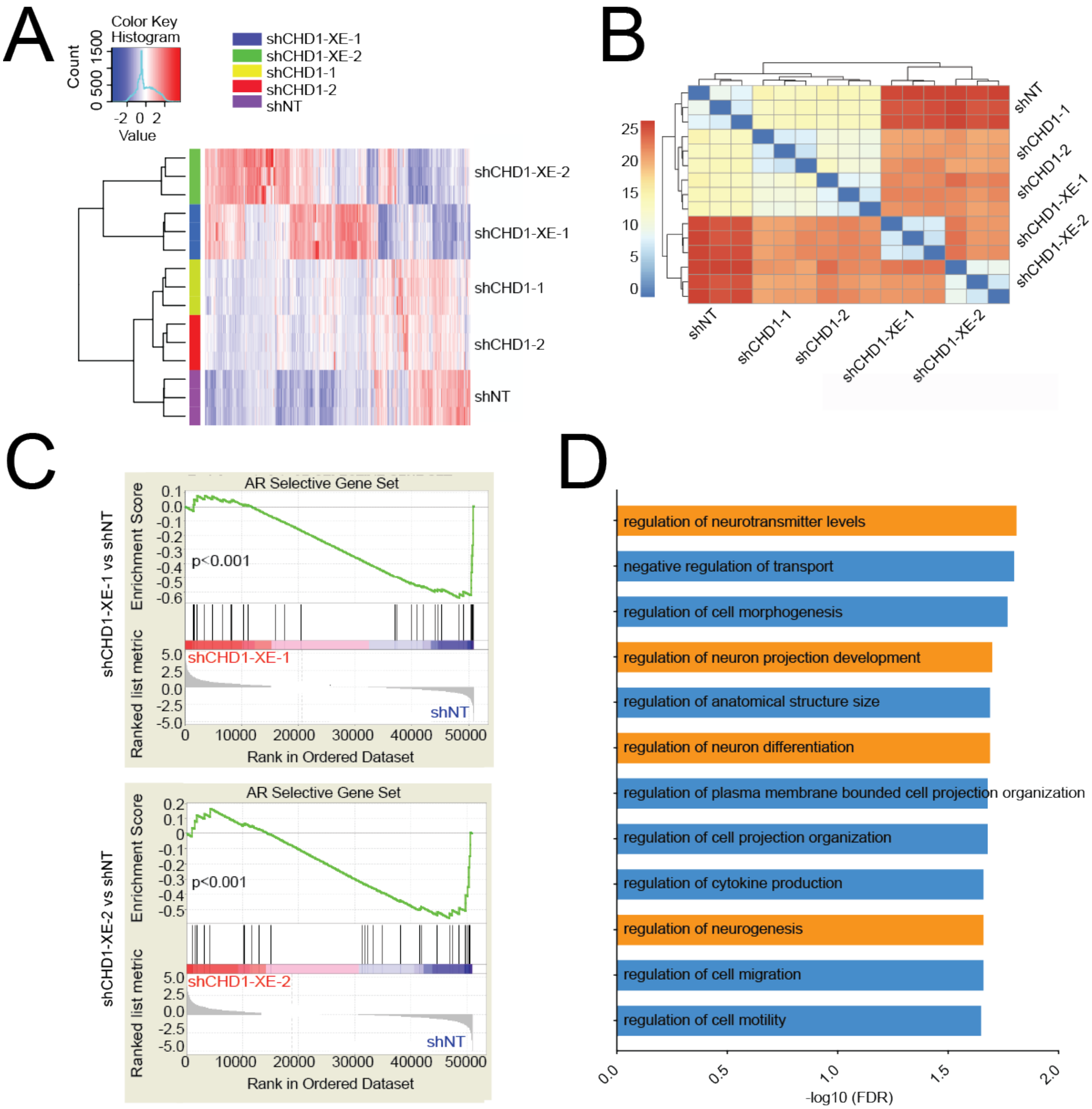

Figure S5 related to Figure 5. RNA-seq analysis reveals global changes in transcriptome profiling after *CHD1* loss. (A) Unsupervised hierarchical clustering of normalized expression of differentially expressed genes whose expression was significantly changed in any of the four other cell lines (shCHD1-1, shCHD1-2, shCHD1-XE-1, shCHD1-XE-2) comparing to shNT. 3 biological replicates in each group are shown. (B) Heatmap depicting the Euclidean distances between samples based on Pearson correlation. Reads from 3 biological replicates in each group were used for analysis. (C) GSEA analysis of AR selected genes (Arora et al. 2013) expression in shCHD1-XE groups compared to shNT group. Reads from 3 biological replicates were used for analysis. (D) Pathways enriched in the overlapped 150 significantly upregulated genes (see also Figure 5E) in the four cell groups compared to shNT. Reads from 3 biological replicates in each group were used for analysis.

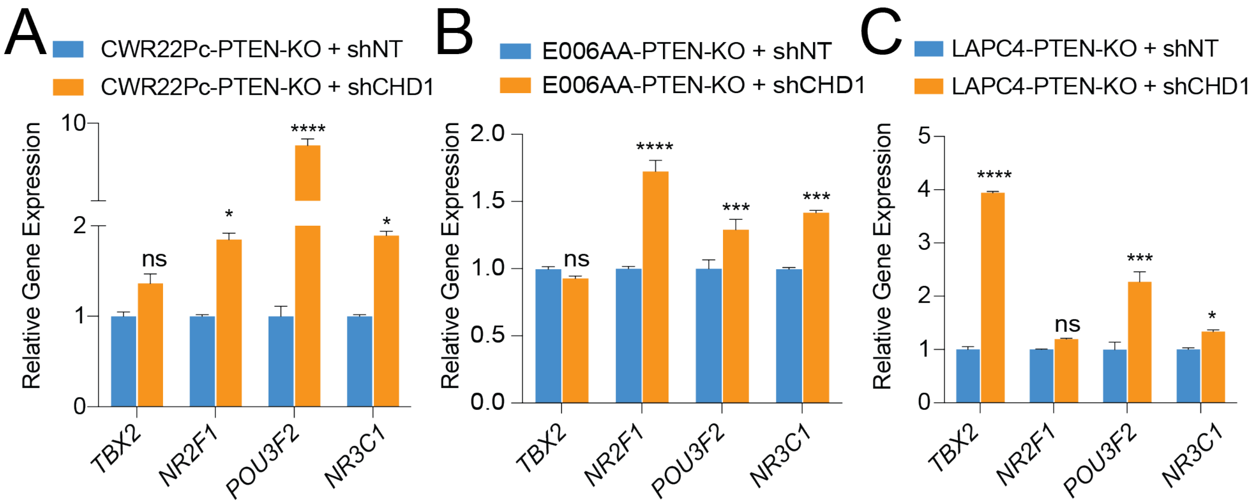

Figure S6 related to Figure 6. *CHD1* loss leads to induction of 4 TFs in other PCa models. (A) Relative gene expression of 4 TFs in CWR22Pc-PTEN-KO cells transduced with annotated shRNAs, all normalized and compared to shNT. (B) Relative gene expression of 4 TFs in E006AA-PTEN-KO cells transduced with annotated shRNAs, all normalized and compared to shNT. (C) Relative gene expression of 4 TFs in LAPC4-PTEN-KO cells transduced with annotated shRNAs, all normalized and compared to shNT. For all panels, mean ± SEM is represented and p values were calculated by multiple t test, 3 technical replicates in each group and **** p<0.0001. *** p<0.001. ** p<0.01. * p<0.05.

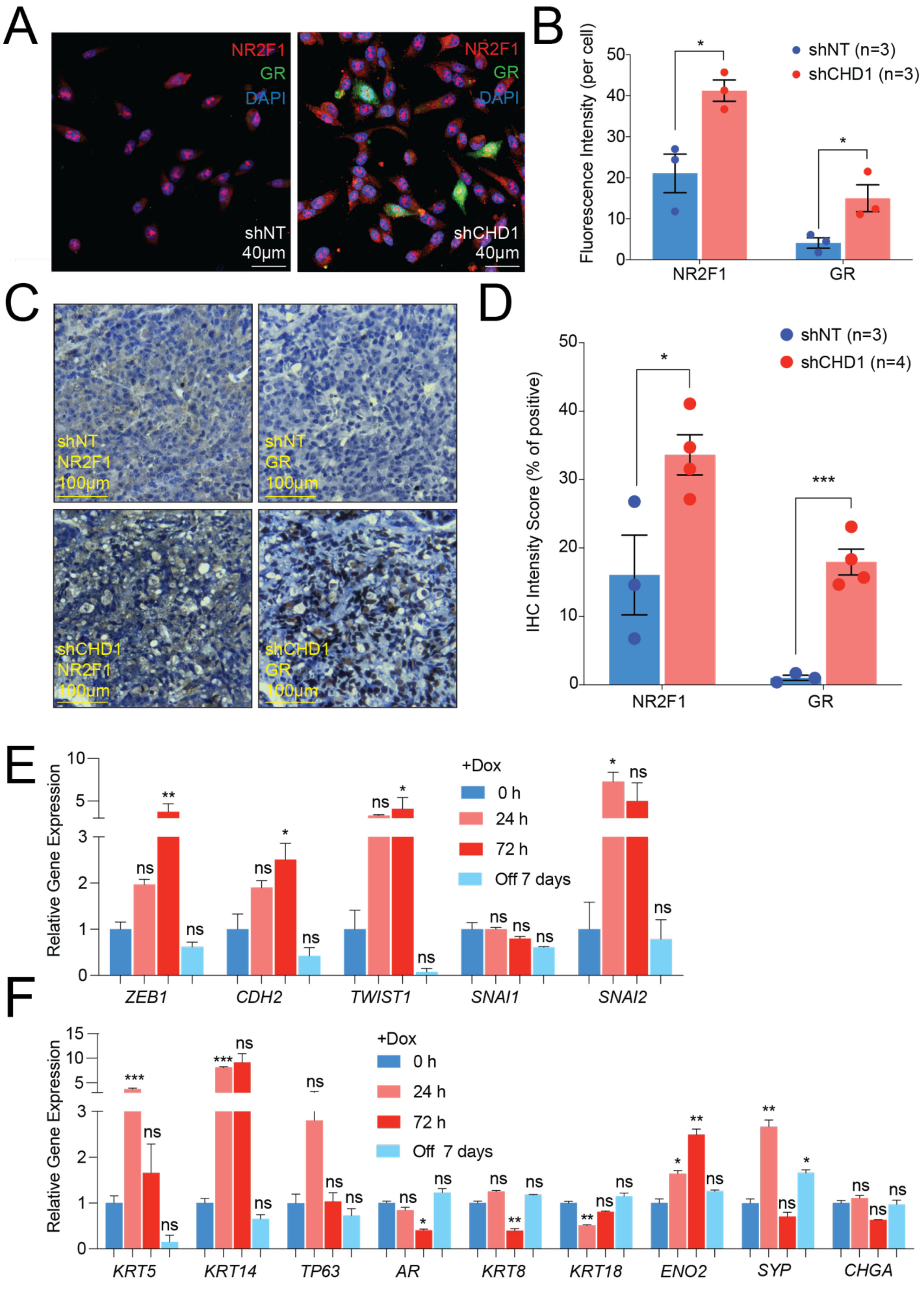

A
NR2F1
GR
DAPI
shNT
40μm
shCHD1
40μm
B
Fluorescence Intensity (per cell)
shNT (n=3)
shCHD1 (n=3)
NR2F1
GR
C
shNT
NR2F1
100μm
shNT
GR
100μm
shCHD1
NR2F1
100μm
shCHD1
GR
100μm
D
IHC Intensity Score (% of positive)
shNT (n=3)
shCHD1 (n=4)
NR2F1
GR
E
Relative Gene Expression
+Dox
0 h
24 h
72 h
Off 7 days
ZEB1
CDH2
TWIST1
SNAI1
SNAI2
F
Relative Gene Expression
+Dox
0 h
24 h
72 h
Off 7 days
KRT5
KRT14
TP63
AR
KRT8
KRT18
ENO2
SYP
CHGA

Figure S7 related to Figure 7. *CHD1* loss enhanced prostate cancer cell heterogeneity and lineage plasticity. (A) Immunofluorescence staining of NR2F1 and GR in shCHD1-XE cells. (B) Quantification of representative immunofluorescence images. Mean ± SEM is represented and p value was calculated by multiple t test. (C) Immunohistochemical staining of NR2F1 and GR on shCHD1 enzalutamide resistant tumor slides. (D) Quantification of representative immunohistochemical images. Mean ± SEM is represented and p value was calculated by multiple t test. (E-F) Relative gene expression level of the EMT genes (E) and lineage specific marker genes (F) in LNCaP/AR cells transduced with annotated inducible shRNAs at various time points. Mean ± SEM is represented and p values were calculated by two-way ANOVA, all compared to 0 hr, 3 technical replicates in each group. For all panels, **** $p<0.0001$. *** $p<0.001$. ** $p<0.01$. * $p<0.05$.

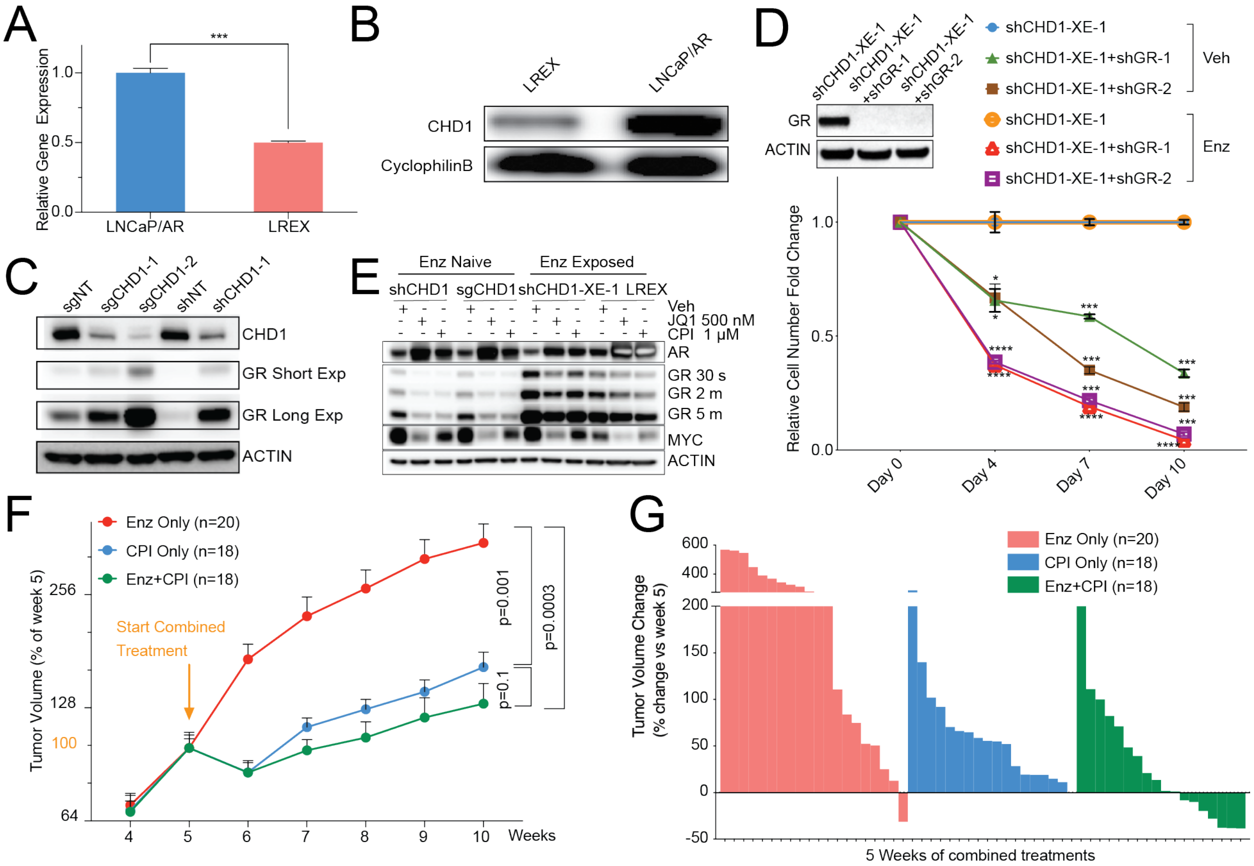

Figure S8 related to Figure 8. BET Bromodomain inhibition restores enzalutamide sensitivity. (A) Relative gene expression level of the *CHD1* in LNCaP/AR and LREX cells. Mean ± SEM is represented and p values were calculated by t test, 3 technical replicates in each group. (B) Western blot showing CHD1 protein levels in LNCaP/AR and LREX cells. (C) Western blot showing GR protein levels in LNCaP/AR cells transduced with annotated shRNAs or sgRNAs. (D) Relative cell number fold change compared to shCHD1-XE-1 group, based on the results of FACS-based competition assay. p values were calculated using two-way ANOVA. Mean ± SEM is represented and p values were calculated by two-way ANOVA, all compared to shCHD1-XE-1+Veh, 3 biological replicates in each group. Western blot showing GR protein levels in shCHD1-XE-1 cells transduced with annotated shRNAs. (E) Western blot of AR, GR, and MYC protein levels in different cell lines with enzalutamide resistance. (F) Tumor growth curve of xenografted LNCaP/AR shCHD1-XE-1 cells. All animals were treated with enzalutamide at 10 mg/kg orally 1 day after grafting. Beginning from week 5 of xenografting, animals were randomized into 3 groups and treated with enzalutamide only (Enz), CPI-0610 only (CPI) or the combination of enzalutamide plus CPI-0610. Mean ± SEM is represented and p values were calculated using two-way ANOVA. (G) Waterfall plot displaying changes in tumor size of xenografted LNCaP/AR shCHD1-XE-1 cells after 3 weeks of treatments. For (F) and (G), Enz denotes enzalutamide treatment at 10 mg/kg orally. CPI denotes CPI-0610 treatment at 60 mg/kg orally. For all panels, **** $p<0.0001$. *** $p<0.001$. ** $p<0.01$. * $p<0.05$.
