# Supplementary material for: Loss of CHD1 Promotes Heterogeneous Mechanisms of Resistance to AR-Targeted Therapy via Chromatin Dysregulation
Source: Cancer Cell. 2020 Apr 13;37(4):584–598.e11. doi: 10.1016/j.ccell.2020.03.001 (PMC7292228; doi:10.1016/j.ccell.2020.03.001)
Supplement: Document S2. Article plus Supplemental Information [file mmc8.pdf]

Cancer Cell

Article

Loss of *CHD1* Promotes Heterogeneous Mechanisms of Resistance to AR-Targeted Therapy via Chromatin Dysregulation

Graphical Abstract

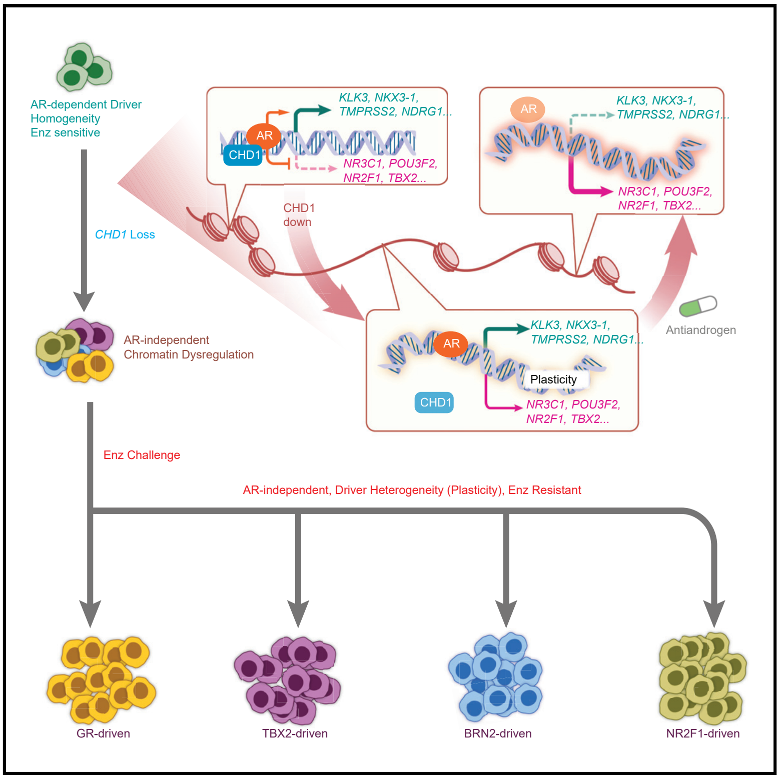

Highlights

- *In vivo* screen identifies drivers of antiandrogen resistance in prostate cancer
- *CHD1* loss establishes a state of chromatin dysregulation and lineage plasticity
- Heterogeneous mechanisms were enabled to escape from luminal lineage and therapy
- Patients with reduced tumor *CHD1* levels have a shorter response to antiandrogen

Authors

Zeda Zhang, Chuanli Zhou, Xiaoling Li, ..., Venkat S. Malladi, Charles L. Sawyers, Ping Mu

Correspondence

sawyersc@mskcc.org (C.L.S.),
ping.mu@utsouthwestern.edu (P.M.)

In Brief

Performing an *in vivo* shRNA screen, Zhang et al. identify that *CHD1* loss confers antiandrogen resistance. Low *CHD1* levels correlate with shorter clinical responses to enzalutamide. *CHD1* loss induces global chromatin changes, increasing expression of transcription factors that contribute to antiandrogen resistance.

Zhang et al., 2020, Cancer Cell *37*, 584–598
April 13, 2020 © 2021 The Authors. Published by Elsevier Inc.
https://doi.org/10.1016/j.ccell.2020.03.001

CellPress
Cancer Cell
Article

Loss of *CHD1* Promotes Heterogeneous Mechanisms of Resistance to AR-Targeted Therapy via Chromatin Dysregulation

Zeda Zhang,[1,3,19] Chuanli Zhou,[2,19] Xiaoling Li,[2,19] Spencer D. Barnes,[4] Su Deng,[2] Elizabeth Hoover,[1] Chi-Chao Chen,[5,6] Young Sun Lee,[1] Yanxiao Zhang,[7] Choushi Wang,[2] Lauren A. Metang,[2] Chao Wu,[1] Carla Rodriguez Tirado,[2] Nickolas A. Johnson,[2] John Wongvipat,[1] Kristina Navrazhina,[6] Zhen Cao,[1,6] Danielle Choi,[1] Chun-Hao Huang,[5,6] Eliot Linton,[1] Xiaoping Chen,[8] Yupu Liang,[9] Christopher E. Mason,[10,11,12] Elisa de Stanchina,[8] Wassim Abida,[13] Amaia Lujambio,[14] Sheng Li,[15] Scott W. Lowe,[5,16] Joshua T. Mendell,[2,16] Venkat S. Malladi,[4] Charles L. Sawyers,[1,16,*] and Ping Mu[2,17,18,20,*]
[1]Human Oncology and Pathogenesis Program, Memorial Sloan Kettering Cancer Center, New York, NY 10065, USA
[2]Department of Molecular Biology, UT Southwestern Medical Center, Dallas, TX 75390, USA
[3]Louis V. Gerstner, Jr. Graduate School of Biomedical Sciences, Memorial Sloan Kettering Cancer Center, New York, NY 10065, USA
[4]Bioinformatics Core Facility of the Lyda Hill Department of Bioinformatics, UT Southwestern Medical Center, Dallas, TX 75390, USA
[5]Cancer Biology and Genetics Program, Memorial Sloan Kettering Cancer Center, New York, NY 10065, USA
[6]Weill Cornell Graduate School of Medical Sciences, New York, NY 10021, USA
[7]Ludwig Institute for Cancer Research, La Jolla, CA, USA
[8]Department of Molecular Pharmacology, Memorial Sloan Kettering Cancer Center, New York, NY 10065, USA
[9]Center for Clinical and Translational Science, Rockefeller University, New York, NY 10065, USA
[10]Department of Physiology and Biophysics, Weill Cornell Medicine, New York, NY, USA
[11]The HRH Prince Alwaleed Bin Talal Bin Abdulaziz Alsaud Institute for Computational Biomedicine, Weill Cornell Medicine, New York, NY, USA
[12]The WorldQuant Initiative for Quantitative Prediction, Weill Cornell Medicine, New York, NY, USA
[13]Department of Medicine, Memorial Sloan Kettering Cancer Center, New York, NY 10065, USA
[14]Department of Oncological Sciences, Icahn School of Medicine at Mount Sinai, New York, NY 10029, USA
[15]The Jackson Laboratory for Genomic Medicine, Farmington, CT 06032, USA
[16]Howard Hughes Medical Institute, Chevy Chase, MD 20815, USA
[17]Hamon Center for Regenerative Science and Medicine, UT Southwestern Medical Center, Dallas, TX 75390, USA
[18]Harold C. Simmons Comprehensive Cancer Center, UT Southwestern Medical Center, Dallas, TX 75390, USA
[19]These authors contributed equally
[20]Lead Contact
*Correspondence: sawyersc@mskcc.org (C.L.S.), ping.mu@utsouthwestern.edu (P.M.)
https://doi.org/10.1016/j.ccell.2020.03.001

SUMMARY

Metastatic prostate cancer is characterized by recurrent genomic copy number alterations that are presumed to contribute to resistance to hormone therapy. We identified *CHD1* loss as a cause of antiandrogen resistance in an *in vivo* small hairpin RNA (shRNA) screen of 730 genes deleted in prostate cancer. ATAC-seq and RNA-seq analyses showed that *CHD1* loss resulted in global changes in open and closed chromatin with associated transcriptomic changes. Integrative analysis of this data, together with CRISPR-based functional screening, identified four transcription factors (NR3C1, POU3F2, NR2F1, and TBX2) that contribute to antiandrogen resistance, with associated activation of non-luminal lineage programs. Thus, *CHD1* loss results in chromatin dysregulation, thereby establishing a state of transcriptional plasticity that enables the emergence of antiandrogen resistance through heterogeneous mechanisms.

Significance

We describe a strategy to comprehensively identify genomic loss-of-function alterations in metastatic prostate cancer through an *in vivo* shRNA library screening approach. We find that loss of *CHD1*, a commonly deleted prostate cancer gene, confers resistance to the next-generation antiandrogen enzalutamide by establishing a state of chromatin dysregulation. This altered chromatin landscape facilitates the emergence of lineage plasticity by upregulation of transcription factors that promote differentiation away from the luminal lineage. Furthermore, we find that clinical response to enzalutamide is shorter in patients whose tumors have reduced *CHD1* levels.

 © 2021 The Authors. Published by Elsevier Inc.
This is an open access article under the CC BY license (http://creativecommons.org/licenses/by/4.0/).

INTRODUCTION

Targeted therapies for driver oncogenes have transformed the clinical management of many cancers but the magnitude and duration of response remains variable, even among patients with the same driver mutation and tumor histology. One potential explanation for this heterogeneity is the presence of additional genomic alterations that modify the degree of dependence on the targeted driver mutation. Metastatic prostate cancer serves as a relevant example, where the molecular target is the androgen receptor (AR) which functions as a lineage survival factor of luminal prostate epithelial cells. Next-generation AR therapies, such as abiraterone, enzalutamide, and apalutamide have significantly improved survival of men with castration-resistant prostate cancer, but resistance remains an issue (Beer et al., 2014; Ryan et al., 2013; Smith et al., 2018). Some patients fail to respond despite robust AR expression, whereas others relapse quickly.

Mechanisms of resistance to AR therapy fall into three general categories: (1) restoration of AR signaling; (2) bypass of AR signaling via other transcription factors (TFs), e.g., glucocorticoid receptor (Arora et al., 2013; Isikbay et al., 2014); and (3) AR-independent signaling (reviewed in Watson et al., 2015). One example of the latter category is combined loss of function of the *TP53* and *RB1* tumor suppressors, which confers resistance by promoting lineage transition to a state that is no longer dependent on AR and its downstream signaling pathway (Ku et al., 2017; Mu et al., 2017). Similar cases of lineage plasticity in the context of drug resistance have been documented in epidermal growth factor receptor-mutant lung adenocarcinoma and in BRAF-mutant melanoma, including transition to neuroendocrine or mesenchymal phenotypes (Garraway et al., 2005; Park et al., 2018; Sequist et al., 2011). These examples provide clear precedent for how co-occurring genomic alterations can affect response to targeted therapies. Due to the heterogeneous number of copy number alterations (Abida et al., 2017; Barbieri et al., 2012; Beltran et al., 2011, 2016; Grasso et al., 2012; Holcomb et al., 2009; Kim et al., 2007; Robinson et al., 2015; Taylor et al., 2010), we surveyed the genomic landscape of metastatic castration-resistant prostate cancer (mCRPC) for modifiers of sensitivity to AR therapy.

RESULTS

Enrichment of shRNAs Targeting *CHD1* in an *In Vivo* Enzalutamide Resistance Screen

To identify genomic modifiers of sensitivity to AR therapy, we constructed a pooled small hairpin RNA (shRNA) library targeting genes most frequently deleted in primary or metastatic prostate cancer, then screened for resistance to enzalutamide in a well-credentialed enzalutamide-sensitive xenograft model (Arora et al., 2013; Tran et al., 2009). The decision to conduct the screen *in vivo* was based on the fact that *in vivo* models provide a more physiologic context for studying castration-resistant growth than *in vitro* models, which rely on the use of charcoal-stripped serum to emulate castrate level of androgens. Indeed, in our hands findings from *in vivo* screens have often been confirmed in clinical datasets (Arora et al., 2013; Balbas et al., 2013).

We generated a list of 730 genes deleted in human prostate cancer (Table S1) through bioinformatic mining of 6 independent genomic datasets as described in the STAR Methods (Barbieri et al., 2012; Grasso et al., 2012; Holcomb et al., 2009; Kim et al., 2007; Network, 2015; Taylor et al., 2010). We then constructed an shRNA library targeting these genes (5–6 hairpins per gene × 730 genes = 4,234 hairpins total) using the miR-E-derived system, which has significantly improved knockdown efficiency and target specificity compared with traditional shRNA approaches (Fellmann et al., 2013) (Figure 1A; Table S2). We conducted our screen *in vivo*, using the enzalutamide-sensitive LNCaP/AR xenograft model, with the goal of identifying shRNAs enriched during enzalutamide therapy (Figure 1B). One challenge of *in vivo* screens is assurance of adequate library representation, since not all cells injected *in vivo* will contribute to the established tumors. This can be managed by limiting the number of shRNAs per injection and by dividing the library into distinct pools (Zuber et al., 2010, 2011). In a pilot experiment using the enzalutamide-resistant AR mutant (F877L) as a positive control (Balbas et al., 2013), we found that dilution of one F877L-positive cell in 100 parental LNCaP/AR cells consistently gave rise to tumors in enzalutamide-treated mice after ~6 weeks, compared with ~19 weeks for cells infected with the non-targeting control vector (shNT) (Figure S1A). Based on this result, we concluded that a pool size of 100 shRNAs should give adequate representation and therefore subdivided the library into 43 pools with ~100 shRNAs per pool. Enzalutamide functions as an agonist on the F877L mutant and may be more potent in this assay than the shRNAs to be screened; therefore, we selected 16 weeks as an optimal time to harvest tumors, before the appearance of background tumors. Each pool was screened using 10 independent injections to ensure that we could identify those shRNAs that were reproducibly enriched and eliminate those that were enriched due to stochastic clonal expansion (bystander shRNAs).

Multiple tumors emerged by 16 weeks from 40 of 43 pools screened (Figure S1B). Genomic DNA was extracted from these tumors and analyzed by next-generation sequencing to determine the enrichment of specific shRNAs compared with the starting material (Figure 1B). As expected, the abundance of most hairpins was reduced due to dilution by more rapidly expanding clones. This is apparent from comparing the normalized read counts of the starting plasmid library and pregraft populations (tightly distributed) to the tumors (broad distribution) (Figures 1C and 1D). Then we utilized a classic algorithm RIGER-E (RNAi Gene Enrichment Ranking) to rank the 730 genes based on the normalized read counts of all hairpins in both starting plasmid library/pregraft and resistant tumor populations, as described in the STAR Methods section. A p value of <0.0001 resulted in 172 genes as potential candidates (Figure 2A; Table S3). *TBC1D4* serves as a useful negative control because this gene is already deleted in an LNCaP/AR model and is ranked near the bottom, as expected (Taylor et al., 2010). Considering the potential for stochastic enrichment of biologically inert hairpins *in vivo*, we applied 2 additional filters to this list of 172 genes to enhance the probability of selecting true positives (1) enrichment in >8% of total tumors xenografted (cutoff selected based on stochastic enrichment rate of 8% for the negative control gene *TBC1D4*) (Figure 2B) and (2) enrichment of >4 independent hairpins targeting a specific gene (to avoid off-target effects) (Figure 2C). Application of these filters yielded eight candidate

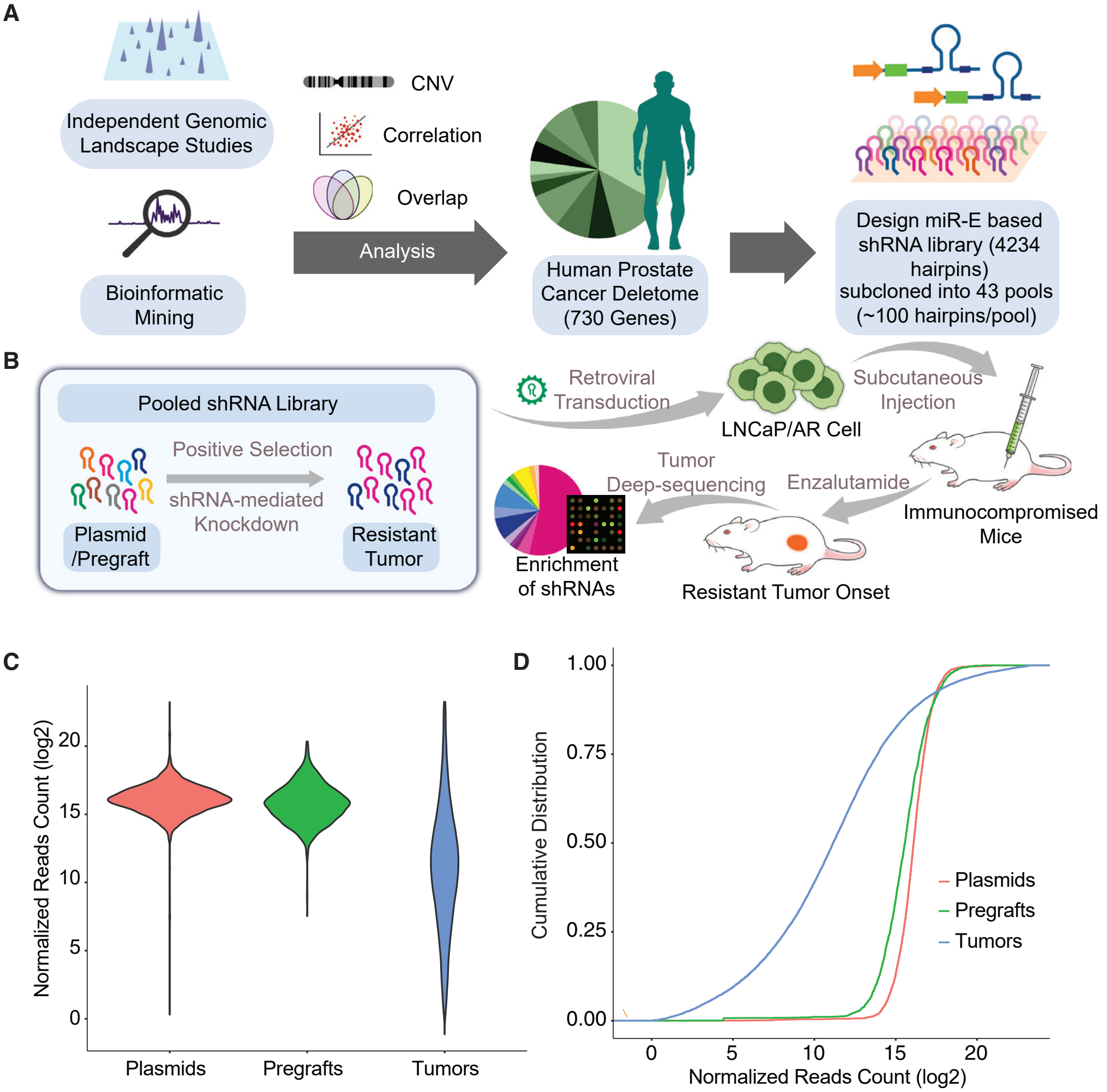

Figure 1. An *In Vivo* shRNA Library Screen of the Human Prostate Cancer Deletome

(A) Schematic representation of a miR-E shRNA library targeting the human prostate cancer deletome.

(B) Schematic representation of enzalutamide resistance screen using the miR-E shRNA library.

(C) Violin plot of the shRNA normalized read counts in the combined plasmid pools (n = 43), pregrafts (n = 21), and enzalutamide-resistant tumors (n = 344).

(D) Cumulative distribution of library shRNAs in the combined plasmid pools (n = 43), pregrafts (n = 21), and enzalutamide-resistant tumors (n = 344).

See also Figure S1 and Tables S1 and S2.

genes (Figure 2A; Table S3). Chromodomain helicase DNA-binding protein 1 (*CHD1*) was selected for further analysis based on its high frequency of deletion in prostate cancer (Augello et al., 2019; Grasso et al., 2012; Ren et al., 2018; Robinson et al., 2015; Rodrigues et al., 2015; Shenoy et al., 2017; Zhao et al., 2017). A representative example of *CHD1* shRNA enrichment from one of the pools is shown in Figure 2D.

CHD1 Loss Confers Enzalutamide Resistance *In Vitro* and *In Vivo*

In normal tissues, *CHD1* functions as a chromatin remodeler and is required to maintain the open chromatin state of pluripotent embryonic stem cells and for somatic cell reprogramming (Gaspar-Maia et al., 2009). Numerous lines of evidence from cell lines and genetically engineered mice implicate *CHD1* as a tumor suppressor, including in primary prostate cancer (Augello et al., 2019; Huang et al., 2011; Liu et al., 2012; Rodrigues et al., 2015; Shenoy et al., 2017; Zhao et al., 2017). To determine the link between *CHD1* loss and enzalutamide resistance, we performed validation experiments using five different stable shRNAs and two different CRISPR guides. *CHD1*-depleted cells consistently grew faster in enzalutamide-containing medium than *CHD1* wild-type cells, as measured in proliferation assays, dose-response assays and a fluorescence-activated cell sorting-based competition assay (Figures 3A–3D and S2A–S2D). Similar results were observed with two other next-

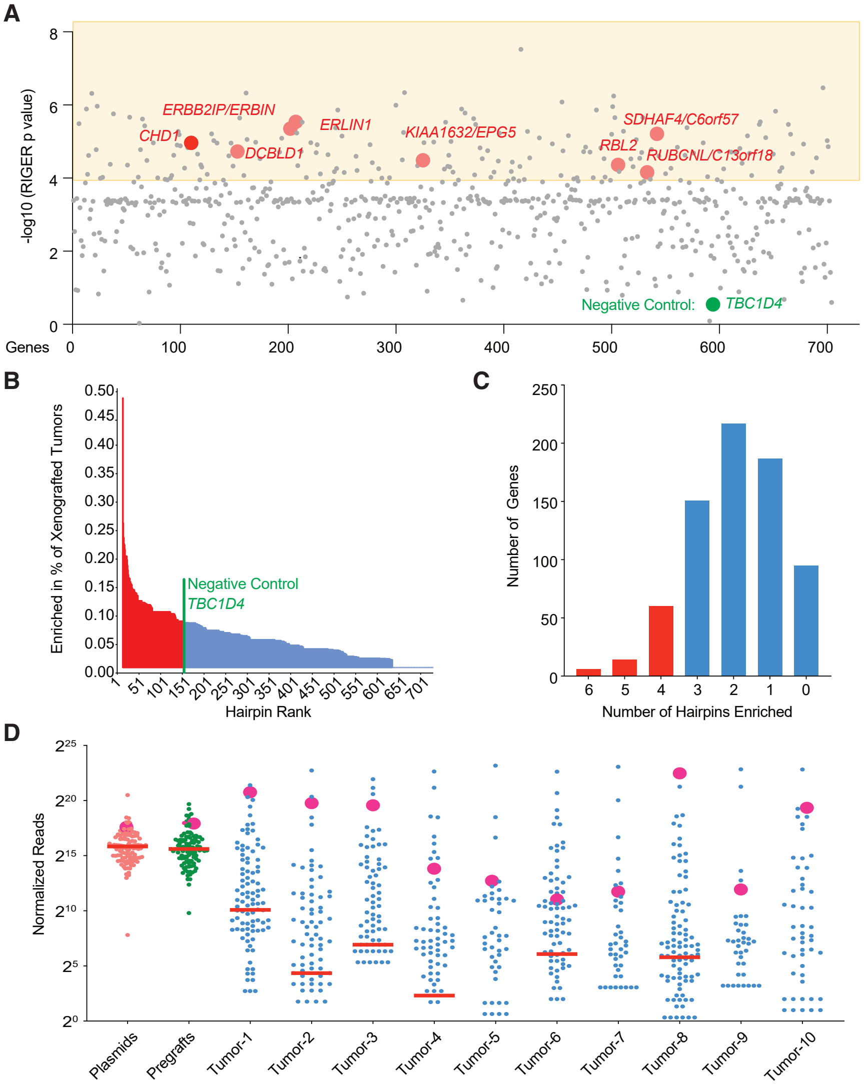

Figure 2. *In Vivo* Screen Identifies *CHD1* as Top Candidate Responsible for Resistance to Antiandrogen

(A) Graphical representation of analyzed results of the library screen, using RIGER-E method. –Log10 of p value is presented and the area of $p < 0.0001$ is highlighted. The top eight candidate genes are presented as large red dots with gene symbol. Negative control gene *TBC1D4* is presented as a large green dot.

(B) Graphical representation of the percentage of tumors which have shRNAs targeting a specific gene and are enriched in resistant tumors.

(C) Graphical representation of the number of genes which have multiple independent shRNAs enriched in resistant tumors.

(D) Bee swarm plot of the normalized shRNA read counts of a representative pool in the plasmid, pregraft, and resistant tumors, median is presented as a red line (medians below 1 are not presented on log2 scale). shCHD1s are presented as large red dots.

See also Table S3.

generation AR inhibitors, apalutamide and darolutamide (Figure S2D). These findings were confirmed *in vivo* in castrated mice treated with enzalutamide (Figures 3E and S2E). In addition to *CHD1*, we confirmed that knockdown of two other candidate genes (*RUBCNL* and *RBL2*) also confers enzalutamide resistance in LNCaP/AR cells cultured *in vitro* (Figure S2F). Analysis of the other five candidates will be reported separately.

Importantly, enzalutamide resistance conferred by *CHD1* knockdown was fully rescued by introducing the full-length *CHD1* cDNA (Figure S2C). Using a doxycycline-inducible shRNA knockdown model, we also confirmed that enzalutamide resistance conferred by *CHD1* knockdown is rapid and reversible (Figures S2G–S2I). *CHD1* knockdown also conferred *in vitro* resistance to enzalutamide in the human prostate cancer cell lines CWR22Pc, LAPC4, and E006AA (but only in the context of *PTEN* loss) and in a genetically defined mouse organoid model (*Pten*$^{-/-}$) cultured in 3D, as well as *in vivo* resistance in the CWR22Pc xenograft model (Figures S3A–S3G).

Low *CHD1* mRNA Level Is Associated with Shorter Treatment Response in CRPC Patients

A recent mCRPC genomic landscape study with linked longitudinal clinical outcome data provided an opportunity to address whether *CHD1* loss in patients is associated with poor clinical response to next-generation antiandrogen therapy (Abida et al., 2019). Within this landscape study we identified 56 CRPC patients treated with either abiraterone or enzalutamide on whom tumor whole-exome and RNA sequencing (RNA-seq) data were available within 30 days before treatment. We initially asked if genomic *CHD1* loss was associated with treatment response but there were too few cases to run the analysis (only two with homozygous *CHD1* deletion). We therefore asked if *CHD1* mRNA expression is correlated with outcome. A Cox proportional hazards regression model was fitted on log2 (*CHD1* mRNA level) as a continuous predictor, which showed a regression coefficient of −0.39 and p value of 0.11. Although this analysis did not meet the threshold for statistical significance, it raised the possibility that lower *CHD1* mRNA levels may have higher relative hazards or, in other words, confer a higher risk to the patients. Indeed, a Pearson correlation analysis showed that *CHD1* mRNA level is significantly correlated with progression-free survival time ($p = 0.021$) (Figure 4A). To further dissect this correlation, we divided the cohort into quartiles based on the *CHD1* mRNA levels, which revealed a Gaussian-like distribution (Figure 4B). We excluded 4 of the 56 patients who had *SPOP* mutations (who were distributed evenly across the quartiles) because these patients have increased sensitivity to abiraterone (Boysen et al., 2018). Patients in the lowest quartile of tumor *CHD1* expression had a significantly shorter time to progression on either enzalutamide

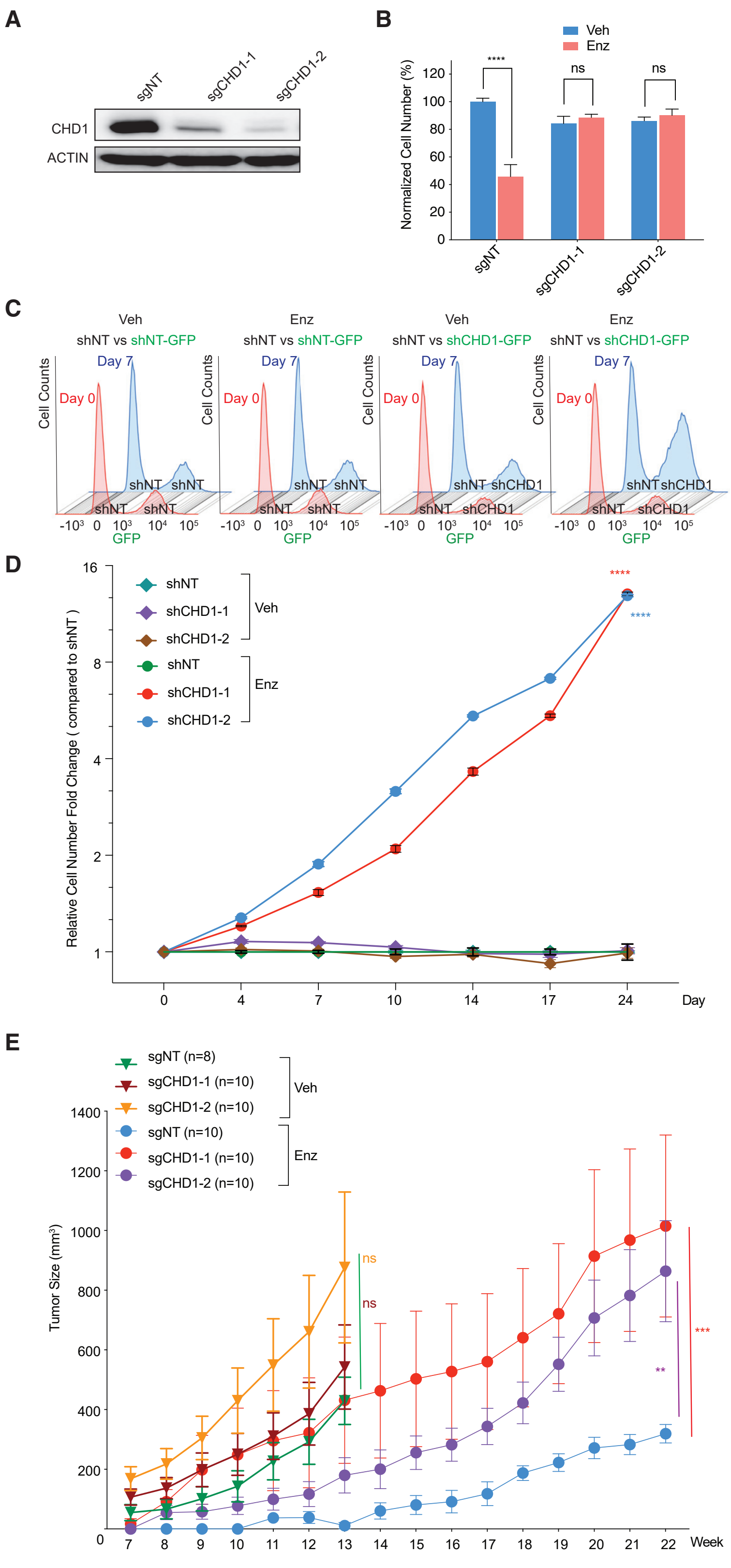

Figure 3. *CHD1* Loss Confers Significant Resistance to Antiandrogen *In Vitro* and *In Vivo*

(A) Western blot of CHD1 in LNCaP/AR cells transduced with annotated guide RNAs.

(B) Relative cell number of LNCaP/AR cells transduced with annotated guide RNAs, normalized to sgNT + Veh group. Cells were treated with 10 μM enzalutamide (Enz) or DMSO (Veh) for 7 days and cell numbers were counted. p values were calculated using multiple t tests, three biological replicates in each group.

(C) Histograms of representative fluorescence-activated cell sorting-based competition assay showing the distribution of shNT LNCaP/AR cells (GFP-negative) compared with cells transduced with *cis*-linked shCHD1-GFP or shNT-GFP shRNAs (GFP positive). The distribution on day 0 is shown in red and day 7 is shown in blue.

(D) Relative cell number fold change compared with shNT group, based on the results of (C). Enz denotes enzalutamide of 10 μM and Veh denotes DMSO. p values were calculated using two-way ANOVA, three biological replicates in each group.

(E) Tumor growth curve of xenografted LNCaP/AR cells transduced with annotated guide RNAs. Enz denotes enzalutamide treatment at 10 mg/kg from day 1 of grafting. Veh denotes 0.5% CMC + 0.1% Tween 80.

p values were calculated using two-way ANOVA. For all panels, mean ± SEM is presented. ****$p < 0.0001$, ***$p < 0.001$, **$p < 0.01$, *$p < 0.05$. See also Figures S2 and S3.

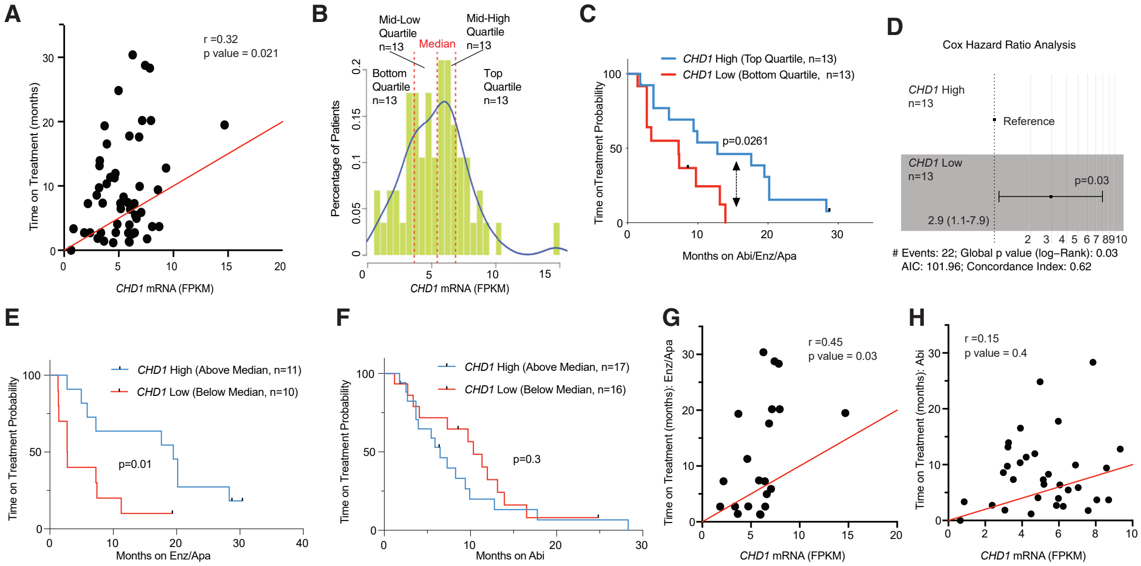

Figure 4. *CHD1* mRNA Level Is Correlated with Clinical Outcome of Antiandrogen Treatment

(A) Pearson correlation analysis of *CHD1* mRNA and time of treatment on abiraterone (Abi)/enzalutamide (Enz)/apalutamide (Apa) of a 52 mCRPC patient cohort.

(B) *CHD1* expression distribution in all patients of the cohort in (A).

(C) Probability of treatment duration of the top quartile compared with bottom quartile of all patients treated with abiraterone (Abi)/enzalutamide (Enz)/apalutamide (Apa); p value was calculated using Mantel-Cox test.

(D) Cox hazard ratio analysis of the top and bottom quartile of all patients, p value was calculated using log rank test.

(E) Probability of treatment duration of the above median compared with below median of patients who received enzalutamide (Enz)/apalutamide (Apa), p value was calculated using Mantel-Cox test.

(F) Probability of treatment duration of the above median compared with below median of patients who received abiraterone (Abi), p value was calculated using Mantel-Cox test.

(G) Pearson correlation analysis of *CHD1* mRNA and time of treatment on patients who received enzalutamide (Enz)/apalutamide (Apa), n = 21 (2 patients received both apalutamide and abiraterone).

(H) Pearson correlation analysis of *CHD1* mRNA and time of treatment on patients who received abiraterone (Abi), n = 33.

or abiraterone compared with the patients in the highest quartile (p = 0.0261) (Figure 4C), supporting the predictions from the preclinical findings. This finding is further supported by Cox hazards ratio analysis showing significant increased hazards related to low *CHD1* mRNA levels (Figure 4D). Interestingly, we find that the poor clinical outcome seen in patients with low *CHD1* expression is primarily seen in those treated with enzalutamide/apalutamide (Figure 4E) but not abiraterone (Figure 4F), which is confirmed by Pearson correlation analysis (Figures 4G and 4H). This distinction is consistent with our experimental data showing that *CHD1* deletion confers resistance to enzalutamide but not to androgen withdrawal in the LNCaP/AR model (as seen in charcoal-stripped serum treated with vehicle; Figures 3B, 3E, S2C, and S2D), raising the intriguing possibility of mechanistic differences in resistance to AR antagonists versus androgen-lowering agents.

Integrated Analysis of RNA-Seq and ATAC-Seq Reveals Candidate TF Drivers of Enzalutamide Resistance

To investigate the mechanism by which *CHD1* loss promotes antiandrogen resistance, we first asked if AR signaling activity was restored in these enzalutamide-resistant tumors. To our surprise, we observed sustained inhibition of the AR target genes *KLK3*, *NKX3-1*, *TMPRSS2*, *NDRG1*, *PMEPA1*, and *STEAP1*, indicating that canonical AR signaling is not restored (Figures 5A and 5B). This suggested that *CHD1* loss might activate transcriptional programs that relieve prostate tumor cells from their dependence on AR by reprogramming away from their luminal lineage, as we have reported previously in the setting of combined loss of *RB1* and *TP53* (Ku et al., 2017; Mu et al., 2017).

Because CHD1 plays a role in chromatin remodeling, we postulated that such lineage transitions (and their underlying transcriptional programs) could be identified by integrative analysis of global transcriptional and chromatin landscape changes induced by *CHD1* loss, as measured by RNA-seq and assay for transposase-accessible chromatin sequencing (ATAC-seq). To distinguish between transcriptional changes induced by *CHD1* loss alone versus enzalutamide treatment, we profiled LNCaP/AR cells that were not exposed to enzalutamide after stable *CHD1* knockdown (shCHD1-1 and shCHD1-2; two different shRNAs) as well as enzalutamide-resistant sublines of shCHD1-1 and shCHD1-2 derived after passage as xenografts in enzalutamide-treated mice (shCHD1-XE-1 and shCHD1-XE-2). ATAC-seq revealed substantial changes in open and closed chromatin after *CHD1* loss, consistent with the function of CHD1 in chromatin remodeling. Globally, we observed more than 10,000 new open and closed peaks, mainly in the intronic and intergenic regions (Figures 5C and 5D). *CHD1* loss also led to global changes in transcriptome profiling (Figure 5E) which were associated with changes in open chromatin (Figures S4A–S4D). The transcriptome changes were relatively similar in both shCHD1 sublines but were quite divergent in the

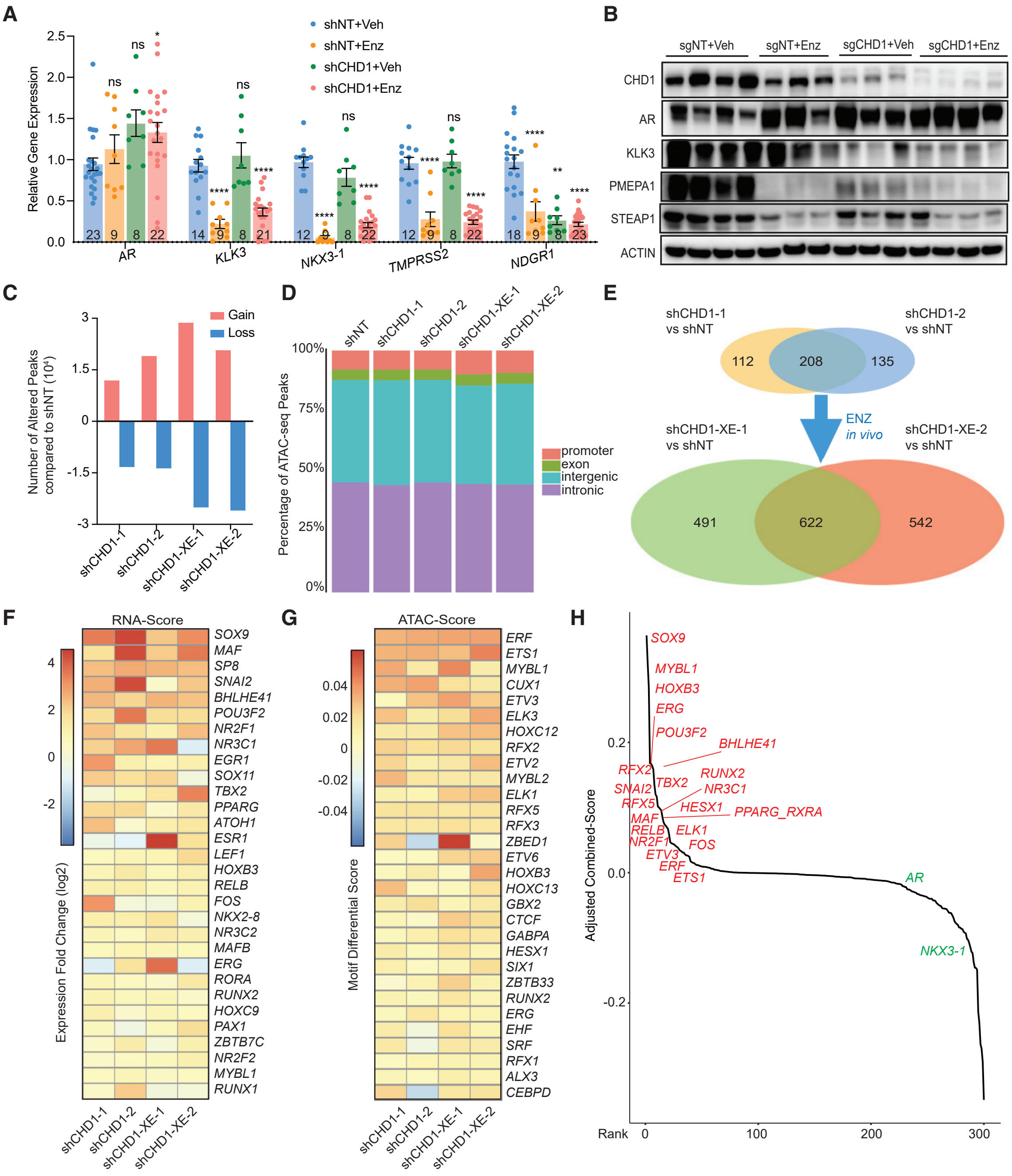

Figure 5. Integrated Analysis of RNA-seq and ATAC-Seq Reveals Candidate Transcription Factor Drivers of Enzalutamide Resistance

(A) Relative gene expression of AR and AR target genes in tumors collected from LNCaP/AR xenografts, all normalized and compared with shNT + Veh group. Mean ± SEM is presented. p values were calculated using two-way ANOVA and numbers of biological replicates are presented. ****$p < 0.0001$, ***$p < 0.001$, **$p < 0.01$, *$p < 0.05$.

(B) Western blot showing AR and AR targets in tumors collected from LNCaP/AR xenografts. For both (A) and (B), Enz denotes enzalutamide treatment at 10 mg/kg from day 1 of grafting. Veh denotes 0.5% CMC + 0.1% Tween 80.

(C) Graphical representation of the ATAC-seq peaks changes (gain or loss) in cell lines compared with shNT.

(D) The distribution of ATAC-seq peak locations in different genetic regions. For both (C) and (D), reads from three biological replicates were pooled to calculate the consensus peaks.

(E) Venn diagram represents the overlap of the most differentially expressed genes in four groups compared with shNT. Cutoff values of fold change greater than 2 and false discovery rate ≤ 0. 1 were used. Reads from three biological replicates in each group were used for analysis.

(F) Heatmap represents the expression fold changes (comparing to shNT) of the top 30 genes ranked by RNA-Score, three biological replicates in each group.

(legend continued on next page)

shCHD1-XE-1 and shCHD1-XE-2 sublines (Figures S5A and 5B), suggesting that enzalutamide exerts selective pressure that can result in distinct transcriptional outcomes. Interestingly, gene set enrichment analysis and pathway analysis revealed significant downregulation of AR-selective signature genes and enrichment of several neuron differentiation related pathways in shCHD1-XE tumor cell lines (Figures S5C and S5D; Table S4).

Because activation of downstream target genes is dependent on both the abundance of a TF as well as the accessibility of its cognate binding sites within chromatin, we integrated changes in TF expression with the presence of their associated binding motifs in areas of open chromatin. We first calculated an overall RNA-Score of TFs using the sum of weighted log fold change to identify those with significant changes in RNA level across all four *CHD1* loss conditions (Figure 5F). We then used motif analysis within the open peaks identified by ATAC-seq to calculate an overall ATAC-Score by summing the weighted motif differential scores derived from the DAStk tool (Figure 5G). Twenty-two TFs emerged after integration of upregulated TFs with the enriched motifs of each TF (by multiplying the overall RNA-Score and ATAC-Score), which we then evaluated as candidate drivers of enzalutamide resistance in context of *CHD1* loss (Figure 5H and Table S5).

Functional Screen Implicates Four TFs in Mediating Enzalutamide Resistance

To explore the functional role of these 22 TFs in antiandrogen resistance, we asked if CRISPR deletion of each TF alone would restore enzalutamide sensitivity in LNCaP/AR cells with *CHD1* knockdown. Four independent guide RNAs for each of the 22 genes were individually cloned into a viral vector with a *cis*-linked RFP gene, pooled and introduced into shCHD1 cells in a manner that resulted in a mixture of RFP-positive (range $\sim$50%–90%) and RFP-negative cells. For cells expressing guides targeting TFs required for enzalutamide resistance, we reasoned that the percentage of RFP-positive cells would decline over 7 days when cultured with enzalutamide (Figure 6A). In control cells infected with a non-targeting guide (sgNT) and in cells expressing guides targeting 18 of the 22 TFs, the fraction of RFP-positive cells did not change significantly (Figure 6B). However, RFP-positive cells were significantly depleted in cells expressing guides selectively targeting genes encoding each of four TFs: *NR3C1* (encoding GR), *POU3F2* (encoding BRN2), *TBX2*, and *NR2F1* (Figure 6B). Independent experiments confirmed that CRISPR deletion of each of these four TFs re-sensitized shCHD1 cells to enzalutamide *in vitro* (Figure 6C). Furthermore, their upregulation in the context of *CHD1* loss was reversible, as revealed by doxycycline-regulated *CHD1* shRNA knockdown (Figure 6D) and was evident in three other AR-positive human prostate cancer cell lines (Figures S6A–S6C).

Interestingly, all four TFs have been previously implicated in resistance to hormone therapy and prostate cancer progression, often in the context of aberrant lineage specification away from canonical luminal adenocarcinoma (Arora et al., 2013; Bishop et al., 2017; Du et al., 2017; Ku et al., 2017; Mu et al., 2017; Nandana et al., 2017; Shi et al., 2019; Sosa et al., 2015). To further interrogate their roles, we extended our analysis to a panel of $\sim$20 enzalutamide-resistant xenografts, each derived independently from LNCaP/AR after *CHD1* depletion by either shRNA or CRISPR deletion. Each of the four TFs had elevated expression in some but not all xenografts across this panel, supporting a heterogeneous profile across this isogenic series of sublines (Figure 7A). *NR3C1* was most frequently and substantially upregulated, but multiple sublines also had upregulation of *NR2F1*, *TBX2*, or *POU3F2*, sometimes without concurrent *NR3C1* upregulation. Immunofluorescence and immunohistochemical staining revealed heterogeneity within the shCHD1-XE-1 cell lines and in shCHD1 tumors resistant to enzalutamide, as seen by increased levels of NR2F1 in some cells and both NR2F1 and GR in other cells (Figures S7A–S7D). Collectively, this pattern and the results from an inducible shCHD1 model suggest a state of chromatin plasticity and enhanced heterogeneity, initiated by *CHD1* loss, which enables upregulation of distinct sets of genes in response to selective pressure.

This concept is further supported by *in vitro* studies where we examined the effect of brief exposure to enzalutamide on expression of each of the four TFs in *CHD1* wild-type cells or in those with *CHD1* depletion (by shRNA or CRISPR) (Figure 7B). Either *CHD1* loss or enzalutamide exposure was sufficient to modestly upregulate each of the four TFs, but transcriptional changes were more substantial under both conditions, particularly in the *CHD1*-deleted, enzalutamide-resistant xenograft-derived cell lines (Figures 7A and 7B). This hypothesis is also supported by RNA-seq data from the previously mentioned cohort of mCRPC patients (Abida et al., 2019), in which we examined the co-association of *CHD1* levels with each of these four TFs across 212 tumors. Unsupervised clustering analysis of just these five genes identified five distinct clusters (Figure 7C). Cluster 5 (*CHD1* high) is noteworthy because the relative expression of each of the four TFs is low; whereas clusters 2, 3, and 4 (*CHD1* low) each displays relatively higher expression of *NR2F1* and *POU3F2* (cluster 3), *TBX2* (cluster 2), or *NR3C1* (cluster 4). Cluster 1 (also *CHD1* low) is an outlier to this pattern because all four TFs are also low, which could be an indication of even greater heterogeneity beyond that elicited from the LNCaP/AR model. The identity of additional plasticity drivers could emerge through characterization of transcriptional and chromatin landscape changes across other models (Alizadeh et al., 2015).

An underlying assumption of our chromatin plasticity model is that the observed changes in TF activity promote enzalutamide resistance through loss of luminal lineage identity. Indeed, we observed altered expression of many canonical lineage-specific genes in the same panel of *CHD1*-deleted, enzalutamide-resistant xenografts that displayed heterogeneous upregulation of the four TFs (Figure 7D). For example, all tumors showed consistent downregulation of luminal marker genes (*AR*, *KRT8*, and *KRT18*), some had increased levels of basal marker genes

(G) Heatmap represents the motif differential changes (compared with shNT) of the top 30 genes ranked by ATAC-Score, three biological replicates in each group.
(H) Rank of candidate transcription factors (TFs) are shown based on the adjusted Combined-Score. Top candidate TFs selected for functional CRISPR library screen are presented in red.
See also Figures S4 and S5 and Table S4.

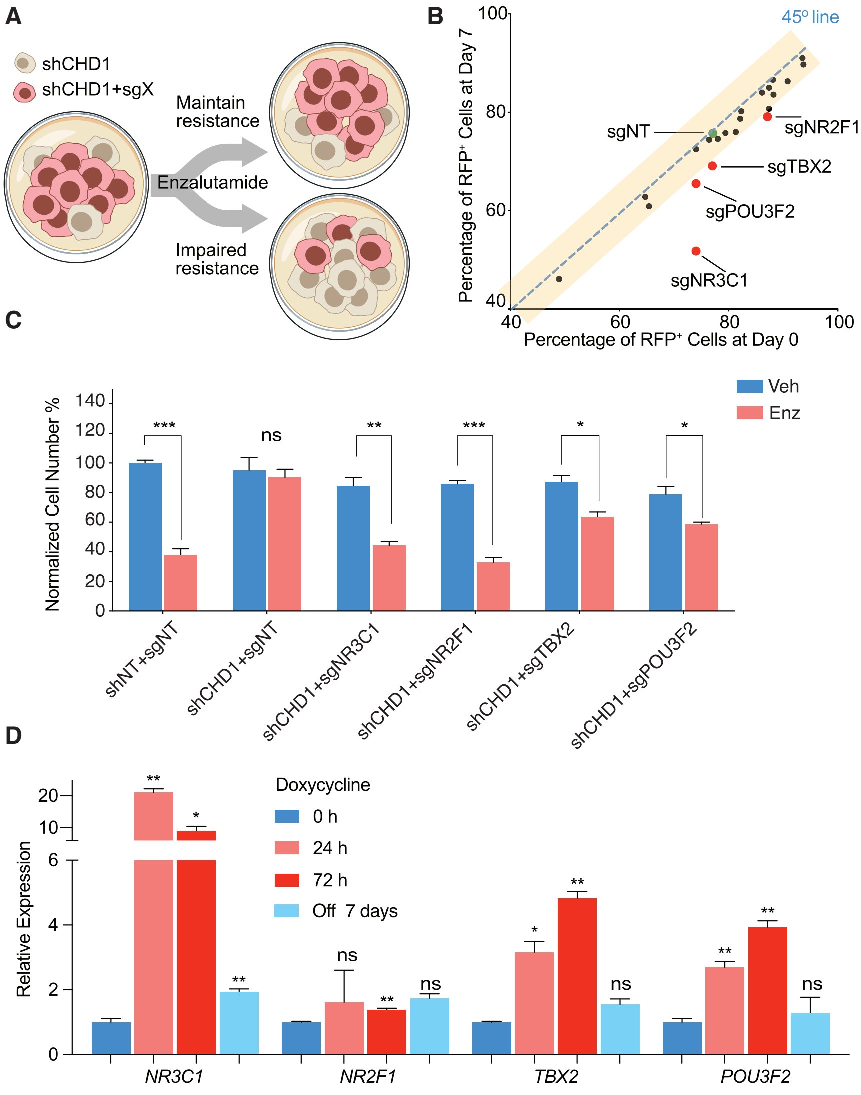

Figure 6. Functional CRISPR Screen Identifies Four Alternative TFs as Drivers of Antiandrogen Resistance

(A) Schematic representation of the functional CRISPR library screen in shCHD1 LNCaP/AR cells. shCHD1 cells were transduced with Cas9 and pooled single guide RNAs targeting individual TFs and achieved cell mixtures of 50%–90% RFP-positive cells (shCHD1 + sgTF) versus RFP-negative cells (shCHD1 only).

(B) Scatterplot summarizing the results of the screen. Each dot represents pooled guide RNAs targeting a specific gene. The x axis is the percentage of RFP cells at day 0 and the y axis is the percentage at day 7. The green dot identifies the sgNT control. Genes that scored positive in the screen are highlighted in red.

(C) Relative cell number of LNCaP/AR cells transduced with annotated guide RNAs, normalized to shNT + sgNT + Veh group. Cells were treated with 10 μM enzalutamide (Enz) or DMSO (Veh) for 7 days and cell numbers were counted. Mean ± SEM is presented, and p values were calculated by multiple t tests, with three biological replicates in each group.

(D) Relative gene expression level of the four TF genes in LNCaP/AR cells transduced with annotated inducible shRNAs at various time points. Mean ± SEM is presented, p values were calculated by two-way ANOVA, all compared with 0 h, with three technical replicates in each group. ****p < 0.0001, ***p < 0.001, **p < 0.01, *p < 0.05.

See also Figure S6 and Table S5.

(*KRT5* and *TP63*), and nearly all showed upregulation of genes, such as *SNAI2*, *TWIST1*, *SNAI1*, and *ZEB1* that specify epithelial to mesenchymal transition (EMT). Intriguingly, these changes in lineage gene expression were rapid (evident within only 48 h after doxycycline-inducible *CHD1* knockdown) and reversible (Figures S7E and S7F). Collectively, we propose that *CHD1* loss establishes an altered and plastic chromatin landscape which, in the face of stresses, such as antiandrogen therapy, enables resistant subclones to emerge through activation of alternative, non-luminal lineage programs that reduce dependence on AR.

GR Inhibition Restores Enzalutamide Sensitivity in *CHD1*-Deficient Tumors with Increased GR Expression

Identification of GR as one of the four critical TFs upregulated by *CHD1* loss was of particular interest based on previous reports implicating GR in enzalutamide resistance (Arora et al., 2013; Isikbay et al., 2014, Li et al., 2017) and led us to reexamine the molecular basis of GR upregulation in LREX cells, a previously reported enzalutamide-resistant subline of LNCaP/AR cells (Arora et al., 2013). Remarkably, *CHD1* mRNA and protein levels were significantly lower in LREX compared with LNCaP/AR cells (Figures S8A and S8B). We also found robust upregulation of GR mRNA (*NR3C1*) and protein (Figures 8A and 8B), as well as downstream GR target genes (*SGK1* and *NPC1*), across a panel of enzalutamide-resistant xenografts after *CHD1* deletion (by shRNA or CRISPR) (Figure 8A). These findings are notable because *CHD1* loss resulted in increased GR expression without enzalutamide challenge, in contrast to previous work in *CHD1* intact models (Arora et al., 2013; Shah et al., 2017) (Figures 8A, 8B, and S8C).

To determine if sustained GR expression is required to maintain enzalutamide resistance in *CHD1*-deleted tumors with increased GR expression, we pursued both genetic and pharmacologic strategies. First, we knocked down GR in shCHD1-XE-1, the subline with the highest GR level, using two independent GR hairpins and observed substantial growth inhibition *in vitro* (Figures 8C, 8D, and S8D). For pharmacologic inhibition of GR, we turned to inhibitors of BET bromodomain proteins, which we previously reported can re-sensitize *CHD1* intact CRPC tumors with increased GR levels to enzalutamide by inhibiting GR expression (Shah et al., 2017). *In vitro* experiments using two different BET inhibitors (JQ1 and CPI-0610) confirmed that GR expression in *CHD1*-deficient cells is BET dependent (Figure S8E), similar to data in the LREX model (Arora et al., 2013; Shah et al., 2017). Interestingly, the degree of BET-dependent GR expression

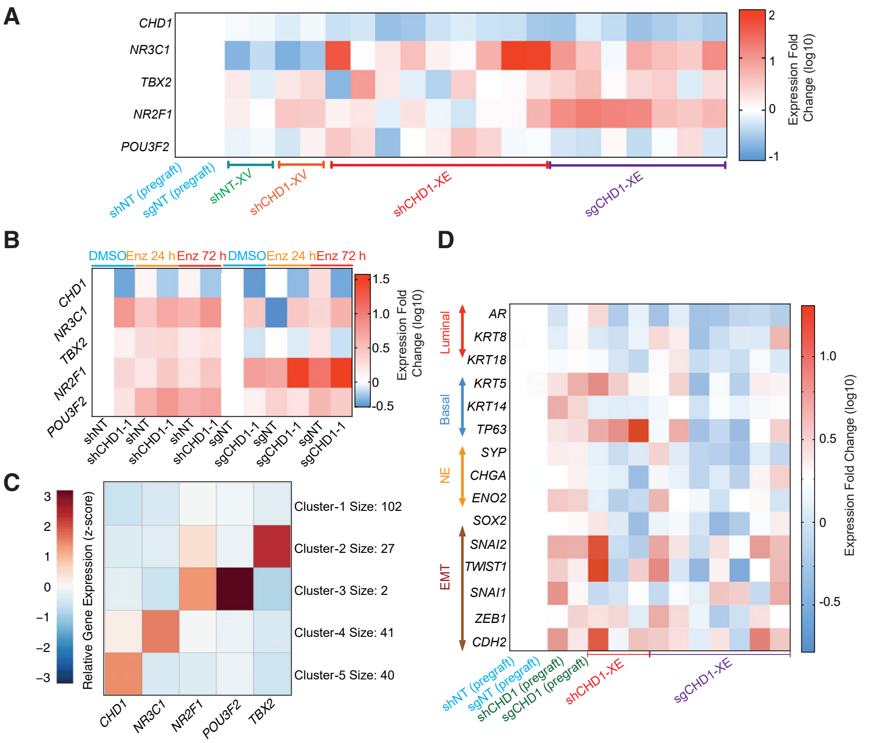

Figure 7. *CHD1* Loss Enhanced Prostate Cancer Cell Heterogeneity and Lineage Plasticity

(A) Heatmap represents the expression fold changes (qPCR) of the top four resistance driver genes and *CHD1* in different xenografts derived cell lines, three technical replicates for each line.

(B) Heatmap represents the expression fold changes of the top four resistant driver genes (qPCR) in shCHD1 cell line treated with 10 μM enzalutamide (Enz) in charcoal-stripped serum medium, three biological replicates for each line.

(C) Unsupervised clustering of 212 patients based on the gene expression level (*Z* score) of *CHD1* and the 4 TFs.

(D) Relative gene expression level (qPCR) of lineage-specific markers and EMT genes in selective shCHD1-XE and sgCHD1-XE cell lines, three technical replicates for each line.

See also Figure S7.

was substantially greater in *CHD1*-deficient cells that had not been previously exposed to enzalutamide (Figure S8E). For *in vivo* experiments, we used CPI-0610 due to its more favorable pharmacologic properties and observed more tumor regressions in mice treated with enzalutamide + CPI-0610 versus either drug alone (Figures S8F and S8G) (Albrecht et al., 2016).

DISCUSSION

It is widely appreciated that the efficacy of targeted cancer therapies can be negatively affected by tumor heterogeneity, particularly in the context of concurrent genomic alterations that can mitigate dependence on the primary oncogenic driver. Cataloging these concurrent alterations in a comprehensive way could better inform patient selection for targeted therapies and provide insight into how to maximize treatment response (Alizadeh et al., 2015; Li et al., 2016). The *in vivo* shRNA library screening strategy reported here, using the next-generation antiandrogen enzalutamide in metastatic CRPC as an example, illustrates the feasibility of this approach as well as the challenges. Two critical learnings were: (1) the use of relatively small shRNA pools (~100 different hairpins) to ensure adequate representation of each hairpin and (2) the decision to perform multiple independent tumor inoculations (10 per pool). The latter decision allowed us to eliminate bystander shRNAs that are enriched solely on the basis of the stochastic growth of individual cells that can contribute disproportionately to the final composition of the tumor (sometimes called jackpot clones). The wisdom of this decision is apparent in the fact that at least three of the eight hits were validated in secondary screens. This approach mandates use of a larger number of animals, but this can be balanced by using smaller, focused libraries (such as the prostate deletome described here) instead of whole genome libraries.

A major insight from our characterization of how *CHD1* loss promotes enzalutamide resistance is the role of an altered chromatin landscape in establishing a cell state that enables more rapid adaptation to environmental stresses, such as antiandrogen therapy than can occur in *CHD1*-intact tumor cells. One consequence of this "cell state model" is the opportunity for multiple different mechanisms of resistance to arise, as illustrated by the four different TFs identified here (Figure 8E). This mechanism has parallels with work in small-cell lung cancer showing altered chromatin landscapes in primary versus metastatic tumors due to genomic amplification of the *NFIB*, which encodes a TF that promotes neuroendocrine differentiation through chromatin pioneering activity (Denny et al., 2016; Yang et al., 2018). Such epigenetic reorganization can also be observed in hematological malignancies (Hassan et al., 2017).

Although this study was focused solely on identifying enzalutamide resistance mechanisms linked to *CHD1* loss, it is remarkable that all four of the TFs identified have been previously implicated in advanced prostate cancer progression. GR is intriguing in light of previous work showing that GR upregulation is an adaptive resistance mechanism (Arora et al., 2013). Indeed, reexamination of those data, in light of these findings, suggests that loss of *CHD1* may be the mechanism of GR upregulation in these earlier models. BRN2 is similarly intriguing based on recent evidence that this neural TF drives neuroendocrine differentiation of tumor cells and thereby promotes enzalutamide resistance through loss of luminal lineage features (Bishop et al., 2017). TBX2, a T-box family TF, has been shown to induce EMT (reduced E-cadherin, increased N-cadherin) and WNT signaling, resulting in enhanced metastasis in prostate cancer models (Du et al., 2017; Nandana et al., 2017). Finally, the orphan nuclear receptor NR2F1 has been linked to tumor cell dormancy in prostate cancer through induction of pluripotency genes, such as *SOX2* and *NANOG* (Sosa et al., 2015).

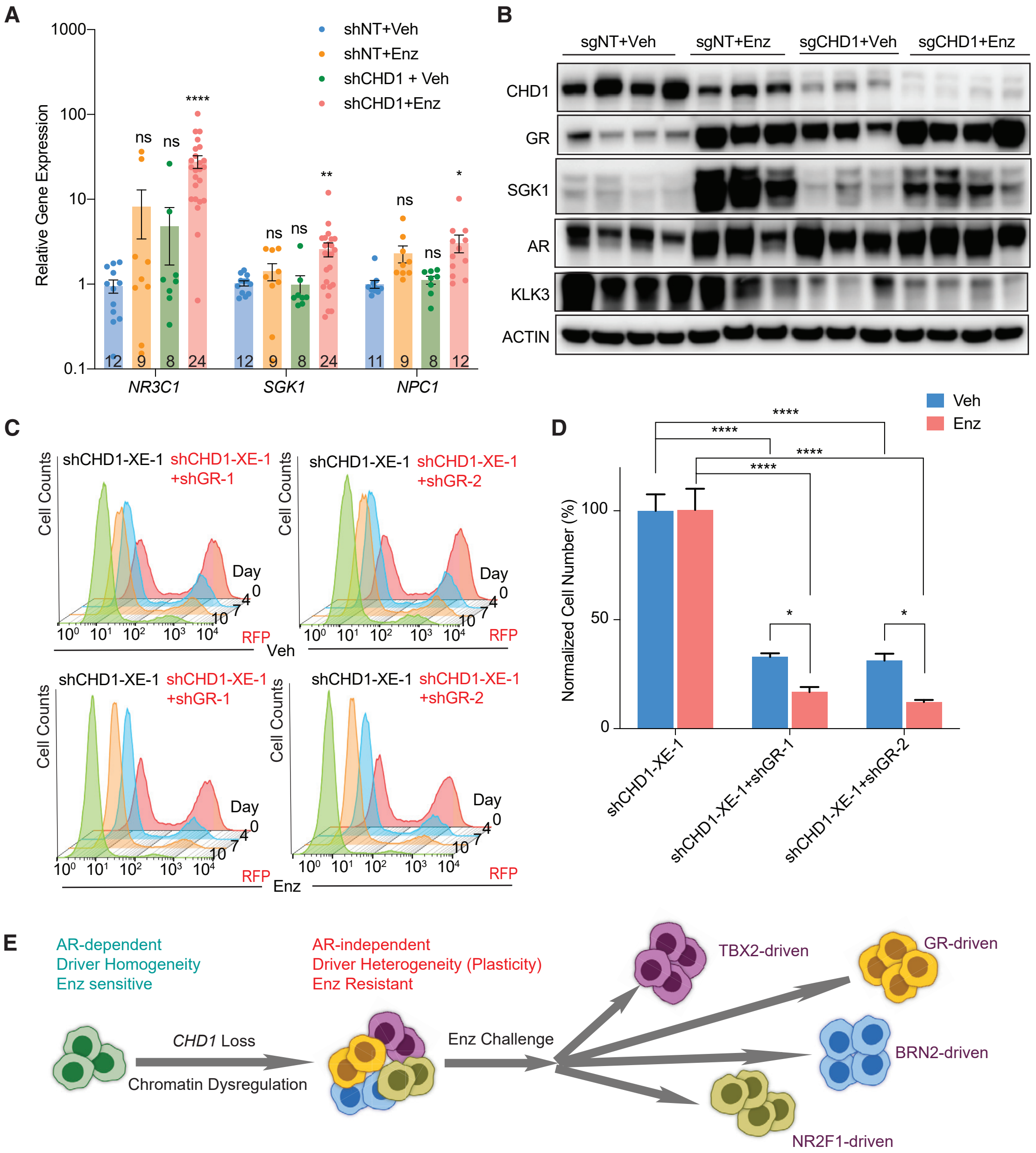

Figure 8. GR Inhibition Has Significant Antitumor Effect on Antiandrogen-Resistant Tumors with *CHD1* Loss

(A) Relative gene expression of *NR3C1* and GR target genes in tumors collected from LNCaP/AR xenografts, all normalized and compared with shNT + Veh group. Mean ± SEM is presented. p values were calculated using two-way ANOVA, and numbers of biological replicates are presented.

(B) Western blot showing AR, GR, and their downstream target genes in xenografted LNCaP/AR tumors. For (A) and (B), Enz denotes enzalutamide at 10 mg/kg from day 1 of grafting. Veh denotes 0.5% CMC + 0.1% Tween 80.

(C) Histograms of representative FACS-based competition assay showing the distribution of shCHD1-XE-1 cells (RFP-negative) versus shCHD1-XE-1 cells transduced with shGR (RFP-positive). The distributions on different days are presented in different colors.

(D) Relative cell number of shCHD1-XE-1 cells transduced with annotated inducible shRNAs, normalized to shCHD1-XE-1 + Veh. Cells were treated with 250 ng/mL doxycycline for 48 h, and then 7 days of 10 μM enzalutamide (Enz) or DMSO (Veh) before cell numbers were counted. Mean ± SEM is presented, and p values were calculated by two-way ANOVA, with three biological replicates in each group.

(E) Model depicting the chromatin dysregulation (plasticity) and antiandrogen resistance in mCRPC due to *CHD1* loss.

For all panels, ****$p < 0.0001$, ***$p < 0.001$, **$p < 0.01$, *$p < 0.05$. See also Figure S8.

Our data demonstrate that *CHD1* loss in CRPC promotes a state of intratumoral heterogeneity, but further work is needed to determine whether these heterogeneous mechanisms function independently or collaboratively. It is worth noting that pluripotency genes, such as *SOX2* have been implicated in several examples of lineage plasticity, including those mediated by

BRN2 and NR2F1, as well as in other examples, such as *RB1* and *TP53* loss (Ku et al., 2017; Mu et al., 2017; Park et al., 2018). Single-cell analysis should bring greater clarity to this heterogeneity, including the possibility that these TFs function in collaborative, hierarchical signaling networks (Goldman et al., 2019).

It is important to place our model of how *CHD1* loss promotes antiandrogen resistance mechanisms in the context of previous work on *CHD1* in prostate cancer. First, it is clear that *Chd1* deletion alone in the mouse prostate is not sufficient to induce cancer (Augello et al., 2019; Shenoy et al., 2017); however, cancers do emerge after co-deletion of *Map3k7* (Rodrigues et al., 2015) or *Pten* (Augello et al., 2019). Conversely, *CHD1* is reported to have a synthetic lethal interaction with *PTEN* in some breast and PCa models (Zhao et al., 2017), presumably due to context-specific effects. Intriguingly, $Chd1^{-/-}$;$Map3k7^{-/-}$ prostate cancers have neuroendocrine features, consistent with our observation that *CHD1* loss can promote expression of aberrant lineage programs. Chromatin immunoprecipitation sequencing studies of the *CHD1* and AR cistromes suggest a regulatory role for CHD1, which directs (or restricts) AR to canonical target genes in normal prostate tissue (Augello et al., 2019). This pattern is disrupted in the setting of *CHD1* loss, where aberrant AR cistromes are observed that more closely resemble those seen in prostate cancers (Augello et al., 2019). Collectively, the phenotypes of neuroendocrine gene expression and altered AR cistromes are consistent with our data showing that *CHD1* loss establishes an altered chromatin landscape, which enables activation of aberrant lineage programs as a mechanism to escape antiandrogen therapy.

In closing, it is worth considering the clinical implications of *CHD1* loss in prostate cancer. Our analysis of a limited cohort suggests that CRPC patients with low *CHD1* expression respond poorly to next-generation antiandrogen therapies. It will be important to validate this finding with a larger cohort, with inclusion of patients with genomic *CHD1* deletion as these were underrepresented in our study.

STAR★METHODS

Detailed methods are provided in the online version of this paper and include the following:

- KEY RESOURCES TABLE
- LEAD CONTACT AND MATERIALS AVAILABILITY
- EXPERIMENTAL MODEL AND SUBJECT DETAIL
  - SCID Mouse *In Vivo* Xenografts
  - Human Prostate Cancer Cell Lines and Mouse Organoids
  - shRNA and CRISPR Model Generation
- METHOD DETAILS
  - Generation of the Human PCa Deletome and Construction of the miR-E shRNA Library
  - *In Vivo* shRNA Mediated Screen and HiSeq
  - Individual Plasmid Construction and Virus Production
  - FACS-Based Growth Competition Assay
  - Cell Growth Assay, Cell Viability Assays and Dose Response Curve
  - Gene Expression Assay by qPCR
  - Western Blot
  - Immunofluorescence (IF)
  - Immunohistochemistry (IHC)
  - FACS-based Functional Screen Mediated by CRISPR/Cas9
- QUANTIFICATION AND STATISTICAL ANALYSIS
  - Statistics Methods
  - Analysis of Human Prostate Cancer Dataset
  - Sample and library preparation for RNA-seq and ATAC-seq
  - Analysis of RNA-seq Data
  - GO Analysis
  - GSEA Analysis
  - Analysis of ATAC-seq Data
  - Differential Binding Analysis
  - ATAC-seq Differential Peak and RNA-Seq Fold Change CDF Plots
  - Annotation and Differential Motif Detection
  - Predicting Driver TFs Using RNA-seq and ATAC-seq Data
  - Generating Density Heatmaps and Profiles
- DATA AND CODE AVAILABILITY

SUPPLEMENTAL INFORMATION

Supplemental Information can be found online at https://doi.org/10.1016/j.ccell.2020.03.001.

ACKNOWLEDGMENTS

We thank the cBioPortal, SU2C, and TCGA for providing genomic and transcriptomic data. We thank A.Viale, N. Socci, D. Nabors, and the MSKCC Integrated Genomics Operation for assistance with the library HiSeq. We thank A. Heguy and the NYU Genome Technology Center for assistance with the RNA-seq and ATAC-seq. We thank J. Zuber for providing retroviral- and lentiviral miR-E-based expression vectors, P. Chi and E. Wong for providing *CHD1*-expressing vectors, E. Lee, D.P. Yun, and H.A. Chen for providing constructs, W. Wu for all the artwork. We thank G. Hannon for critical discussion and feedback. This work was supported or partially supported by: the National Cancer Institute (NCI) and National Institutes of Health (NIH), USA (R00CA218885-04 to P.M., R01CA155169–04 and R01CA19387-01 to C.L.S., F99CA223063 to Z.Z., P30CA008748 to E.d.S., U54OD020355 to S.W.L. and E.d.S., P30CA034196 to S.L., R35CA197311 to J.T.M., and 1R01MH117406 to C.E.M.), Department of Defense, USA (PC170900 to P.M.), Cancer Prevention Research Institute (CPRIT), USA (RR170050 to P.M., RP160249 to J.T.M., and RP150596 to S.B. and V.M.), Prostate Cancer Foundation, USA (17YOUN12 to P.M.), Welch Foundation, USA (I-2005-20190330 to P.M. and I-1961-20180324 to J.T.M.), UTSW Deborah and W.A. Tex Moncrief, Jr. Scholar in Medical Research Award, USA (to P.M.), UTSW Harold C. Simmons Cancer Center Pilot Award, USA (to P.M.), Howard Hughes Medical Institute, USA (DT0712 to C.L.S., J.T.M., and S.L.W.), NIH/NCI/MSKCC Spore in Prostate Cancer, USA (P50 CA092629-14 to C.L.S.), NCI/MSKCC Support Grant/Core Grant, USA (P30CA008748-49 and P3CA008748-49-S2 to C.L.S.), the Starr Cancer Consortium, USA (I9-A9-071 to C.E.M.), the Vallee Foundation, USA (to C.E.M.), the WorldQuant Foundation, USA (to C.E.M.), the Pershing Square Sohn Cancer Research Alliance, USA (to C.E.M.), the Leukemia and Lymphoma Society, USA (LLS 9238-16 and LLS-MCL-982 to C.E.M.), the Jackson Laboratory New Investigator Award, USA (to S.L.), the Director's Innovation Fund, USA (to S.L.), and the NCATS (UL1 TR001866 to Y.L.).

AUTHOR CONTRIBUTIONS

C.L.S. and P.M. conceived the project. Z.Z., C.Z., X.L., P.M., and C.L.S. oversaw the project, designed experiments, and interpreted data. P.M. and C.L.S co-wrote the manuscript. K.N. and L.M. edited the manuscript. P.M., S.L., Y.L., and C.E.M. established the deletome. A.L. designed the shRNA library.

E.H., Z.C., and C.-H.H. constructed the shRNA library. Z.Z., C.Z., X.L., E.H., S.D., K.N., Y.S.L., C.W., and N.J. cloned all other plasmid constructs. Z.Z., X.L., N.J., J.W., D.C., X.C., and E.d.S. performed *in vivo* experiments. C.-C.C., S.B., V.M., J.T.M., and P.M. analyzed the library results. S.B., V.M., C.-C.C., and P.M. performed bioinformatic analysis. Z.Z., C.Z., X.L., S.D., C.R.T., and L.M. performed competition assays. Z.Z., C.Z., X.L., and S.D. established the tumor-derived cell lines. X.L. and S.D. performed inducible shCHD1 and *CHD1* rescue experiments. X.L. performed immunofluorescence and immunohistochemistry. X.L., S.D., C.W., and C.R.T. performed dose-response experiments. S.D. performed proliferation assays. C.Z., X.L., and C.W. performed the CRISPR library screen. Z.Z., C.Z., X.L., S.D., E.H., and C.W. performed western blots and qPCR. W.A., Y.Z., Z.Z., E.L., and P.M. performed clinical data analysis. Y.Z. examined all the statistic tests. S.W.L. supervised the construction of the library and offered critical feedback. C.L.S. and P.M. are the corresponding authors of this manuscript.

DECLARATION OF INTERESTS

C.L.S. and J.W. are co-inventors of enzalutamide and apalutamide and may be entitled to royalties. C.L.S. serves on the Board of Directors of Novartis and is a co-founder of ORIC Pharm. He is a science advisor to Agios, Beigene, Blueprint, Column Group, Foghorn, Housey Pharma, Nextech, KSQ, Petra, and PMV. He was a co-founder of Seragon, purchased by Genentech/Roche in 2014. S.W.L. is a founder and member of the scientific advisory board of ORIC Pharmaceuticals, Blueprint Medicines, and Mirimus, Inc.; he is also on the scientific advisory board of PMV Pharmaceuticals, Constellation Pharmaceuticals, and Petra Pharmaceuticals. W.A. reports consulting for Clovis Oncology, Janssen, MORE Health, and ORIC Pharmaceuticals. He received honoraria from CARET and travel accommodations from GlaxoSmith Kline, Clovis Oncology, and ORIC Pharmaceuticals. C.E.M is a co-founder and board member for Biotia and Onegevity Health, as well as an advisor for Genpro and Karius.

Received: April 16, 2019
Revised: November 4, 2019
Accepted: February 28, 2020
Published: March 26, 2020

REFERENCES

Abida, W., Armenia, J., Gopalan, A., Brennan, R., Walsh, M., Barron, D., Danila, D., Rathkopf, D., Morris, M., Slovin, S., et al. (2017). Prospective genomic profiling of prostate cancer across disease states reveals germline and somatic alterations that may affect clinical decision making. JCO Precis Oncol. *2017*, https://doi.org/10.1200/PO.17.00029.

Abida, W., Cyrta, J., Heller, G., Prandi, D., Armenia, J., Coleman, I., Cieslik, M., Benelli, M., Robinson, D., Allen, E.M., et al. (2019). Genomic correlates of clinical outcome in advanced prostate cancer. Proc. Natl. Acad Sci U S A *116*, 11428–11436.

Albrecht, B.K., Gehling, V.S., Hewitt, M.C., Vaswani, R.G., Côtô, A., Leblanc, Y., Nasveschuk, C.G., Bellon, S., Bergeron, L., Campbell, R., et al. (2016). Identification of a benzoisoxazoloazepine inhibitor (CPI-0610) of the bromodomain and extra-terminal (BET) family as a candidate for human clinical trials. J. Med. Chem. *59*, 1330–1339.

Alizadeh, A.A., Aranda, V., Bardelli, A., Blanpain, C., Bock, C., Borowski, C., Caldas, C., Califano, A., Doherty, M., Elsner, M., et al. (2015). Toward understanding and exploiting tumor heterogeneity. Nat. Med. *21*, 846–853.

Arora, V.K., Schenkein, E., Murali, R., Subudhi, S.K., Wongvipat, J., Balbas, M.D., Shah, N., Cai, L., Efstathiou, E., Logothetis, C., et al. (2013). Glucocorticoid receptor confers resistance to antiandrogens by bypassing androgen receptor blockade. Cell *155*, 1309–1322.

Augello, M.A., Liu, D., Deonarine, L.D., Robinson, B.D., Huang, D., Stelloo, S., Blattner, M., Doane, A.S., Wong, E., Chen, Y., et al. (2019). CHD1 loss alters AR binding at lineage-specific enhancers and modulates distinct transcriptional programs to drive prostate tumorigenesis. Cancer Cell *35*, 817–819.

Bailey, T.L., Boden, M., Buske, F.A., Frith, M., Grant, C.E., Clementi, L., Ren, J., Li, W.W., and Noble, W.S. (2009). MEME SUITE: tools for motif discovery and searching. Nucleic Acids Res *37*, W202–W208.

Bailey, T.L., Johnson, J., Grant, C.E., and Noble, W.S. (2015). The MEME suite. Nucleic Acids Res. *43*, W39–W49.

Balbas, M.D., Evans, M.J., Hosfield, D.J., Wongvipat, J., Arora, V.K., Watson, P.A., Chen, Y., Greene, G.L., Shen, Y., and Sawyers, C.L. (2013). Overcoming mutation-based resistance to antiandrogens with rational drug design. eLife *2*, e00499.

Barbieri, C.E., Baca, S.C., Lawrence, M.S., Demichelis, F., Blattner, M., Theurillat, J.-P., White, T.A., Stojanov, P., Allen, E., Stransky, N., et al. (2012). Exome sequencing identifies recurrent SPOP, FOXA1 and MED12 mutations in prostate cancer. Nat. Genet. *44*, 685–689.

Barnes S.D., Ruess H., and Malladi V.S. (2019). BICF ATAC-seq Analysis Workflow (Version publish_1.0.0). Zenodo. http://doi.org/10.5281/zenodo.3526149.

Beer, T.M., Armstrong, A.J., Rathkopf, D.E., Loriot, Y., Sternberg, C.N., Higano, C.S., Iversen, P., Bhattacharya, S., Carles, J., Chowdhury, S., et al. (2014). Enzalutamide in metastatic prostate cancer before chemotherapy. N. Engl. J. Med. *371*, 424–433.

Beltran, H., Prandi, D., Mosquera, J.-M., Benelli, M., Puca, L., Cyrta, J., Marotz, C., Giannopoulou, E., Chakravarthi, B.V., Varambally, S., et al. (2016). Divergent clonal evolution of castration-resistant neuroendocrine prostate cancer. Nat. Med. *22*, 298–305.

Beltran, H., Rickman, D., Park, K., Chae, S., Sboner, A., MacDonald, T., Wang, Y., Sheikh, K., Terry, S., Tagawa, S., et al. (2011). Molecular characterization of neuroendocrine prostate cancer and identification of new drug targets. Cancer Discov. *1*, 487–495.

Bishop, J.L., Thaper, D., Vahid, S., Davies, A., Ketola, K., Kuruma, H., Jama, R., Nip, K., Angeles, A., Johnson, F., et al. (2017). The master neural transcription factor BRN2 is an androgen receptor-suppressed driver of neuroendocrine differentiation in prostate cancer. Cancer Discov. *7*, 54–71.

Boysen, G., Rodrigues, D., Rescigno, P., Seed, G., Dolling, D.I., Riisnaes, R., Crespo, M., Zafeiriou, Z., Sumanasuriya, S., Bianchini, D., et al. (2018). SPOP mutated/CHD1 deleted lethal prostate cancer and abiraterone sensitivity. Clin. Cancer Res. *24*, 5585–5593.

Chen, Y., Chi, P., Rockowitz, S., Iaquinta, P.J., Shamu, T., Shukla, S., Gao, D., Sirota, I., Carver, B.S., Wongvipat, J., et al. (2013). ETS factors reprogram the androgen receptor cistrome and prime prostate tumorigenesis in response to PTEN loss. Nat Med *19*, 1023–1029.

Chen, C.D., Welsbie, D.S., Tran, C., Baek, S., Chen, R., Vessella, R., Rosenfeld, M.G., and Sawyers, C.L. (2003). Molecular determinants of resistance to antiandrogen therapy. Nat. Med. *10*, 33–39.

Denny, S.K., Yang, D., Chuang, C.-H., Brady, J.J., Lim, J., Grüner, B.M., Chiou, S.-H., Schep, A.N., Baral, J., Hamard, C., et al. (2016). Nfib promotes metastasis through a widespread increase in chromatin accessibility. Cell *166*, 328–342.

Du, W.-L., Fang, Q., Chen, Y., Teng, J.-W., Xiao, Y.-S., Xie, P., Jin, B., and Wang, J.-Q. (2017). Effect of silencing the T-Box transcription factor TBX2 in prostate cancer PC3 and LNCaP cells. Mol. Med. Rep. *16*, 6050–6058.

Fellmann, C., Hoffmann, T., dhar, V., Hopfgartner, B., Muhar, M., Roth, M., Lai, D., Barbosa, I.A., Kwon, J., Guan, Y., et al. (2013). An optimized microRNA backbone for effective single-copy RNAi. Cell Rep. *5*, 1704–1713.

Feng, J., Liu, T., Qin, B., Zhang, Y., and Liu, X. (2012). Identifying ChIP-seq enrichment using MACS. Nat. Protoc. *7*, 1728.

Franco, H.L., Nagari, A., Malladi, V.S., Li, W., Xi, Y., Richardson, D., Allton, K.L., Tanaka, K., Li, J., Murakami, S., et al. (2018). Enhancer transcription reveals subtype-specific gene expression programs controlling breast cancer pathogenesis. Genome Res. *28*, 159–170.

Gao, D., Vela, I., Sboner, A., Iaquinta, P.J., Karthaus, W.R., Gopalan, A., Dowling, C., Wanjala, J.N., Undvall, E.A., Arora, V.K., et al. (2014). Organoid cultures derived from patients with advanced prostate cancer. Cell *159*, 176–187.

Garraway, L.A., Widlund, H.R., Rubin, M.A., Getz, G., Berger, A.J., Ramaswamy, dhar, Beroukhim, R., Milner, D.A., Granter, S.R., Du, J., et al. (2005). Integrative genomic analyses identify MITF as a lineage survival oncogene amplified in malignant melanoma. Nature *436*, 117.

Gaspar-Maia, A., Alajem, A., Polesso, F., dharan, R., Mason, M.J., Heidersbach, A., Ramalho-Santos, J., McManus, M.T., Plath, K., Meshorer, E., et al. (2009). Chd1 regulates open chromatin and pluripotency of embryonic stem cells. Nature *460*, 863–868.

Golden, R.J., Chen, B., Li, T., Braun, J., Manjunath, H., Chen, X., Wu, J., Schmid, V., Chang, T.-C., Kopp, F., et al. (2017). An Argonaute phosphorylation cycle promotes microRNA-mediated silencing. Nature *542*, 197–202.

Goldman, S.L., MacKay, M., Afshinnekoo, E., Melnick, A.M., Wu, S., and Mason, C.E. (2019). The impact of heterogeneity on single-cell sequencing. Front. Genet. *10*, 8.

Grasso, C.S., Wu, Y.-M., Robinson, D.R., Cao, X., anasekaran, S., Khan, A.P., Quist, M.J., Jing, X., Lonigro, R.J., Brenner, C.J., et al. (2012). The mutational landscape of lethal castration-resistant prostate cancer. Nature *487*, 239–243.

Hassan, C., Afshinnekoo, E., Li, S., Wu, S., and Mason, C.E. (2017). Genetic and epigenetic heterogeneity and the impact on cancer relapse. Exp. Hematol. *54*, 26–30.

Heinz, S., Benner, C., Spann, N., Bertolino, E., Lin, Y.C., Laslo, P., Cheng, J.X., Murre, C., Singh, H., and Glass, C.K. (2010). Simple combinations of lineage-determining transcription factors prime *cis*-regulatory elements required for macrophage and B cell identities. Mol. Cell *38*, 576–589.

Holcomb, I.N., Young, J.M., Coleman, I.M., Salari, K., Grove, D.I., Hsu, L., True, L.D., Roudier, M.P., Morrissey, C.M., Higano, C.S., et al. (2009). Comparative analyses of chromosome alterations in soft-tissue metastases within and across patients with castration-resistant prostate cancer. Cancer Res. *69*, 7793–7802.

Huang, S., Gulzar, Z., Salari, K., Lapointe, J., Brooks, J., and Pollack, J. (2011). Recurrent deletion of CHD1 in prostate cancer with relevance to cell invasiveness. Oncogene *31*, 4164–4170.

Isikbay, M., Otto, K., Kregel, S., Kach, J., Cai, Y., Griend, D.J., Conzen, S.D., and Szmulewitz, R.Z. (2014). Glucocorticoid receptor activity contributes to resistance to androgen-targeted therapy in prostate cancer. Horm. Cancer *5*, 72–89.

Karthaus, W.R., Iaquinta, P.J., Drost, J., Gracanin, A., van Boxtel, R., Wongvipat, J., wling, C., Gao, D., Begthel, H., Sachs, N., et al. (2014). Identification of multipotent luminal progenitor cells in human prostate organoid cultures. Cell *159*, 163–175.

Khan, A., Fornes, O., Stigliani, A., Gheorghe, M., Castro-Mondragon, J.A., van der Lee, R., Bessy, A., Cheneby, J., Kulkarni, S.R., and Tan, G. (2017). Jaspar 2018: update of the open-access database of transcription factor binding profiles and its web framework. Nucleic Acids Res. *46*, D1284.

Kim, J.H., anasekaran, S., Mehra, R., Tomlins, S.A., Gu, W., Yu, J., Kumar-Sinha, C., Cao, X., Dash, A., Wang, L., et al. (2007). Integrative analysis of genomic aberrations associated with prostate cancer progression. Cancer Res. *67*, 8229–8239.

Klein, K.A., Reiter, R.E., Redula, J., Moradi, H., Zhu, X., Brothman, A.R., Lamb, D.J., Marcelli, M., Belldegrun, A., Witte, O.N., et al. (1997). Progression of metastatic human prostate cancer to androgen independence in immunodeficient SCID mice. Nat. Med. *3*, 402–408.

Ku, S., Rosario, S., Wang, Y., Mu, P., Seshadri, M., Goodrich, Z.W., Goodrich, M.M., Labbé, D.P., Gomez, E., Wang, J., et al. (2017). Rb1 and Trp53 cooperate to suppress prostate cancer lineage plasticity, metastasis, and antiandrogen resistance. Science *355*, 78–83.

Landt, S.G., Marinov, G.K., Kundaje, A., Kheradpour, P., Pauli, F., Batzoglou, S., Bernstein, B.E., Bickel, P., Brown, J.B., Cayting, P., et al. (2012). ChIP-seq guidelines and practices of the ENCODE and modENCODE consortia. Genome Res *22*, 1813–1831.

Li, J., Alyamani, M., Zhang, A., Chang, K.-H., Berk, M., Li, Z., Zhu, Z., Petro, M., Magi-Galluzzi, C., Taplin, M.-E., et al. (2017). Aberrant corticosteroid metabolism in tumor cells enables GR takeover in enzalutamide resistant prostate cancer. eLife *6*, e20183.

Li, H., and Durbin, R. (2009). Fast and accurate short read alignment with Burrows-Wheeler transform. Bioinformatics *25*, 1754–1760.

Li, S., Garrett-Bakelman, F.E., Chung, S.S., Sanders, M.A., Hricik, T., Rapaport, F., Patel, J., Dillon, R., Vijay, P., Brown, A.L., et al. (2016). Distinct evolution and dynamics of epigenetic and genetic heterogeneity in acute myeloid leukemia. Nat. Med. *22*, 792–799.

Li, H., Handsaker, B., Wysoker, A., Fennell, T., Ruan, J., Homer, N., Marth, G., Abecasis, G., and Durbin, R. (2009). The sequence alignment/map format and SAMtools. Bioinformatics *25*, 2078–2079.

Liao, Y., Smyth, G.K., and Shi, W. (2014). featureCounts: an efficient general purpose program for assigning sequence reads to genomic features. Bioinformatics *30*, 923–930.

Liu, W., Lindberg, J., Sui, G., Luo, J., Egevad, L., Li, T., Xie, C., Wan, M., Kim, S.-T., Wang, Z., et al. (2012). Identification of novel CHD1-associated collaborative alterations of genomic structure and functional assessment of CHD1 in prostate cancer. Oncogene *31*, 3939.

Love, M.I., Huber, W., and Anders, S. (2014). Moderated estimation of fold change and dispersion for RNA-seq data with DESeq2. Genome Biol. *15*, 550.

Martin, M. (2011). Cutadapt removes adapter sequences from high-throughput sequencing reads. Embnet.journa. *17*, https://doi.org/10.14806/ej.17.1.200.

Mi, H., Muruganujan, A., Ebert, D., Huang, X., and Thomas, P.D. (2018). PANTHER version 14: more genomes, a new PANTHER GO-slim and improvements in enrichment analysis tools. Nucleic Acids Res *47*, D419–D426.

Müllner, D. (2013). Fast Hierarchical Clustering Routines for R and Python. R Package Fastcluster Version 1.

Mu, P., Zhang, Z., Benelli, M., Karthaus, W.R., Hoover, E., Chen, C.-C., Wongvipat, J., Ku, S., Gao, D., Cao, Z., et al. (2017). SOX2 promotes lineage plasticity and antiandrogen resistance in TP53- and RB1-deficient prostate cancer. Science *355*, 84–88.

Mudge, J.M., and Harrow, J. (2015). Creating reference gene annotation for the mouse C57BL6/J genome assembly. Mamm. Genome *26*, 366–378.

Nandana, S., Tripathi, M., Duan, P., Chu, C.-Y., Mishra, R., Liu, C., Jin, R., Yamashita, H., Zayzafoon, M., Bhowmick, N.A., et al. (2017). Bone metastasis of prostate cancer can be therapeutically targeted at the TBX2-WNT signaling axis. Cancer Res. *77*, 1331–1344.

Network, C. (2015). The molecular taxonomy of primary prostate cancer. Cell *163*, 1011–1025.

Park, J., Lee, J.K., Sheu, K.M., Wang, L., Balanis, N.G., Nguyen, K., Smith, B.A., Cheng, C., Tsai, B.L., Cheng, D., et al. (2018). Reprogramming normal human epithelial tissues to a common, lethal neuroendocrine cancer lineage. Science *362*, 91–95.

Pelossof, R., Fairchild, L., Huang, C.-H., Widmer, C., edharan, V.T., Sinha, N., Lai, D., Guan, Y., Premsrirut, P.K., Tschaharganeh, D.F., et al. (2017). Prediction of potent shRNAs with a sequential classification algorithm. Nat. Biotechnol. *35*, 350–353.

Pertea, M., Kim, D., Pertea, G.M., Leek, J.T., and Salzberg, S.L. (2016). Transcript-level expression analysis of RNA-seq experiments with HISAT, StringTie and Ballgown. Nat. Protoc. *11*, 1650.

Quinlan, A.R., and Hall, I.M. (2010). BEDTools: a flexible suite of utilities for comparing genomic features. Bioinformatics *26*, 841–842.

R Core Team (2016). R: A Language and Environment for Statistical Computing (R Foundation for Statistical Computing). https://www.R-project.org/.

Ramírez, F., Ryan, D.P., Grüning, B., Bhardwaj, V., Kilpert, F., Richter, A.S., Heyne, S., Dündar, F., and Manke, T. (2016). deepTools2: a next generation web server for deep-sequencing data analysis. Nucleic Acids Res. *44*, W160–W165.

Ren, S., Wei, G.-H., Liu, D., Wang, L., Hou, Y., Zhu, S., Peng, L., Zhang, Q., Cheng, Y., Su, H., et al. (2018). Whole-genome and transcriptome sequencing of prostate cancer identify new genetic alterations driving disease progression. Eur. Urol. *73*, 322–339.

Robinson, D., Allen, E.M., Wu, Y.-M., Schultz, N., Lonigro, R.J., Mosquera, J.-M., Montgomery, B., Taplin, M.-E., Pritchard, C.C., Attard, G., et al.

(2015). Integrative clinical genomics of advanced prostate cancer. Cell *161*, 1215–1228.

Rodrigues, L., Rider, L., Nieto, C., Romero, L., Karimpour-Fard, A., Loda, M., Luciai, M.S., Wu, M., Shi, L., Cimic, A., et al. (2015). Coordinate loss of MAP3K7 and CHD1 promotes aggressive prostate cancer. Cancer Res. *75*, 1021–1034.

Ryan, C.J., Smith, M.R., de Bono, J.S., Molina, A., Logothetis, C.J., de Souza, P., Fizazi, K., Mainwaring, P., Piulats, J.M., Ng, S., et al. (2013). Abiraterone in metastatic prostate cancer without previous chemotherapy. N. Engl. J. Med. *368*, 138–148.

Sequist, L.V., Waltman, B.A., Dias-Santagata, D., Digumarthy, S., Turke, A.B., Fidias, P., Bergethon, K., Shaw, A.T., Gettinger, S., Cosper, A.K., et al. (2011). Genotypic and histological evolution of lung cancers acquiring resistance to EGFR inhibitors. Sci. Transl Med. *3*, 75ra26.

Shah, N., Wang, P., Wongvipat, J., Karthaus, W.R., Abida, W., Armenia, J., Rockowitz, S., Drier, Y., Bernstein, B.E., Long, H.W., et al. (2017). Regulation of the glucocorticoid receptor via a BET-dependent enhancer drives antiandrogen resistance in prostate cancer. eLife *6*, e27861.

Shenoy, T., Boysen, G., Wang, M., Xu, Q., Guo, W., Koh, F., Wang, C., Zhang, L., Wang, Y., Gil, V., et al. (2017). CHD1 loss sensitizes prostate cancer to DNA damaging therapy by promoting error-prone double-strand break repair. Ann. Oncol. *28*, 1495–1507.

Shi, W., Wang, D., Yuan, X., Liu, Y., Guo, X., Li, J., and Song, J. (2019). Glucocorticoid receptor-IRS-1 axis controls EMT and the metastasis of breast cancers. J. Mol. Cell Biol. *11*, 1042–1055.

Smith, M.R., Saad, F., Chowdhury, S., Oudard, S., Hadaschik, B.A., Graff, J.N., Olmos, D., Mainwaring, P.N., Lee, J., Uemura, H., et al. (2018). Apalutamide treatment and metastasis-free survival in prostate cancer. N. Engl. J. Med. *378*, 1408–1418.

Sosa, M., Parikh, F., Maia, A., Estrada, Y., Bosch, A., Bragado, P., Ekpin, E., George, A., Zheng, Y., Lam, H.-M., et al. (2015). NR2F1 controls tumour cell dormancy via SOX9- and RARβ-driven quiescence programmes. Nat. Commun. *6*, 6170.

Stark, R., and Brown, G. (2011). DiffBind: Differential Binding Analysis of ChIP-Seq Peak Data. R Package Version 100. http://bioconductor.org/packages/release/bioc/vignettes/DiffBind/inst/doc/DiffBind.pdf

Tarasov, A., Vilella, A.J., Cuppen, E., Nijman, I.J., and Prins, P. (2015). Sambamba: fast processing of NGS alignment formats. Bioinformatics *31*, 2032–2034.

Taylor, B.S., Barretina, J., Socci, N.D., Decarolis, P., Ladanyi, M., Meyerson, M., Singer, S., and Sander, C. (2008). Functional copy-number alterations in cancer. PLos One *3*, e3179.

Taylor, B.S., Schultz, N., Hieronymus, H., Gopalan, A., Xiao, Y., Carver, B.S., Arora, V.K., Kaushik, P., Cerami, E., Reva, B., et al. (2010). Integrative genomic profiling of human prostate cancer. Cancer Cell *18*, 11–22.

Tran, C., Ouk, S., Clegg, N., Chen, Y., Watson, P., Arora, V., Wongvipat, J., ith-Jones, P., Yoo, D., Kwon, A., et al. (2009). Development of a second-generation antiandrogen for treatment of advanced prostate cancer. Science *324*, 787–790.

Tripodi, I.J., Allen, M.A., and Dowell, R.D. (2018). Detecting differential transcription factor Activity from ATAC-seq data. Molecules *23*, 1136.

Varghese, F., Bukhari, A.B., Malhotra, R., and De, A. (2014). IHC profiler: an open source plugin for the quantitative evaluation and automated scoring of immunohistochemistry images of human tissue samples. PLoS One *9*, e96801.

Watson, P.A., Arora, V.K., and Sawyers, C.L. (2015). Emerging mechanisms of resistance to androgen receptor inhibitors in prostate cancer. Nat. Rev. Cancer *15*, 701–711.

Wheeler, D.B., Zoncu, R., Root, D.E., batini, D., and Sawyers, C.L. (2015). Identification of an oncogenic RAB protein. Science *350*, 211–217.

Yang, D., Denny, S.K., Greenside, P.G., Chaikovsky, A.C., Brady, J.J., Ouadah, Y., Granja, J.M., Jahchan, N.S., Lim, J., Kwok, S., et al. (2018). Intertumoral heterogeneity in SCLC is influenced by the cell type of origin. Cancer Discov. *8*, 1316–1331.

Zack, G., Rogers, W., and Latt, S. (1977). Automatic measurement of sister chromatid exchange frequency. J. Histochem. Cytochem. Official J. Histochem. Soc. *25*, 741–753.

Zhao, D., Lu, X., Wang, G., Lan, Z., Liao, W., Li, J., Liang, X., Chen, J., Shah, S., Shang, X., et al. (2017). Synthetic essentiality of chromatin remodelling factor CHD1 in PTEN-deficient cancer. Nature *542*, 484.

Zuber, J., McJunkin, K., Fellmann, C., Dow, L.E., Taylor, M.J., Hannon, G.J., and Lowe, S.W. (2010). Toolkit for evaluating genes required for proliferation and survival using tetracycline-regulated RNAi. Nat. Biotechnol. *29*, 79–83.

Zuber, J., Shi, J., Wang, E., Rappaport, A.R., Herrmann, H., Sison, E.A., Magoon, D., Qi, J., Blatt, K., Wunderlich, M., et al. (2011). RNAi screen identifies Brd4 as a therapeutic target in acute myeloid leukaemia. Nature *478*, 524–528.

STAR★METHODS

KEY RESOURCES TABLE

REAGENT or RESOURCE	SOURCE	IDENTIFIER
Antibodies		
CHD1 (D8C2) Rabbit mAb	Cell Signaling	Cat #4351; RRID: AB_11179073
AR Antibody (N-20)	Santa Cruz	sc-816; RRID: AB_1563391
PSA/KLK3 (D6B1) XP® Rabbit mAb	Cell Signaling	Cat # 5365; RRID: AB_2797609
PMEPA1 Antibody (P-15)	Santa Cruz	Cat # sc-85829; RRID: AB_2252615
STEAP Antibody (B-4)	Santa Cruz	Cat # sc-271872; RRID: AB_10707830
β-Actin (13E5) Rabbit mAb	Cell Signaling	Cat # 4970; RRID: AB_10694076
Glucocorticoid Receptor (D6H2L) XP® Rabbit mAb	Cell Signaling	Cat #12041; RRID: AB_2631286
SGK1 (D27C11) Rabbit mAb	Cell Signaling	Cat #12103; RRID: AB_2687476
c-Myc (D84C12) Rabbit mAb	Cell Signaling	Cat #5605; RRID: AB_1903938
NR2F1 (H8132) mouse mAb	R&D Systems, Inc.	Cat PP-H8132-00; RRID: AB_2155494
Alexa Fluor® 488 AffiniPure Goat Anti-Mouse IgG (H+L)	Jackson Immunoresearch	Cat: 115-545-003; RRID: AB_2338840
Alexa Fluor® 594 AffiniPure Goat Anti-Rabbit IgG (H+L)	Jackson Immunoresearch	Cat: 111-585-003; RRID: AB_2338059
VECTASTAIN® ABC HRP Kit- Rabbit IgG	Peroxidase	Cat:PK-6101; RRID: AB_2336815
Chemicals, Peptides, and Recombinant Proteins		
Enzalutamide	Selleck Chemicals	S1250
CPI-0610	Selleck Chemicals	S7853
JQ-1	Selleck Chemicals	S7110
GlutaMAXTM Supplement	Gibco	35050061
1M HEPES Solution	Gibco	15630080
100mM Sodium Pyruvate	Gibco	11360-070
Penicillin-streptomycin	Sigma Aldrich	P0781-100ML
Puromycin	Gibco	A1113803
Blasticidin	Gibco	A1113903
Doxycycline	Sigma Aldrich	D3072-1ML
Trizol	Ambion	15596018
20X NuPAGE MES SDS buffer	Novex	NP0002
1X Bolt Transfer buffer	Novex	BT00061
100% methanol	Thermo Fisher	A412-20
Fetal Bovine Serum, charcoal stripped	Gibco	12-676-029
TrypLE Express	Gibco	12605-010
Transposase enzyme	Illumina Nextera	15028252
Critical Commercial Assays		
SuperScript™ IV VILO™ Master Mix with ezDNase™ Enzyme	Thermo Fisher	11766500
2X PowerUp™ SYBR™ Green Master Mix	Thermo Fisher	A25778
Pierce BCA Protein Assay Kit	Thermo Fisher	23225

(*Continued on next page*)

Continued

REAGENT or RESOURCE	SOURCE	IDENTIFIER
MycoAlertTM PLUS Mycoplasma Detection kit	Lonza	LT07-710
Lipofectamine 2000 Transfection Reagent	Thermo Fisher	11668500
Qiagen MinElute PCR purification kit	Qiagen	28004
ImmPACT™ DAB Peroxidase (HRP) Substrate	LSBio	LS-J1075-120
CellTiter-Glo luminescent cell viability assay	Promega	cat #7570
Deposited Data		
Library Hi-seq	GEO	GSE127957
RNA-seq	GEO	GSE126917
ATAC-seq	GEO	GSE127241
Experimental Models: Cell Lines		
LNCaP/AR	Chen et al., 2003	N/A
LREX	Arora et al., 2013	N/A
CWR22Pc	Mu et al., 2017	N/A
LAPC4	ATCC	N/A
Pten$^{-/-}$ mouse organoid	Chen et al., 2013	N/A
E006AA	ATCC	N/A
shCHD1-XE-1	This paper	N/A
shCHD1-XE-2	This paper	N/A
shCHD1-XE-3	This paper	N/A
shCHD1-XE-4	This paper	N/A
shCHD1-XE-5	This paper	N/A
shCHD1-XE-6	This paper	N/A
shCHD1-XE-7	This paper	N/A
shCHD1-XE-8	This paper	N/A
shCHD1-XE-9	This paper	N/A
sgCHD1-XE-1	This paper	N/A
sgCHD1-XE-2	This paper	N/A
sgCHD1-XE-3	This paper	N/A
sgCHD1-XE-4	This paper	N/A
sgCHD1-XE-5	This paper	N/A
sgCHD1-XE-6	This paper	N/A
sgCHD1-XE-7	This paper	N/A
shNT-XV-1	This paper	N/A
shNT-XV-2	This paper	N/A
shCHD1-XV-1	This paper	N/A
shCHD1-XV-2	This paper	N/A
Experimental Models: Organisms/Strains		
C.B-*Igh*-1^{b}/IcrTac-*Prkdc*^{scid} mouse	Taconic	CB17SC-M
Oligonucleotides		
LEPG-shNT:TGCTGTTGACAGTGAGCGCAGGAATTATA ATGCTTATCTATAGTGAAGCCACAGATGTATAGATAAG CATTATAATTCCTATGCCTACTGCCTCGGA	This paper	N/A
LEPG-shCHD1-1: TGCTGTTGACAGTGAGCGACAGGTTAACA TTTTAGATAAATAGTGAAGCCACAGATGTATTTATCTAAAATG TTAACCTGGTGCCTACTGCCTCGGACTTCAAGGGGCTAGAATTC	This paper	N/A
LEPG-shCHD1-2: TGCTGTTGACAGTGAGCGACAGGAAATGGA TATAGATGAATAGTGAAGCCACAGATGTATTCATCTATATCCAT TTCCTGGTGCCTACTGCCTCGGACTTCAAGGGGCTAGAATTC	This paper	N/A

(*Continued on next page*)

Continued

REAGENT or RESOURCE	SOURCE	IDENTIFIER
LEPG-shCHD1-3: TGCTGTTGACAGTGAGCGCAACGTTATATATGACAAATTATA GTGAAGCCACAGATGTATAATTTGTCATATATAACGTTTTGCC TACTGCCTCGGACTTCAAGGGGCTAGAATTC	This paper	N/A
LEPG-shCHD1-4: TGCTGTTGACAGTGAGCGACAGGAGAGATTCAGTATTTAATAG TGAAGCCACAGATGTATTAAATACTGAATCTCTCCTGGTGCCT ACTGCCTCGGACTTCAAGGGGCTAGAATTC	This paper	N/A
LEPG-shCHD1-5: TGCTGTTGACAGTGAGCGCTAGGCGGTTTATCAAGAGCTATA GTGAAGCCACAGATGTATAGCTCTTGATAAACCGCCTAATGC CTACTGCCTCGGACTTCAAGGGGCTAGAATTC	This paper	N/A
LT3CEPIR-shGR-1: TGCTGTTGACAGTGAGCGCCCAAAGCAGTTTCACTCTCAAT AGTGAAGCCACAGATGTATTGAGAGTGAAACTGCTTTGGAT GCCTACTGCCTCGGA	This paper	N/A
LT3CEPIR-shGR-2: TGCTGTTGACAGTGAGCGAAAGCTGTAAAGTTTTCTTCAATAGT GAAGCCACAGATGTATTGAAGAAAACTTTACAGCTTCTGCCT ACTGCCTCGGA	This paper	N/A
lentiCRISPRv2-sgCHD1-1-F: CACCGTCAGCTCCATCAACTTTCGG lentiCRISPRv2-sgCHD1-1-R: AAACCCGAAAGTTGATGGAGCTGAC	This paper	N/A
lentiCRISPRv2-sgCHD1-2-F: CACCGGATTTATGGATTGTCGGATT lentiCRISPRv2-sgCHD1-2-R: AAACAATCCGACAATCCATAAATCC	This paper	N/A
Additional sgRNA sequences, see Tables S5 and S6	N/A	N/A
Primers, see Table S6	N/A	N/A
**Recombinant DNA**		
pMSCV-miRE-PGK-PuroR-IRES-GFP	Fellmann et al.,2013	LEPG
pRRL-GFP-miRE-PGK-PuroR	Fellmann et al.,2013	SGEP
pRRL-TRE3G-GFP-miRE-PGK-PuroR-IRES-rtTA3	Fellmann et al.,2013	LT3GEPIR
pMSCV-miRE-PGK-PuroR-IRES-mCherry	Mu et al., 2017	LEPC
pRRL-mCherry-miRE-PGK-PuroR	Mu et al., 2017	SCEP
pRRL-TRE3G-mCherry-miRE-PGK-PuroR-IRES-rtTA3	Mu et al., 2017	LT3CEPIR
lentiCRISPR v2	Addgene	Cat #52961
pLKO5.sgRNA.EFS.tRFP	Addgene	Cat #57823
lentiCas9-Blast	Addgene	Cat #52962
**Software and Algorithms**		
HISAT (v 2.0.1)	Pertea et al., 2016	http://ccb.jhu.edu/software/hisat2/index.shtml
Sambamba (v0.6.6)	Tarasov et al., 2015	http://lomereiter.github.io/sambamba/
Featurecount (v1.4.6)	Liao et al., 2014	http://bioinf.wehi.edu.au/featureCounts/
DEseq2 (v1.6.3)	Love et al., 2014	https://bioconductor.org/packages/release/bioc/html/DESeq2.html
PANTHER	Mi et al., 2018	http://www.pantherdb.org
Trimgalore (v0.4.1)	Martin, 2011	https://www.bioinformatics.babraham.ac.uk/projects/trim_galore
BWA (v0.7.12)	Li and Durbin, 2009	http://bio-bwa.sourceforge.net
Samtools (v1.3)	Li et al., 2009	http://samtools.sourceforge.net
BEDTools (v2.26.0)	Quinlan and Hall, 2010	https://bedtools.readthedocs.io/en/latest
MACS (v2.1.0)	Feng et al., 2012	https://github.com/taoliu/MACS
R (v3.3.2) package DiffBind (v2.2.12)	R Core Team, 2016; Stark and Brown, 2011	https://bioconductor.org/packages/release/bioc/html/DiffBind.html

(*Continued on next page*)

Continued

REAGENT or RESOURCE	SOURCE	IDENTIFIER
MEME suite (v4.11.1)	Bailey et al., 2009<br>Bailey et al., 2015	http://meme-suite.org
DAStk (v0.1.5)	Tripodi et al., 2018	https://pypi.org/project/DAStk
HOMER (v4.9)	Heinz et al., 2010	http://homer.ucsd.edu/homer/ngs/annotation.html
deepTools (v2.5.0)	Ramirez et al., 2016	http://homer.ucsd.edu/homer/ngs/annotation.html
hclust	Müllner, 2013	http://danifold.net/fastcluster.html
pheatmap	R Core Team, 2016	https://cran.r-project.org/web/packages/pheatmap/index.html

LEAD CONTACT AND MATERIALS AVAILABILITY

Further information and requests for resources and reagents should be directed to and will be fulfilled by the Lead Contact, Dr. Ping Mu (ping.mu@utsouthwestern.edu). All cell lines, plasmids and other reagents generated in this study are available from the Lead Contact with a completed Materials Transfer Agreement if there is potential for commercial application.

EXPERIMENTAL MODEL AND SUBJECT DETAIL

SCID Mouse *In Vivo* Xenografts

All animal experiments were performed in compliance with the guidelines of the Animal Resource Center of UT Southwestern and Research Animal Resource Center of the MSKCC. LNCaP/AR *in vivo* xenograft experiments were conducted by subcutaneous injection of 2×10^6 LNCaP/AR cells (100 μl in 50% Matrigel, BD Biosciences, and 50% growth media) into the flanks of castrated male SCID mice on both sides. Daily gavage treatment with 10 mg/kg enzalutamide or vehicle (1% carboxymethyl cellulose, 0.1% Tween 80, 5% DMSO) was initiated one day after the injection. Once tumors were noticeable, tumor size was measured weekly by tumor measuring system Peira TM900 (Peira bvba, Belgium). For CWR22Pc *in vivo* experiments (Figures S3E and S3F), 2×10^6 CWR22Pc cells were injected subcutaneously into the flanks of intact male SCID mice and both castration and enzalutamide treatment (10 mg/kg) was initiated on day 27 of xenografting. For *in vivo* experiment in Figures S8F and S8G, 10 mg/kg enzalutamide and/or 60 mg/kg CPI-0610 were given after 5 weeks of enzalutamide alone administration, when tumors were around 200 mm^3 size in average. CPI-0610 and JQ1 are commercially available from Selleck Chemicals, details listed in Key Resources Table.

Human Prostate Cancer Cell Lines and Mouse Organoids

LNCaP/AR, CWR22Pc and LAPC4 prostate cancer cell lines were generated and maintained as previously described(Chen et al., 2003; Klein et al., 1997; Mu et al., 2017). E006AA cells were purchased from Millipore (Sigma-Aldrich) (#SCC102). LNCaP/AR, CWR22Pc and LAPC4 cells were cultured in RPMI medium supplemented with 10% fetal bovine serum (FBS), 1% L-glutamine, 1% penicillin-streptomycin, 1% HEPES, and 1% sodium pyruvate (denoted as normal culture medium). E006AA cells were cultured in DMEM medium supplemented with 10% fetal bovine serum (FBS), 1% L-glutamine, 1% penicillin-streptomycin, 1% HEPES, and 1% sodium pyruvate. LNCaP/AR cells were passaged every 3-5 days at a 1:6 ratio, CWR22Pc cells were passaged every 3-5 days at 1:3 ratio. LAPC4 cells were passed every 5-7 days at 1:2 ratio. E006AA cells were passaged every 3-5 days at 1:5 ratio. When treated with 10 μM enzalutamide LNCaP/AR cells were cultured in RPMI medium supplemented with 10% charcoal-stripped serum (denoted as CSS medium). All of the xenograft tumor-derived LNCaP/AR subsequent cell lines were developed from different individual tumors (treatment details as described in main text) that were harvested, disaggregated with collagenase treatments, and then maintained in normal culture medium. After harvesting, cells were cultured on Poly-D-Lysine-coated plates with 2 μg/ml puromycin (Gibco #A1113803) until confluence and were then maintained on standard tissue culture dishes. All cell cultures were assessed for mycoplasma monthly via the highly sensitive MycoAlert^{TM} PLUS Mycoplasma Detection kit from Lonza (Cat #LT07-710). Cell line identification was validated each year through the human STR profiling cell authentication provided by the UT Southwestern genomic sequencing core and compared to ATCC cell line profiles. *Pten*$^{-/-}$ mouse organoids were generated from Pb-Cre4-*Pten*$^{flox/flox}$ mice as previously described (Chen et al., 2013). This organoid (218-5A) is cultured in 3D Matrigel according to established protocol (Karthaus et al., 2014). This organoid is split at 1:3 ratio every 6 days by trypsin or sterile glass pipette.

shRNA and CRISPR Model Generation

Lentiviral or retroviral transduction of cells for shRNA or guide RNA experiments was performed as previously described with some modifications (Mu et al., 2017; Wheeler et al., 2015). Specifically, retroviral virus was used for shRNA library transduction in Figure 1, as well as shCHD1 KD in Figures 3E, 8A, 8C, 8D, and S2A–S2E. Lentiviral virus was used for CRISPR based KO in Figures 3A–3C, 6C, and S1C and inducible or stable shRNA constructs based KD in Figures S2F–S2H, S3, 6D, 8C, and 8D. For

the miR-E based shRNA library transduction, LNCaP/AR cells were transduced with pooled retroviral shRNA hairpins with a 5-20% transduction efficiency to ensure that most shRNAs are transduced at single-copy level. Two days after transduction, infected LNCaP/AR cells were selected with 2 μg/ml puromycin for four days to select a pure GFP positive population. Sequences of all the library shRNAs are listed in Table S2. For all other shRNA or CRISPR mediated modifications, unless otherwise noted, cells were seeded at 400,000 cells per well in 2 ml of media in 6-well plates. The next day, media was replaced with media containing 50% of virus and 50% of fresh culture medium, along with 5 μg/ml polybrene. The lentiviral or retroviral virus containing media was removed after 24 hours and replaced with regular culture medium. Three days post transduction, the cells were selected with 2 μg/ml puromycin for 4 days or 5 μg/ml blasticidin, as described below. The backbones and sequences of all the shRNAs and CRISPR guide RNAs are listed in the Method Details and Key Resources Table.

METHOD DETAILS

Generation of the Human PCa Deletome and Construction of the miR-E shRNA Library

To define a comprehensive human prostate cancer deletome, we developed an integrative pipeline to analyze the genomic copy number alterations (CNV) and mRNA expression data from multiple independent genomic studies. First, we examined the original CNV data of the 2010 Taylor dataset and filtered the list of deleted genes present in regions of recurrent focal and chromosome arm length deletion in more than 15% of the prostate cancer patients (generated 2 lists based on either the published CNV or the R.A.E. output) (Taylor et al., 2008; 2010). Then we integrated these CNV data and the corresponding gene expression data to further filtered the recurrent deletion events that are associated with decreased gene expression based on matched gene expression data. In parallel, we utilized this pipeline analysis for another three independent genomic studies and generated 4 additional deleted gene lists, including the 2012 Barbieri dataset, the 2012 Grasso dataset (2 lists based on either the published CNV or the R.A.E. output) and the TCGA dataset (Barbieri et al., 2012; Grasso et al., 2012; Network, 2015). Two more deleted gene lists were generated using similar approaches as our integrative pipeline and therefore added into our final 8 deleted gene lists, including the 2007 Kim dataset and the 2009 Holcomb dataset(Holcomb et al., 2009; Kim et al., 2007). As expected, these 8 deleted gene lists substantially overlap. We then combined the 8 deleted gene lists and refined the final human PCa deletome of 730 genes by only incorporating the genes whose deletion were confirmed by at least two independent studies (Table S1) (Two genes *PTEN* and *DACH1* were removed from the list because they were already deleted in LNCaP/AR cells) (Taylor et al., 2010). To identify genes whose protein product inhibition can confer resistance to antiandrogen therapy in prostate cancer, we built a custom shRNA library targeting 730 genes (5-6 shRNAs/gene, total 4234 shRNAs) (Table S2). The shRNAs were cloned in a LEPC (aka MLP-E) vector, a constitutive expression vector that was previously optimized for more efficient knockdown, by PCR-cloning a pool of oligonucleotides synthesized on 12k customized arrays (CustomArrays) as previously described (Zuber et al., 2010). The shRNAs were designed using an algorithm that predicts potent shRNAs as previously described (Pelossof et al., 2017). The library was subcloned into 43 independent pools each pool consisting of ~100 shRNAs, to ensure that shRNA representation was not lost after grafting the tumors cells *in vivo*.

In Vivo shRNA Mediated Screen and HiSeq

Each pool of the library was transduced into human CRPC tumor cell line LNCaP/AR at low multiplicity of infection (MOI < 1), to ensure a single retroviral integration per cell and achieving a representation of each shRNA in an average of 20,000 cells. Transduced LNCaP/AR cells were selected for 4 days using 2 μg/ml puromycin (Invitrogen) and 2 million cells were subcutaneously injected bilaterally into 5 castrated SCID mice to preserve library representation throughout the experiment (because of unexpected mice loss, we have added additional mice in several pools to get enough tumors). As a negative control group, LNCaP/AR cells transduced with shNT were also injected into 10 castrated mice. All animals were treated with enzalutamide (10 mg/kg/day) one day after the day of bilateral injection to mimic the clinical scenario of enzalutamide usage, with the exception of 5 mice in the negative control group being treated with vehicle. As described in the main text, based on the results of pilot experiments (Figures S1A and S1B), we only harvested the tumors that reached 100 mm^3 burden by week 16, before the appearance of background tumors (which usually require more than ~19 weeks to arise) based on the rationale that the shRNAs targeting candidate resistance biomarkers should confer resistance significantly quicker than the stochastic enrichment of the tumor initiating cells ("jackpot effect").

Genomic DNA from plasmids, pregrafts, and resistant tumors was isolated by two rounds of phenol extraction using Phase-Lock tubes (5prime) followed by isopropanol precipitation. The normalized reads of all shRNAs present in resistant tumors or starting materials were quantified using HiSeq 2500 sequencing of shRNA guide strands PCR amplified from the isolated genomic DNA, as previously described (Zuber et al., 2010; 2011). Sequence processing was performed using a customized Galaxy platform as previously described (Zuber et al., 2011). For each shRNA and condition, the number of matching reads was normalized to the total number of library-specific reads per lane (10 million total reads per pool) and used for further analysis. We only obtained 21 pools of reads in pregrafts therefore reads in plasmids were used as starting material instead. All the HiSeq sequencing results (FASTQ) and normalized reads files were deposited to GEO: GSE127957. To adapt a probabilistic ranking algorithm RIGER-E (RNAi Gene Enrichment Ranking) to analyze the HiSeq results, we recorded the hairpin reads in the tumors which did not score by week 16 as "0" because they failed to enrich quicker than stochastically enriched hairpins, in order to have a working matrix for a probabilistic statistic model. RIGER analysis was performed as previously described (Golden et al., 2017) and the data matrix was deposited to GEO: GSE127957. We then applied two additional cut-offs to further filter out the

false positive candidate genes. We chose "enriched in more than 8% of total tumor xenografted" as a first cut-off based on the stochastic enrichment ratio of negative control gene *TBC1D4*. We chose "4 out of 6 hairpins enriched" as the second cut-off based on a triangle thresholding method (Zack et al., 1977) and the results of our pilot experiments. The enrichment of each shRNAs was determined by comparing the normalized reads in the resistant tumors with the normalized reads in plasmids.

Individual Plasmid Construction and Virus Production

The retroviral (LEPG) and lentiviral (SGEP, LT3GEPIR) miR-E based expression vectors generous gifts from Dr. Johannes Zuber (Research Institute of Molecular Pathology, Vienna, Austria), and described previously(Zuber et al., 2011). LEPC, SCEP and LT3CEPIR vectors were constructed by switching the GFP cassette in the previous three vectors with a mCherry cassette as described previously (Mu et al., 2017).

The sequences of shRNA hairpins are listed below:

LEPG-shNT:

TGCTGTTGACAGTGAGCGCAGGAATTATAATGCTTATCTATAGTGAAGCCACAGATGTATAGATAAGCATTATAATTCCTATGCCTACTGCCTCGGA

LEPG-shCHD1-1:

TGCTGTTGACAGTGAGCGACAGGTTAACATTTTAGATAAATAGTGAAGCCACAGATGTATTTATCTAAAATGTTAACCTGGTGCCTACTGCCTCGGACTTCAAGGGGCTAGAATTC

LEPG-shCHD1-2:

TGCTGTTGACAGTGAGCGACAGGAAATGGATATAGATGAATAGTGAAGCCACAGATGTATTCATCTATATCCATTTCCTGGTGCCTACTGCCTCGGACTTCAAGGGGCTAGAATTC

LEPG-shCHD1-3:

TGCTGTTGACAGTGAGCGCAACGTTATATATGACAAATTATAGTGAAGCCACAGATGTATAATTTGTCATATATAACGTTTTGCCTACTGCCTCGGACTTCAAGGGGCTAGAATTC

LEPG-shCHD1-4:

TGCTGTTGACAGTGAGCGACAGGAGAGATTCAGTATTTAATAGTGAAGCCACAGATGTATTAAATACTGAATCTCTCCTGGTGCCTACTGCCTCGGACTTCAAGGGGCTAGAATTC

LEPG-shCHD1-5:

TGCTGTTGACAGTGAGCGCTAGGCGGTTTATCAAGAGCTATAGTGAAGCCACAGATGTATAGCTCTTGATAAACCGCCTAATGCCTACTGCCTCGGACTTCAAGGGGCTAGAATTC

LT3CEPIR-shGR-1:

TGCTGTTGACAGTGAGCGCCCAAAGCAGTTTCACTCTCAATAGTGAAGCCACAGATGTATTGAGAGTGAAACTGCTTTGGATGCCTACTGCCTCGGA

LT3CEPIR-shGR-2:

TGCTGTTGACAGTGAGCGAAAGCTGTAAAGTTTTCTTCAATAGTGAAGCCACAGATGTATTGAAGAAAACTTTACAGCTTCTGCCTACTGCCTCGGA

LT3CEPIR-shCHD1-1:

TGCTGTTGACAGTGAGCGACAGGTTAACATTTTAGATAAATAGTGAAGCCACAGATGTATTTATCTAAAATGTTAACCTGGTGCCTACTGCCTCGGACTTCAAGGGGCTAGAATTC

LT3CEPIR-shCHD1-2:

TGCTGTTGACAGTGAGCGACAGGAAATGGATATAGATGAATAGTGAAGCCACAGATGTATTCATCTATATCCATTTCCTGGTGCCTACTGCCTCGGACTTCAAGGGGCTAGAATTC

The All-In-One lentiCRISPR v2 purchased from Addgene (Plasmid #52961) was used to generate the sgCHD1, sgmChd1(for mouse organoid experiment) and sgPTEN constructs. The empty vector served as the sgNT control. The guide RNAs were designed using the online CRISPR designing tool at Benchling (https://benchling.com).

The sequences of sgRNAs are listed below:

lentiCRISPRv2-sgCHD1-1-F: CACCGTCAGCTCCATCAACTTTCGG
lentiCRISPRv2-sgCHD1-1-R: AAACCCGAAAGTTGATGGAGCTGAC
lentiCRISPRv2-sgCHD1-2-F: CACCGGATTTATGGATTGTCGGATT
lentiCRISPRv2-sgCHD1-2-R: AAACAATCCGACAATCCATAAATCC
lentiCRISPRv2-sgmChd1-1-F: CACCGAAAGTGTTAGAAATGGCAG
lentiCRISPRv2-sgmChd1-1-R: AAACCTGCCATTTCTAACACTTTC
lentiCRISPRv2-sgmChd1-2-F: CACCGCAACATTCACGGGTTTCCTG
lentiCRISPRv2-sgmChd1-2-R: AAACCAGGAAACCCGTGAATGTTGC
lentiCRISPRv2-sgPTEN-F: CACCGAAACAAAAGGAGATATCAAG
lentiCRISPRv2-sgPTEN-R: AAACCTTGATATCTCCTTTTGTTTC

All information related to constructs used for CRISPR function screening are discussed below in the functional screening section.

The *CHD1* expressing vectors pCDH-EF1-Chd1-T2A-copGFP and pCDH-EF1-Chd1-P2A-puro were generous gift from Dr. Ping Chi's laboratory at MSKCC.

FACS-Based Growth Competition Assay

LNCaP/AR cells were transduced with 5 different shRNAs targeting *CHD1* or shNT individually with a viral infection efficiency of ~20%, verified by GFP percentage by FACS. The competition cell mixture of ~20% transduced LNCaP/AR cells and ~80% wild-type cells was treated with 10 μM enzalutamide and the percentage of GFP positive cells were measured by FACS on day 0, day 6, day 12, day 17 and day 24. Relative cell number fold change was calculated as follows:
$\frac{T2\times Y}{T1\times X} \div \frac{T2\times(1-Y)}{T1\times(1-X)} = \frac{Y\times(1-X)}{X\times(1-Y)}$, where T1 is the total cell number of cell mixture on day 0 and T2 is the total cell number on day 6, 12,17, or 24; X is the percentage of GFP positive cells measured on day 0 and Y is the percentage of GFP positive cells measured on day 6,12,17, or 24; then 1-X is the percentage of wild-type uninfected cells on day 0 and 1-Y is the percentage of wild-type uninfected cells on day 6, 12, 17 or 24. FACS-based competition assay in Figures 8C and S8D is analogous to the one in Figures 3D and 3E described above, except the shCHD1-XE-1 cells transduced with LT3CEPIR-shGRs were treated with doxycycline for 48 hours at 250 ng/ml before the day 0 was measured.

Cell Growth Assay, Cell Viability Assays and Dose Response Curve

LNCaP/AR cells transduced with CRISPR/sgRNAs were seeded at 20,000 cells per well in a 24-well cell culture plate, in CSS medium and treated with enzalutamide (10 μM) or vehicle (DMSO) for 6 days. Cell numbers were counted using a Countess II FL automatic cell counter (Invitrogen) on day 7 and the relative cell growth (Enz/DMSO) was calculated. Cell growth assays were conducted in triplicate and mean ± SEM were reported. Dose response curve and all other cell viability assays were measured by CellTiter-Glo luminescent cell viability assay (Promega cat #7570). 4000 LNCaP/AR cells were seeded in 96-well dish and treated with different dosages of enzalutamide for 3 days before performing the assay. 3000 CWR22Pc cells were seeded in 96-well plate and treated with different dosages of enzalutamide for 6 days before performing the assay. 5000 LAPC4 cells were seeded in 96-well plate with different dosages of enzalutamide for 12 days before perform the assay. 500 E006AA cells were seeded in 3D Matrigel in human organoid media (Gao et al., 2014; Karthaus et al., 2014) with enzalutamide for 6 days, because E006AA cells are not very sensitive to enzalutamide treatment in 2D culture condition. Mouse organoid were seeded in 3D Matrigel (1000 cells/per 50 μl sphere) in mouse organoid media (Karthaus et al., 2014) with 1 μM enzalutamide for 6 days before the cell viability was read.

Gene Expression Assay by qPCR

Total RNA from cells or homogenized tissues was extracted using Trizol (Ambion, Cat 15596018) following manufacturer's instructions. cDNA was made using the SuperScript™ IV VILO™ Master Mix with ezDNase™ Enzyme (Thermo Fisher, 11766500) following manufacturer's instructions, with 200 ng/μl RNA template. 2X PowerUp™ SYBR™ Green Master Mix (Thermo Fisher, A25778) was used in the amplification of the cDNA. Assays were performed in triplicate and normalized to endogenous β-Actin expression. Heatmaps represent the gene expression difference were generated by prism 8, using the log10 of expression fold change compared to control cell lines (shNT or sgNT transduced LNCaP/AR). Qiagen RT2 qPCR primer assays are used as primers for gene expression detection, unless otherwise noted. Individual primer assays are listed, as well as in Table S6.

AR, Qiagen RT2, Cat# PPH01016A
KLK3, Qiagen RT2, Cat# PPH01002B
NKX3-1, Qiagen RT2, Cat# PPH02267C
TMPRSS2, Qiagen RT2, Cat# PPH02262C
NDRG1, Qiagen RT2, Cat# PPH02202B
NR3C1 (GR), Qiagen RT2, Cat# PPH02652A
TBX2, Origene, F-AGCAGTGGATGGCTAAGCCTGT
R-GGATGTCGTTGGCTCGCACTAT
NR2F1, Origene, F-TGCCTCAAAGCCATCGTGCTGT
R-CAGCAGCAGTTTGCCAAAACGG
POU3F2, Origene, F-GTGTTCTCGCAGACCACCATCT
R-GCTGCGATCTTGTCTATGCTCG
SGK1, Qiagen RT2, Cat# PPH00387F
NPC1, Sigma KiCqStart, Cat#H_NPC1_1, 4864
KRT8, Qiagen RT2, Cat# PPH02214F
KRT18, Qiagen RT2, Cat# PPH00452F
KRT5, Qiagen RT2, Cat# PPH02625F
KRT14, Qiagen RT2, Cat# PPH02389A
TP63, Qiagen RT2, Cat# PPH01032F
SYP, Qiagen RT2, Cat# PPH00717A
CHGA, Qiagen RT2, Cat# PPH01181A
ENO2, Qiagen RT2, Cat# PPH02058A
SOX2, Qiagen RT2, Cat# PPH02471A

SNAI2, Qiagen RT2, Cat# PPH02475A
TWIST1, Qiagen RT2, Cat# PPH02132A
SNAI1, Qiagen RT2, Cat# PPH02459B
ZEB1, Qiagen RT2, Cat# PPH01922A
CDH2, Qiagen RT2, Cat# PPH00636F

Western Blot

Proteins were extracted from whole cell lysate using RIPA buffer. Proteins were then measured with Pierce BCA Protein Assay Kit (cat #23225) following manufacturer's instructions. Protein lyses were mixed with 5X laemmli buffer and boiled at 95°C for 5 minutes. Proteins were run on the NuPAGE 4-12% Bis-Tris gels (Invitrogen, Cat #NP0323) using Novex sharp pre-stained protein standards as a marker (Invitrogen, LC8500) and 1X NuPAGE MES SDS buffer as running buffer (Novex, Cat #NP0002) and run at 120 volts. Gels were transferred in 1X Bolt Transfer buffer (Novex, Cat #BT00061) diluted with water and ethanol. Nitrocellulose membrane paper (Immobilon, Cat#IPVH00010) was used and was activated with 100% methanol (Fisher, Cat#A412-20). Transfer was conducted at 4°C for 1 hour at 100 volts. Membranes were blocked in 5% non-fat milk for 15 minutes prior to addition of primary antibody and washed with 1X TBST (10X stock from Teknova, T9511).

Antibodies used for western blot are (also listed in Key Resources Table):

(1) CHD1 (D8C2) Rabbit mAb, Cell Signaling, Cat #4351
(2) AR Antibody (N-20), Santa Cruz, sc-816
(3) KLK3 (D6B1) XP® Rabbit mAb, Cell Signaling, Cat # 5365
(4) PMEPA1 Antibody (P-15), Santa Cruz, Cat # sc-85829
(5) STEAP Antibody (B-4), Santa Cruz, Cat # sc-271872
(6) β-Actin (13E5) Rabbit mAb, Cell Signaling, Cat # 4970
(7) Glucocorticoid Receptor (D6H2L) XP® Rabbit mAb, Cell Signaling, Cat #12041
(8) SGK1 (D27C11) Rabbit mAb, Cell Signaling, Cat #12103
(9) c-Myc (D84C12) Rabbit mAb, Cell Signaling, Cat #5605

Immunofluorescence (IF)

LNCaP/AR cells were seeded on round glass coverslips. After 24 hr, cells were washed with PBS and fixed with 4% paraformaldehyde for 20 min at room temperature, followed by permeabilization with 0.5% Triton X-100 for 5 min. Then cells were incubated with primary antibodies (Rabbit anti-GR, CST, #12041; mouse anti-NR2F1 R&D, PP-H8132-00), overnight at 4°C after blocking with 3% BSA/PBS for 30 min at room temperature, followed by incubation with Alexa Fluor-labeled secondary antibodies (Alexa Fluor® 488 AffiniPure Goat Anti-Mouse IgG (H+L), Jackson Immunoresearch; Alexa Fluor® 594 AffiniPure Goat Anti-Rabbit IgG (H+L), Jackson Immunoresearch) for 1hr at room temperature. Nuclei were stained with DAPI. Images were acquired on Leica DMi8 microscope and Zeiss LSM 700 confocal Laser Scanning Microscope. Three biological replicated, representative images of each cell line were used to quantify the fluorescence intensity of GR and NR2F1 signals, using imageJ.

Immunohistochemistry (IHC)

Tumors were fixed in 4% paraformaldehyde overnight at 4°C. Then tumors were embedded in paraffin and sectioned at 5 μm. Immunohistochemistry was performed following standard procedures. After incubated with primary antibodies (Rabbit anti-GR, CST, #12041; mouse anti-NR2F1 R&D, PP-H8132-00), VECTASTAIN® ABC HRP Kit (Peroxidase, Rabbit IgG) and HRP conjugated Goat anti-mouse IgG were used, followed by ImmPACT® DAB Peroxidase (HRP) Substrate. Images were acquired on ECHO revolve microscope. Representative images of four tumors of each group were used to quantify the IHC signals of GR and NR2F1, using imageJ and the IHC Profiler plugin (Varghese et al., 2014).

FACS-based Functional Screen Mediated by CRISPR/Cas9

LNCaP/AR-shCHD1 cells (GFP positive) were transduced with lentiCas9-Blast purchased from Addgene (Plasmid #52962) and then selected with 5 μg/ml blasticidin (Gibco #A1113903) for 5 days. Four individual guide RNAs were designed to target each of the top 22 candidate TFs using the online CRISPR designing tool at Benchling (https://benchling.com). The sequences of sgRNAs can be found in Table S5. These guide RNAs were individually cloned into pLKO5.sgRNA.EFS.tRFP purchased from Addgene (Plasmid #57823). Then the LNCaP/AR-shCHD1-Cas9-Blast cells were transduced with these guide RNAs (guide RNAs targeting the same TF were pooled together) or sgNT with a viral infection efficiency of 50-90%, as measured by percentage of RFP positive cells (achieving a cell mixture of RFP positive cells vs RFP negative cells). The transduced cells were treated with 10 μM enzalutamide and the percentage of RFP positive cells were measured by FACS on day 0 and day 7. If deletion of any TF by CRISPR/Cas9 compromised the resistance to enzalutamide, it will give the infected cells with a growth disadvantage that will in turn be reflected by a reduction in the percentage of RFP positive cells.

QUANTIFICATION AND STATISTICAL ANALYSIS

Statistics Methods

All of the statistical details of experiments can be found in figure legends as well as the Method Details section. For all comparisons between two groups of independent datasets, multiple t tests were performed, p value and standard error of the mean (SEM) were reported. For all comparisons among more than two groups (>2), one-way or two-way ANOVA were performed, p values and SEM were reported; and p values were adjusted by multiple testing corrections (Bonferroni) when applicable. For dose response curve, p values were calculated by non-linear regression with extra sum-of-squares F test. For all figures, **** represents p<0.0001. *** represents p<0.001. ** represents p<0.01. * represents p<0.05. The usage of all statistical approaches was examined by our biostatistical collaborators. All bioinformatic analysis and comparisons are described in details below.

Analysis of Human Prostate Cancer Dataset

Processed 444 SU2C metastatic prostate cancer patient cohort (Abida et al., 2019) RNA-seq data and enzalutamide/abiraterone treatment data were downloaded from cBioPortal (http://www.cbioportal.org/). 128 patients of this cohort with metastatic CRPC have baseline biopsy and matched clinical data. 75 patients of this 128 sub-cohort have gene expression data captured by Poly-A RNA-seq. 56 patients of this 75 sub-cohort have records of time on either enzalutamide/apalutamide or abiraterone. 4 patients of this cohort were excluded because they have *SPOP* mutations, which demonstrate elevated sensitivity to antiandrogen treatment (Boysen et al., 2018). Histogram of *CHD1* mRNA distribution was generated by R Studio (Version 1.1.453). The probability of treatment duration figure was generated by prism 8 using Mantel-Cox test.

The same SU2C cohort (Abida et al., 2019) RNA-seq data was used to analyze expression patterns of 4 TFs (*NR3C1*, *POU3F2*, *NR2F1* and *TBX2*) and their relationship with *CHD1* level. Among these patients, RNA-seq data (Capture platform) for all 5 genes were available for 212 patients. We excluded patients with only polyA RNA-seq data because *NR2F1* expression is not available from the polyA platform. Expression matrix of all 5 genes was analyzed by "hclust" method (Müllner, 2013), with the parameter k-means= 5, scale = "column" (normalized value centered by gene). Unsupervised clustering resulted in 5 distinct groups, using the "pheatmap" package of R (V1.0.12). Each cluster contains different number of tumors (Cluster:1 Size:102, Cluster:2 Size:27, Cluster3: Size:2, Cluster:4 Size:4, Cluster:5, Size:40).

Sample and library preparation for RNA-seq and ATAC-seq

$1x10^6$ LNCaP/AR cells was plated in 6-well plate, growing under regular RPMI-1640 containing 10% FBS. After 48 hours, cells were trypsinized and collected by spinning at 500 g for 1.5 min, 4° C. Cells were then washed once with cold 1X PBS and spinned down at 500 g for 1.5 min, 4° C. After discarding supernatant, cells were lysed using 50 μL cold lysis buffer (10 mM Tris-HCl pH 7.4, 10 mM NaCl, 3 mM $MgCl_2$, 0.1% IGEPAL CA-360) and spinned down immediately at 500 g for 10 min, 4 ° C. Total RNA from cells was extracted using Trizol (Ambion, Cat 15596018) following manufacturer's instructions. RNA-Seq libraries were prepared using the Illumina TruSeq stranded mRNA kit, with 10 cycles of PCR amplification, starting from 500 ng of total RNA, at the Genome Technology Center (GTC) at NYU. Barcoded RNA-Seq were run as single read 50 nucleotides in length on the Illumina HiSeq 2500 (v4 chemistry) and Poly-A selection was performed. For ATAC-seq, $5x10^5$ LNCaP/AR cells were precipitated and kept on ice and subsequently resuspended in 25 μL 2X TD Buffer (Illumina Nextera kit), 2.5 μL Transposase enzyme (Illumina Nextera kit, 15028252) and 22.5 μL Nuclease-free water in a total of 50 μL reaction for 1 hr at 37° C. DNA was then purified using Qiagen MinElute PCR purification kit (28004) in a final volume of 10 μL. ATAC-Seq- Libraries were prepared following the Buenrostro protocol (https://www.ncbi.nlm.nih.gov/pmc/articles/PMC4374986/) and ATAC-Seq libraries were sequenced as 50 base paired-end reads on the Illumina HiSeq 4000 at the Genome Technology Center (GTC) at NYU.

Analysis of RNA-seq Data

Reads with Phred quality scores less than 20 and less than 35 bp after trimming were removed from further analysis using trimgalore (v0.4.1) (Martin, 2011). Quality-filtered reads were then aligned to the human reference genome GRCh38 using the HISAT (v2.0.1) (Pertea et al., 2016) aligner with default settings and marked duplicates using Sambamba (v0.6.6) (Tarasov et al., 2015). Aligned reads were quantified using featureCounts (v1.4.6) (Liao et al., 2014) per gene ID against GENCODE v10 GRCh38.p10 (Mudge and Harrow, 2015). Differential gene expression analysis was performed using the R package DEseq2 (v1.6.3) (Love et al., 2014). Cutoff values of absolute fold change greater than 2 and FDR<0.1 were used to select for differentially expressed genes between sample group comparisons. All RNA-seq data have been deposited in the Sequence Read Archive (SRA) with the accession numbers GSE126917, also listed in Key Resources Table.

GO Analysis

GeneOntology Enrichment Pathway analysis was performed using PANTHER to determine molecular and biological functional categories which were enriched in CHD1-depelted cells (Mu et al., 2017). The input gene lists were generated from the overlapping of differentially expressed genes in four compilations (shCHD1-1 compared to shNT, shCHD1-2 compared to shNT, shCHD1-XE-1 compared to shNT, shCHD1-XE-2 compared to shNT), which consistence of 150 genes in total. Cutoff values of FDR<0.05 was used to select top enriched pathways. To avoid pathways with too few genes, we excluded the gene lists with less than 10 hits changed in our datasets.

GSEA Analysis

GSEA statistical analysis was carried out with publicly available software from the Broad Institute (http://www.broadinstitute.org/gsea/index.jsp). Weighted GSEA enrichment statistic and Signal2Noise metric for ranking genes were used. The AR selective gene score was calculated by the sum of RPKM of all genes in the AR selective gene list as previously defined (Arora et al., 2013).

Analysis of ATAC-seq Data

We utilized trimgalore (v0.4.1) (Martin, 2011) for the raw reads to remove reads shorter than 35 bp or with Phred quality scores less than 20 and then aligned those trimmed reads to the human reference genome (GRCh38) using default parameters in BWA (v0.7.12) (Li and Durbin, 2009). The aligned reads were subsequently filtered for quality and uniquely mappable reads were retained for further analysis using Samtools (v1.3) (Li et al., 2009) and Sambamba (v0.6.6) (Tarasov et al., 2015). Library complexity was measured using BEDTools (v2.26.0) (Quinlan and Hall, 2010) and meets ENCODE data quality standards (Landt et al., 2012). Relaxed peaks were called using MACS (v2.1.0) (Feng et al., 2012) with a p value of $1x10^{-2}$. Consensus peaks were calculated by taking the overlap of peaks for sample, its replicates, and pseudoreplicates. All ATAC-seq data have been deposited in the Sequence Read Archive (SRA) with the accession numbers GSE127241, also listed in Key Resources Table.

Differential Binding Analysis

To detect differentially bound sites, we used R (v3.3.2) and package DiffBind (v2.2.12) (Stark and Brown, 2011; R Core Team, 2016) Default parameters were used in DiffBind workflow. To identify overlapping peaks between conditions we used BEDtools (v2.26.0), using intersect (Quinlan and Hall, 2010).

ATAC-seq Differential Peak and RNA-Seq Fold Change CDF Plots

We filtered the above annotated differential peak data for peak locations having fold changes of greater than 2 and greater than 5 separately, with associated p values of 0.01 or less. We then took the gene name from these filtered peak annotations and plotted the cumulative distribution of the gene's RNA-seq differential expression log2 fold change values against the cumulative distribution of the log2 expression fold change of all genes.

Annotation and Differential Motif Detection

To identify motif presence in peaks, we created a list of possible binding sites across the human reference (GRCh38) genome of motifs obtained from the JASPAR 2018 core vertebrate non-redundant database using the *fimo* command from the MEME suite (v4.11.1) (Bailey et al., 2015; Khan et al., 2017). We then performed differential motif analysis using DAStk (v0.1.5) on ATAC-seq peaks (Tripodi et al., 2018). ATAC-seq peaks were annotated using the annotatePeaks. script in HOMER (v4.9) (Heinz et al., 2010).

Predicting Driver TFs Using RNA-seq and ATAC-seq Data

We developed a workflow (Barnes et al., 2019) that combines RNA-seq and ATAC-seq data with TF motif information to predict driver TFs in prostate cancer resistance, similar as the method as previously described (Franco et al., 2018).

Transcription Factor Expression using RNA-seq: For each cell line (2 shCHD1-XE lines and two shCHD1 lines) we calculated the RNA-Score as the RNA-seq log2 fold change values compared to shNT cells.

Motif Predictions using ATAC-seq: For each cell line (2 shCHD1-XE lines and two shCHD1 lines) we calculated the ATAC-seq from the DAStk derived motif differential scores.

Determining driver Transcription Factors: To avoid having results from one of the four cell lines dominate the entire analysis, a weight γ was first calculated for each group by dividing the sum of the absolute value shCHD1-XE RNA-seq fold change values by the sum of the absolute value of shCHD1 RNA-Scores. A γ was also calculated for ATAC-Scores by dividing the sum of shCHD1-XE motif differential scores by the sum of shCHD1 ATAC-Scores, as shown in this equation: $\gamma = \frac{\sum |\text{shCHD1}-\text{XEs}|}{\sum |\text{shCHD1s}|}$. RNA fold change values and motif differential Scores were then multiplied by the respective weights, and then summed to create overall RNA-Scores and ATAC-Scores, respectively (Table S5). Then a Combined-Score is calculated by multiplying the overall RNA-Score and ATAC-Score.

If the TF has both negative value of RNA-Score and ATAC-Score, the Combined-Score was multiplied by -1 to get the adjusted Combined-Score. Furthermore, because some TFs may upregulate the downstream signaling pathway without significant changes in chromatin accessibility, or upregulate the downstream signaling pathway with only changes in chromatin accessibility, the Combined-Score of TFs with top 12 RNA-Scores and/or top 5 ATAC-Scores was also multiplied by -1 if it was a negative value (cut-off was picked based on the previously known function of these TFs). Then all the TFs are ranked using the adjusted Combined-Score (Figure 5H). The top 20 TFs with highest adjusted Combined-Score plus the 2 TFs with highest ATAC-Score are selected as final candidate resistant drivers for further functional screen.

Generating Density Heatmaps and Profiles

For heatmaps and profiles, we used deepTools (v2.5.0) (Ramírez et al., 2016) to generate read abundance from all datasets around peak center (± 2.5 kb/ 2.0 kb), using 'computeMatrix'. These matrices were then used to create heatmaps and profiles, using deepTools commands 'plotHeatmap' or 'plotProfile' respectively.

DATA AND CODE AVAILABILITY

Library shRNA HiSeq data has been deposited in GEO: GSE127957. RNA-Seq data has been deposited in GEO: GSE126917. ATAC-Seq data has been deposited in GEO: GSE127241.

Cancer Cell, Volume *37*

Supplemental Information

Loss of *CHD1* Promotes Heterogeneous Mechanisms of Resistance to AR-Targeted Therapy via Chromatin Dysregulation

Zeda Zhang, Chuanli Zhou, Xiaoling Li, Spencer D. Barnes, Su Deng, Elizabeth Hoover, Chi-Chao Chen, Young Sun Lee, Yanxiao Zhang, Choushi Wang, Lauren A. Metang, Chao Wu, Carla Rodriguez Tirado, Nickolas A. Johnson, John Wongvipat, Kristina Navrazhina, Zhen Cao, Danielle Choi, Chun-Hao Huang, Eliot Linton, Xiaoping Chen, Yupu Liang, Christopher E. Mason, Elisa de Stanchina, Wassim Abida, Amaia Lujambio, Sheng Li, Scott W. Lowe, Joshua T. Mendell, Venkat S. Malladi, Charles L. Sawyers, and Ping Mu

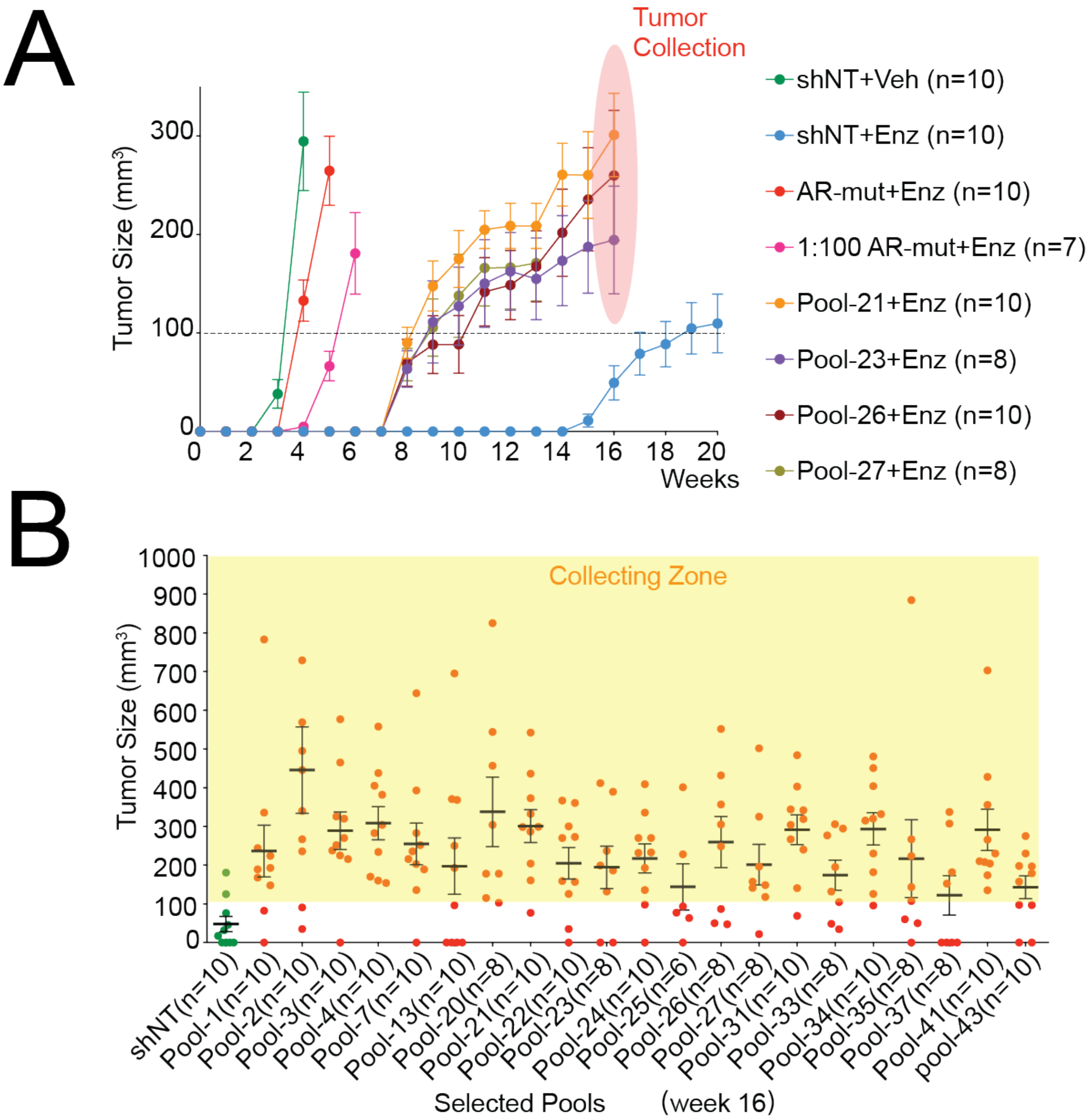

Figure S1 related to Figure 1. Pilot experiments and the validation of other two top hits emerged from the *in vivo* library screen. (A) Tumor growth curve of xenografted LNCaP/AR cells transduced with annotated plasmids or pools of library. Enz denotes enzalutamide treatment at 10 mg/kg orally one day after grafting. Veh denotes 0.5% CMC + 0.1% Tween 80 treatment at same dosage. (B) Tumor measurement of xenografted LNCaP/AR cells transduced with shNT and 21 representative pools at week 16. For all panels unless otherwise noted, mean ± SEM. is represented.

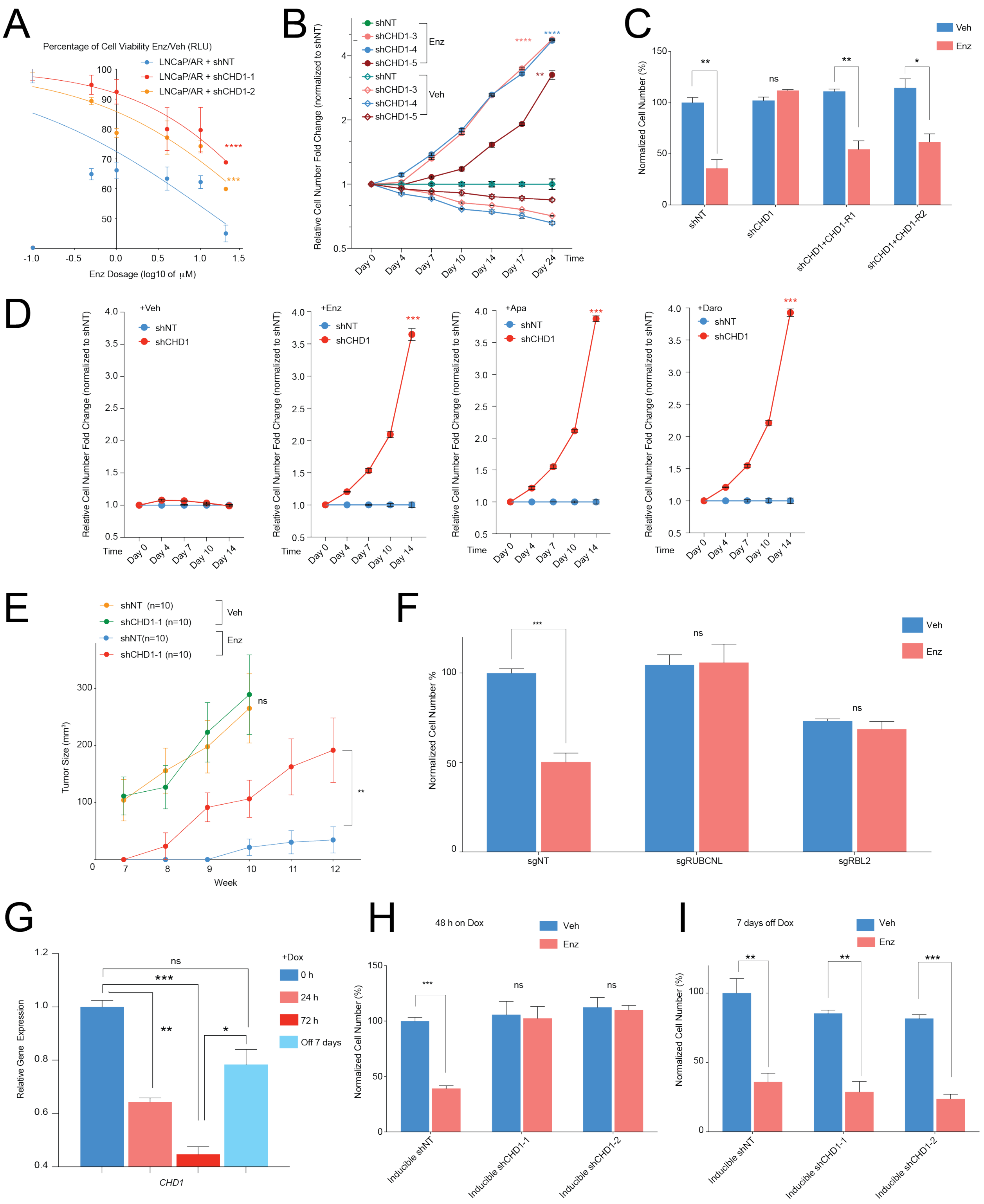

Figure S2 related to Figure 3. Resistance conferred by *CHD1* KD is reversible. (A) Dose response curve of LNCaP/AR cells transduced with annotated shRNAs. Mean ± SEM is represented, and p values were calculated by non-linear regression with extra sun-of-squares F test, 3 biological replicates were used for each data point. (B) Relative cell number fold change compared to shNT group, based on the results of FACS-based competition assay. Enz denotes

enzalutamide of 10 μM and Veh denotes DMSO. Mean ± SEM is represented, and p values were calculated using two-way ANOVA, 3 biological replicates in each group. (C) Relative cell number of LNCaP/AR cells transduced with annotated shRNAs and rescue plasmids expressing CHD1 cDNA (R1/R2), normalized to shNT+Veh group. Cells were treated with 10 μM enzalutamide (Enz) or DMSO (Veh) for 7 days and cell numbers were counted. Mean ± SEM is represented, and p values were calculated using multiple t tests, 3 biological replicates in each group. (D) Relative cell number fold change compared to shNT group, based on the results of competition assay. Veh denotes DMSO. Enzalutamide (Enz), apalutamide (Apa) and darolutamide (Daro) all denotes dosage of 10 μM. Mean ± SEM is represented, and p values were calculated using multiple t tests, 3 biological replicates in each group. (E) Tumor growth curve of xenografted LNCaP/AR cells transduced with annotated shRNAs. Enz denotes 10 mg/kg orally one day after grafting. Veh denotes 0.5% CMC + 0.1% Tween 80 at same dosage. Mean ± SEM is represented, and p values were calculated using two-way ANOVA. Experiments have been done with two independent repeats. (F) Relative cell number of LNCaP/AR cells transduced with annotated guide RNAs, normalized to shNT+Veh group. Cells were treated with 10 μM enzalutamide (Enz) or DMSO (Veh) for 7 days and cell numbers were counted. Mean ± SEM is represented, and p values were calculated using multiple t tests, 3 biological replicates in each group. (G) Relative gene expression level of *CHD1* in LNCaP/AR cells transduced with annotated inducible shRNAs at various time points. Mean ± SEM is represented, p values were calculated by one-way ANOVA, compared to 0 hr condition, 3 technical replicates in each group. (H) Relative cell number of LNCaP/AR cells transduced with annotated shRNAs in an inducible vector system, normalized to shNT+Veh. Cells were treated with 250 ng/ml doxycycline (Dox) for 48 hours, and then treated with 7 days of 10 μM enzalutamide (Enz) or DMSO (Veh), and cell numbers were counted. Mean ± SEM is represented, and p values were calculated using multiple t tests, 3 biological replicates in each group. (I) Relative cell number of LNCaP/AR cells transduced with annotated shRNAs in an inducible vector system, normalized to shNT+Veh. Cells were treated with 250 ng/ml doxycycline (Dox) for 48 hours, removed doxycycline for 7 days, and then treated with 7 days of 10 μM enzalutamide (Enz) or DMSO (Veh), then cell numbers were counted. Mean ± SEM is represented, and p values were calculated using multiple t tests, 3 biological replicates in each group. For all panels, **** $p<0.0001$. *** $p<0.001$. ** $p<0.01$. * $p<0.05$.

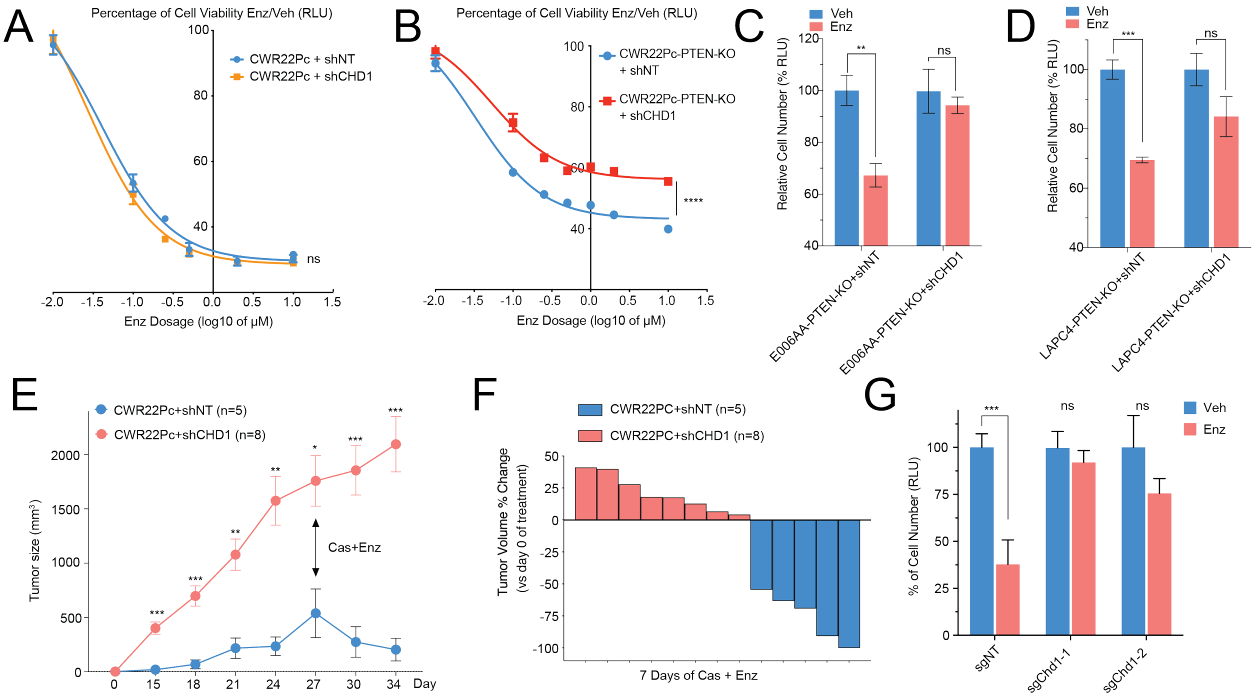

Figure S3 related to Figure 3. *CHD1* KD confers resistance in other PCa models. (A) Enzalutamide (Enz) dose response curve of CWR22Pc cells transduced with annotated shRNAs. Mean ± SEM is represented, and p values were calculated by non-linear regression with extra sun-of-squares F test, 3 biological replicates were used for each data point. (B) Enzalutamide (Enz) response curve of CWR22Pc-PTEN-KO cells transduced with annotated shRNAs. Mean ± SEM is represented, and p values were calculated by non-linear regression with extra sun-of-squares F test, 3 biological replicates were used for each data point. (C) Relative cell number of E006AA-PTEN-KO cells transduced with annotated hairpins. Cells were treated with 15 µg/ml enzalutamide (Enz) or DMSO (Veh) for 6 days in 3D Matrigel and cell number was measured using CellTiter-Glo assay. Mean ± SEM is represented, and p values were calculated using multiple t tests, 3 biological replicates in each group. (D) Relative cell number of LAPC4-PTEN-KO cells transduced with annotated hairpins. Cells were treated with 30 µg/ml enzalutamide (Enz) or DMSO (Veh) for 6 days and cell number was measured using CellTiter-Glo assay. Mean ± SEM is represented, and p values were calculated using multiple t tests, 3 biological replicates in each group. (E) Tumor growth curve of xenografted CWR22Pc cells in intact mice. All animals were castrated (Cas) and treated with enzalutamide (Enz) at 10 mg/kg orally from day 27. Mean ± SEM is represented and p values were calculated using two-way ANOVA. (F) Waterfall plot displaying changes in tumor size of xenografted CWR22Pc cells after 1 week of castration and enzalutamide treatments. Cas denotes castration. Enz denotes enzalutamide treatment at 10 mg/kg orally. (G) Relative cell number of mouse organoid (*Pten*$^{-/-}$) cultured in 3D. Organoids were treated with DMSO (Veh) or 1 µM enzalutamide (Enz) for 6 days. Mean ± SEM is represented, and p values were calculated using multiple t tests, 3 biological replicates in each group. For all panels, **** p<0.0001. *** p<0.001. ** p<0.01. * p<0.05.

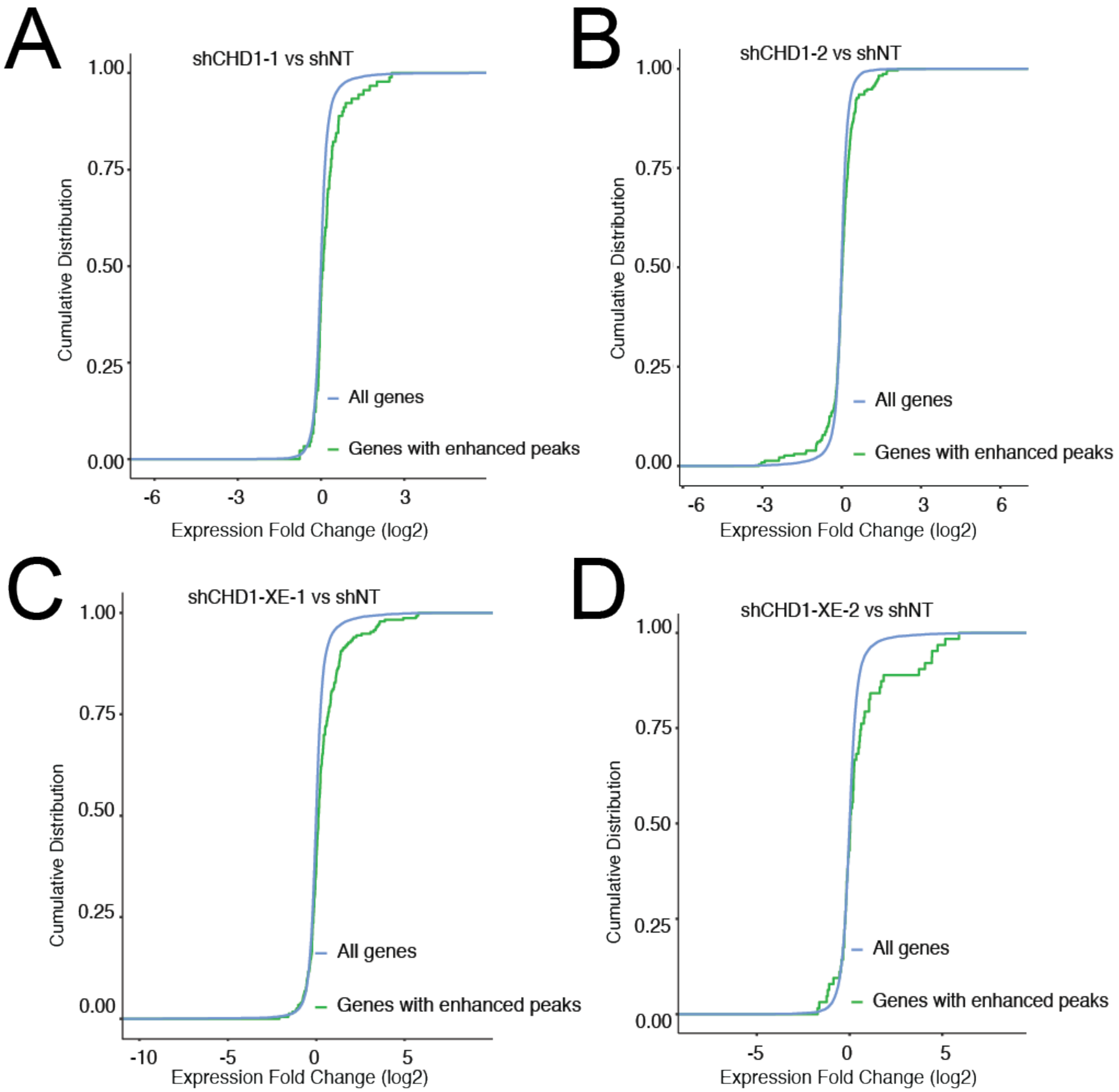

Figure S4 related to Figure 5. ATAC-seq analysis reveals global changes in chromatin accessibility after *CHD1* loss. (A-D) Cumulative distribution of log2 expression changes in shCHD1-1 cell line compared to shNT(A); shCHD1-2 cell line compared to shNT (B); shCHD1-XE-1 cell line compared to shNT (C); shCHD1-XE-2 cell line compared to shNT (D). For all panels, the blue line denotes all of the expressed genes. Green line denotes the genes with significant upregulated ATAC-peaks compared to shNT (enhanced peaks, combined increasing of peaks>6). For all panels, reads from 3 biological replicates were pooled to calculated the consensus peaks.

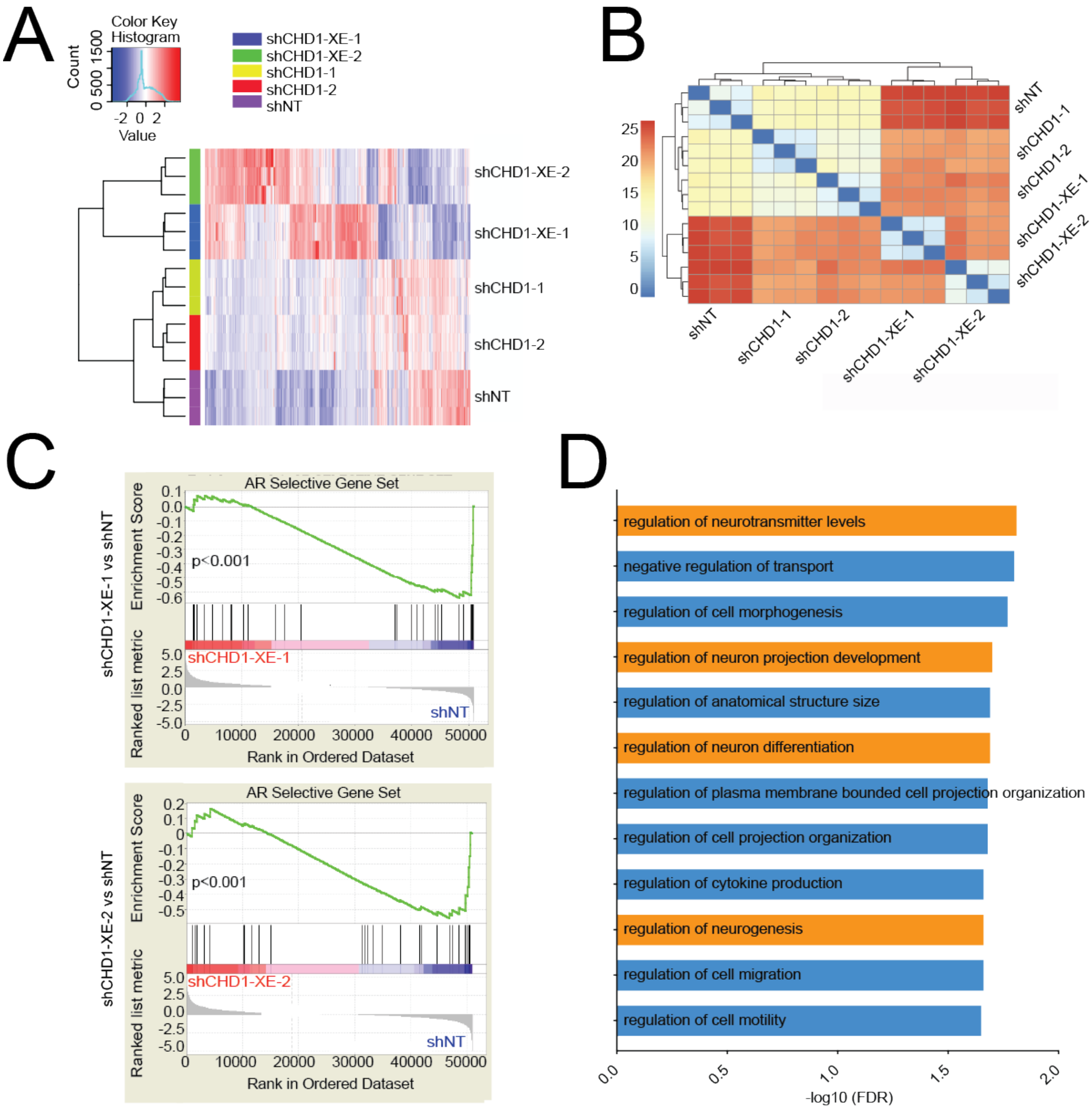

Figure S5 related to Figure 5. RNA-seq analysis reveals global changes in transcriptome profiling after *CHD1* loss. (A) Unsupervised hierarchical clustering of normalized expression of differentially expressed genes whose expression was significantly changed in any of the four other cell lines (shCHD1-1, shCHD1-2, shCHD1-XE-1, shCHD1-XE-2) comparing to shNT. 3 biological replicates in each group are shown. (B) Heatmap depicting the Euclidean distances between samples based on Pearson correlation. Reads from 3 biological replicates in each group were used for analysis. (C) GSEA analysis of AR selected genes (Arora et al. 2013) expression in shCHD1-XE groups compared to shNT group. Reads from 3 biological replicates were used for analysis. (D) Pathways enriched in the overlapped 150 significantly upregulated genes (see also Figure 5E) in the four cell groups compared to shNT. Reads from 3 biological replicates in each group were used for analysis.

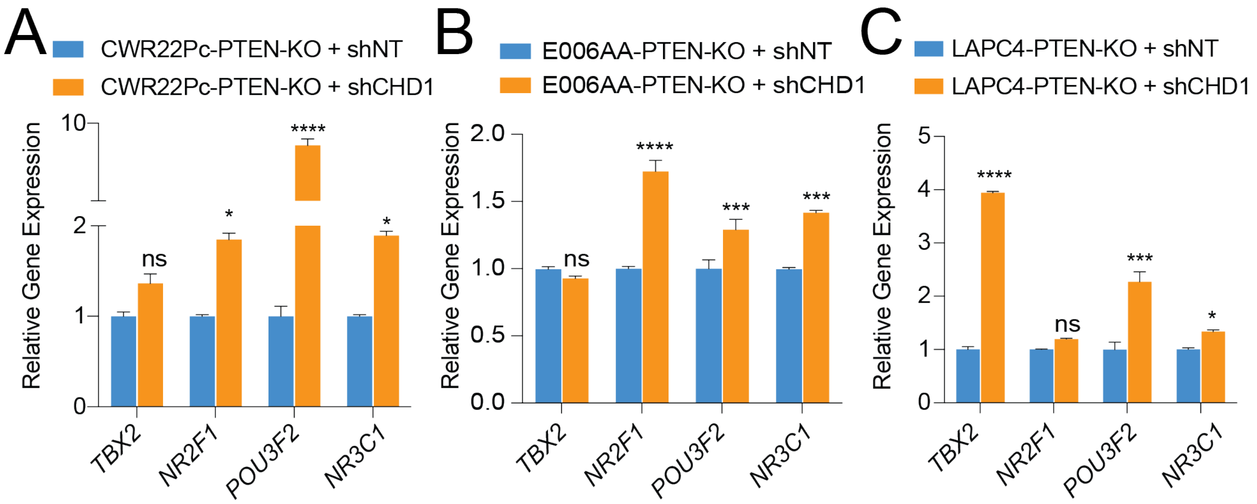

Figure S6 related to Figure 6. *CHD1* loss leads to induction of 4 TFs in other PCa models. (A) Relative gene expression of 4 TFs in CWR22Pc-PTEN-KO cells transduced with annotated shRNAs, all normalized and compared to shNT. (B) Relative gene expression of 4 TFs in E006AA-PTEN-KO cells transduced with annotated shRNAs, all normalized and compared to shNT. (C) Relative gene expression of 4 TFs in LAPC4-PTEN-KO cells transduced with annotated shRNAs, all normalized and compared to shNT. For all panels, mean ± SEM is represented and p values were calculated by multiple t test, 3 technical replicates in each group and **** p<0.0001. *** p<0.001. ** p<0.01. * p<0.05.

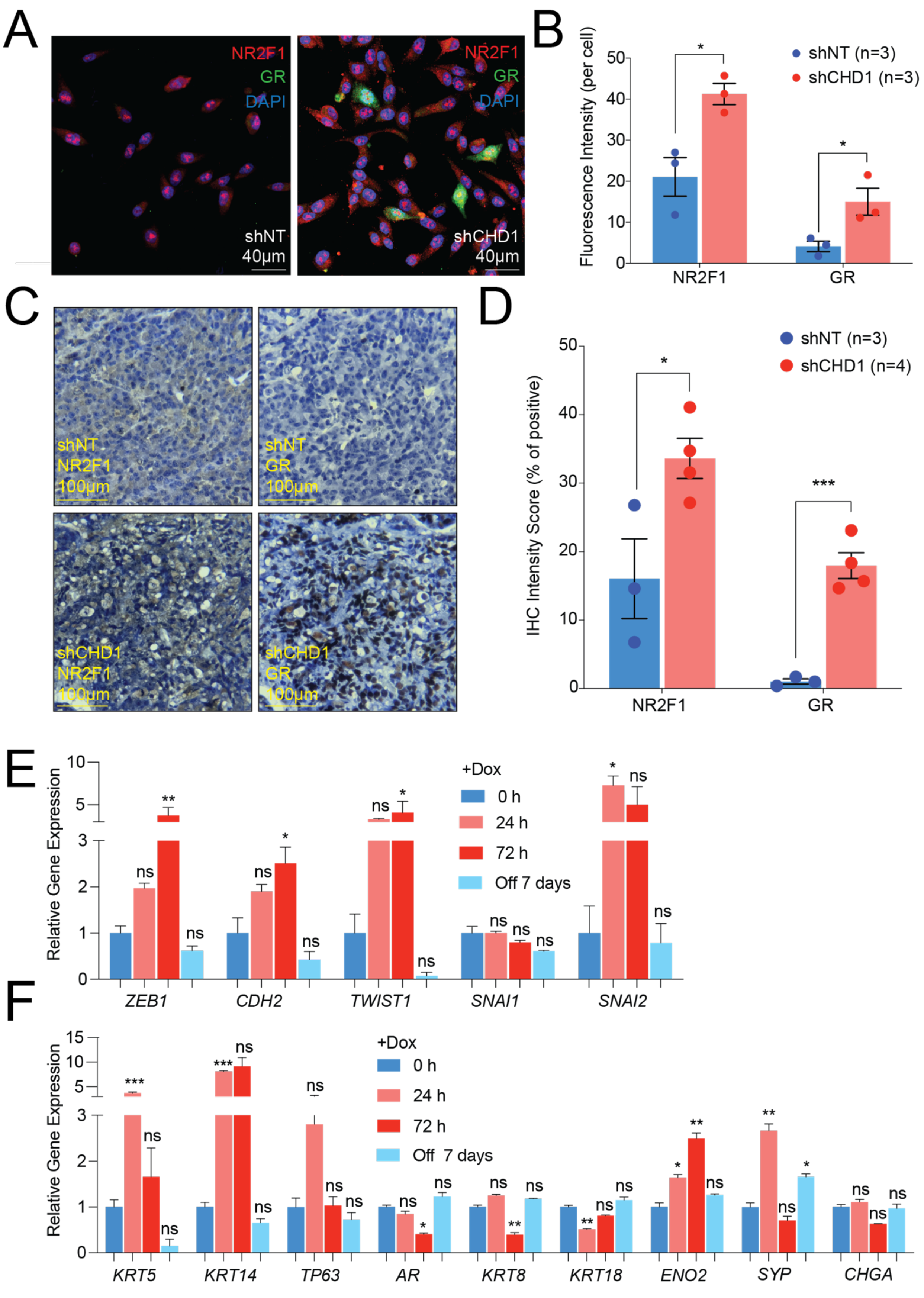

A
NR2F1
GR
DAPI
shNT
40μm
shCHD1
40μm
B
Fluorescence Intensity (per cell)
shNT (n=3)
shCHD1 (n=3)
NR2F1
GR
C
shNT
NR2F1
100μm
shNT
GR
100μm
shCHD1
NR2F1
100μm
shCHD1
GR
100μm
D
IHC Intensity Score (% of positive)
shNT (n=3)
shCHD1 (n=4)
NR2F1
GR
E
Relative Gene Expression
+Dox
0 h
24 h
72 h
Off 7 days
ZEB1
CDH2
TWIST1
SNAI1
SNAI2
F
Relative Gene Expression
+Dox
0 h
24 h
72 h
Off 7 days
KRT5
KRT14
TP63
AR
KRT8
KRT18
ENO2
SYP
CHGA

Figure S7 related to Figure 7. *CHD1* loss enhanced prostate cancer cell heterogeneity and lineage plasticity. (A) Immunofluorescence staining of NR2F1 and GR in shCHD1-XE cells. (B) Quantification of representative immunofluorescence images. Mean ± SEM is represented and p value was calculated by multiple t test. (C) Immunohistochemical staining of NR2F1 and GR on shCHD1 enzalutamide resistant tumor slides. (D) Quantification of representative immunohistochemical images. Mean ± SEM is represented and p value was calculated by multiple t test. (E-F) Relative gene expression level of the EMT genes (E) and lineage specific marker genes (F) in LNCaP/AR cells transduced with annotated inducible shRNAs at various time points. Mean ± SEM is represented and p values were calculated by two-way ANOVA, all compared to 0 hr, 3 technical replicates in each group. For all panels, **** $p<0.0001$. *** $p<0.001$. ** $p<0.01$. * $p<0.05$.

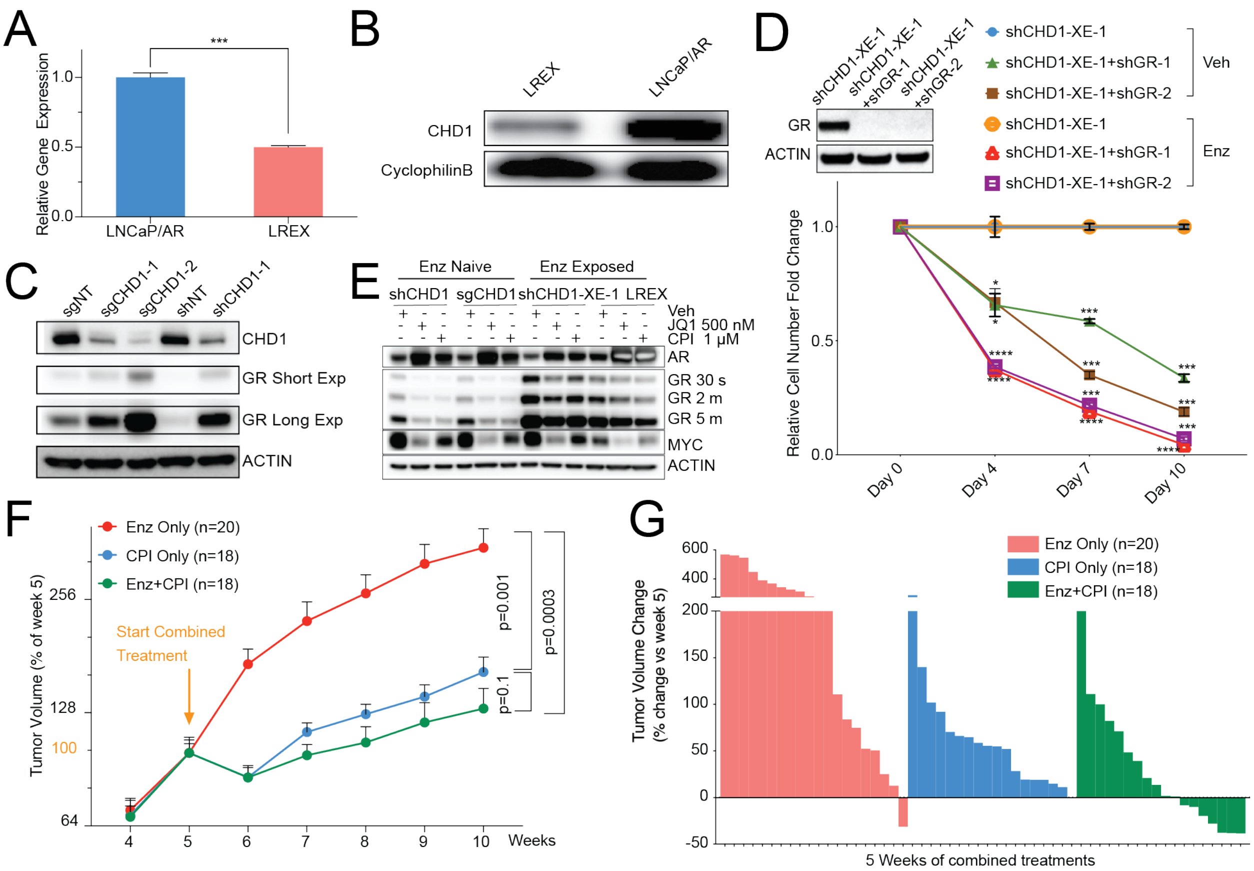

Figure S8 related to Figure 8. BET Bromodomain inhibition restores enzalutamide sensitivity. (A) Relative gene expression level of the *CHD1* in LNCaP/AR and LREX cells. Mean ± SEM is represented and p values were calculated by t test, 3 technical replicates in each group. (B) Western blot showing CHD1 protein levels in LNCaP/AR and LREX cells. (C) Western blot showing GR protein levels in LNCaP/AR cells transduced with annotated shRNAs or sgRNAs. (D) Relative cell number fold change compared to shCHD1-XE-1 group, based on the results of FACS-based competition assay. p values were calculated using two-way ANOVA. Mean ± SEM is represented and p values were calculated by two-way ANOVA, all compared to shCHD1-XE-1+Veh, 3 biological replicates in each group. Western blot showing GR protein levels in shCHD1-XE-1 cells transduced with annotated shRNAs. (E) Western blot of AR, GR, and MYC protein levels in different cell lines with enzalutamide resistance. (F) Tumor growth curve of xenografted LNCaP/AR shCHD1-XE-1 cells. All animals were treated with enzalutamide at 10 mg/kg orally 1 day after grafting. Beginning from week 5 of xenografting, animals were randomized into 3 groups and treated with enzalutamide only (Enz), CPI-0610 only (CPI) or the combination of enzalutamide plus CPI-0610. Mean ± SEM is represented and p values were calculated using two-way ANOVA. (G) Waterfall plot displaying changes in tumor size of xenografted LNCaP/AR shCHD1-XE-1 cells after 3 weeks of treatments. For (F) and (G), Enz denotes enzalutamide treatment at 10 mg/kg orally. CPI denotes CPI-0610 treatment at 60 mg/kg orally. For all panels, **** $p<0.0001$. *** $p<0.001$. ** $p<0.01$. * $p<0.05$.
